# A metal-free approach for the synthesis of amides/esters with pyridinium salts of phenacyl bromides via oxidative C–C bond cleavage

**DOI:** 10.3762/bjoc.15.182

**Published:** 2019-08-05

**Authors:** Kesari Lakshmi Manasa, Yellaiah Tangella, Namballa Hari Krishna, Mallika Alvala

**Affiliations:** 1 Department of Medicinal Chemistry, National Institute of Pharmaceutical Education and Research (NIPER), Hyderabad-500 037, India; 2 Fluoro-Agrochemicals, CSIR-Indian Institute of Chemical Technology, Hyderabad-500 007, India

**Keywords:** amidation, benzoylation, benzylamines, pyridinium salt of phenacyl bromides

## Abstract

An efficient, simple, and metal-free synthetic approach for the *N*- and *O*-benzoylation of various amines/benzyl alcohols with pyridinium salts of phenacyl bromides is demonstrated to generate the corresponding amides and esters. This protocol facilitates the oxidative cleavage of a C–C bond followed by formation of a new C–N/C–O bond in the presence of K_2_CO_3_. Various pyridinium salts of phenacyl bromides can be readily transformed into a variety of amides and esters which is an alternative method for the conventional amidation and esterification in organic synthesis. High functional group tolerance, broad substrate scope and operational simplicity are the prominent advantages of the current protocol.

## Introduction

Amidation and esterification are fundamental transformations in synthetic chemistry. The occurrence of an amide and/or ester bond is widely realized in organic molecules, proteins, natural products, pharmaceuticals, polymers and agrochemicals [1–5]. The conventional synthesis of amides involves the reaction of carboxylic acids or their derivatives such as acyl halides [6–7], anhydrides [8], esters [9], and acyl azides [10] with amines by employing coupling reagents [11]. Alternative protocols include the Staudinger–Vilarrasa reaction [12], Schmidt reaction [13–14], Beckmann rearrangement [15], aminocarbonylation of aryl halides [16], Staudinger ligation [17], rearrangement of aldoximes [18], hydration of nitriles [19], dehydrogenative coupling of primary alcohols with amines [20–21] and hydration of organonitriles to amides [22–24]. However, these traditional methods have certain disadvantages such as generation of toxic chemical waste, involvement of tedious work-up procedures and the requirement of stoichiometric amounts of coupling reagents. On the other hand, benzoylation reactions are usually performed with benzoyl chloride or benzoic anhydride which results in the formation of a substantial amount of undesired chemical waste. Moreover, these benzoic acid derivatives are commonly lachrymatory and are inconvenient to handle and store.

Over the past decades, the thermodynamic stability of C–C bonds has attracted considerable attention of the synthesis community for the reorganization of organic compound by a sequential C–C cleavage and formation of new C–N bonds in the presence of transition metal catalysts [25–34]. Tang and Jiao reported copper catalyzed C–C single bond cleavage and C–N bond formation [35]. However, the transition metal catalysts and additives employed in these transformations are toxic and restrict the substrate scope. Qandil et al. reported a transition metal-free approach for the formation of amides and esters from phenacyl bromides or chlorides [36]. Zhang et al. developed a milder and metal-free approach for the selective cleavage of C(CO)–C(α) bonds [37–40]. Kamal and co-workers also demonstrated a transition metal-free aerobic oxidative cleavage of C–C bonds of phenacyl azides employed in the construction of amides utilizing benzylamines [41].

**Scheme 1:**
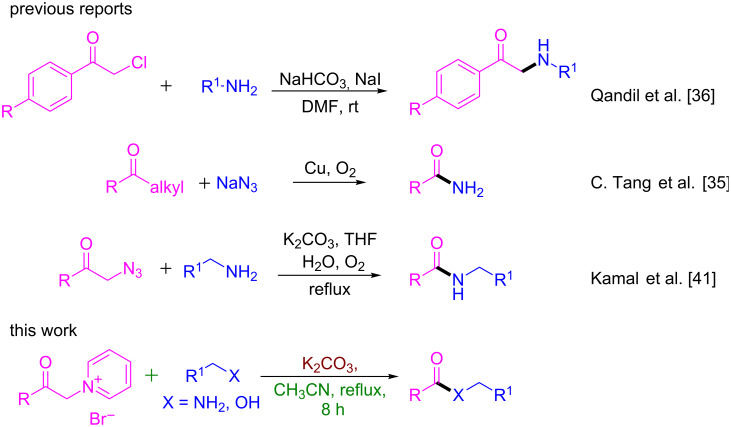
Comparison of our work with previous studies.

Herein we wish to report a metal-free C(CO)–C(α) bond cleavage of pyridinium salts of phenacyl bromides for the facile synthesis of amides and esters in the presence of a suitable amine or alcohol under basic conditions (Scheme 1). To the best of our knowledge, this is the first report for the synthesis of amides and esters from the pyridinium salts of phenacyl bromides and benzylamines/alcohols via C(CO)–C(α) bond cleavage.

## Results and Discussion

To optimize the reaction conditions for *N*- and *O*-benzoylation, a model reaction was performed between 1-(2-oxo-2-phenylethyl)pyridin-1-ium bromide and benzylamine. Initially, the reaction was carried out in acetonitrile at reflux temperature for 8 h in the presence of K_2_CO_3_ (2 equiv), which resulted in the formation of **3a** in 78% yield (Table 1, entry 2). This inspiring result led us to scrutinize the reaction conditions in detail. Increasing the amount of K_2_CO_3_ to 2.5 equiv resulted in a higher yield of 81% (Table 1, entry 3). It was observed that further increase the number of equivalents of K_2_CO_3_ had no significant effect on the improvement of the yields (Table 1, entry 4). A decrease in the yields of the product was observed when the number of equivalents of K_2_CO_3_ (1 equiv) were reduced (Table 1, entry 5), which suggests that 1 equiv of K_2_CO_3_ was insufficient to complete the reaction (Table 1, entry 5). Next, we focused our attention on screening of other bases such as KOH, NaOH, *t-*BUOK, Na_2_CO_3_, Et_3_N and DBU. It was observed that K_2_CO_3_ is superior to other organic and inorganic bases employed (Table 1, entries 6–11). To further understand the effect of solvent a series of polar and non-polar solvents such as toluene, THF, DMF and DMSO was employed in the reaction (Table 1, entries 12–15). However, there was no noticeable improvement in the yields of the desired product. Hence, the optimal conditions for the required transformation were found to be 2.5 equiv of K_2_CO_3_ in CH_3_CN at 80 ºC for 8 h.

**Table 1:** Optimization of reaction conditions.^a^

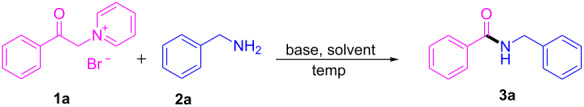					

Entry	Base (equiv)	Solvent	*T* [°C]	Time [h]	Yield^b^ [%]

1	–	CH_3_CN	80	8	–
2	K_2_CO_3_ (2)	CH_3_CN	80	8	78
3	K_2_CO_3_ (2.5)	CH_3_CN	80	8	81
4	K_2_CO_3_ (3)	CH_3_CN	80	8	62
5	K_2_CO_3_ (1.0)	CH_3_CN	80	8	35
6	KOH (2.5)	CH_3_CN	80	8	43
7	NaOH (2.5)	CH_3_CN	80	8	36
8	*t*-BUOK (2.5)	CH_3_CN	80	8	48
9	Na_2_CO_3_ (2.5)	CH_3_CN	80	8	46
10	Et_3_N (2.5)	CH_3_CN	80	8	39
11	DBU (2.5)	CH_3_CN	80	8	46
12	K_2_CO_3_ (2.5)	toluene	80	8	61
13	K_2_CO_3_ (2.5)	THF	80	8	63
14	K_2_CO_3_ (2.5)	DMF	80	8	69
15	K_2_CO_3_ (2.5)	DMSO	80	8	67

^a^Reaction conditions: **1a** (1 mmol), **2a** (1 mmol), base (2.5 mmol), solvent (5 mL), 8 h. ^b^Isolated yields.

**Scheme 2:**
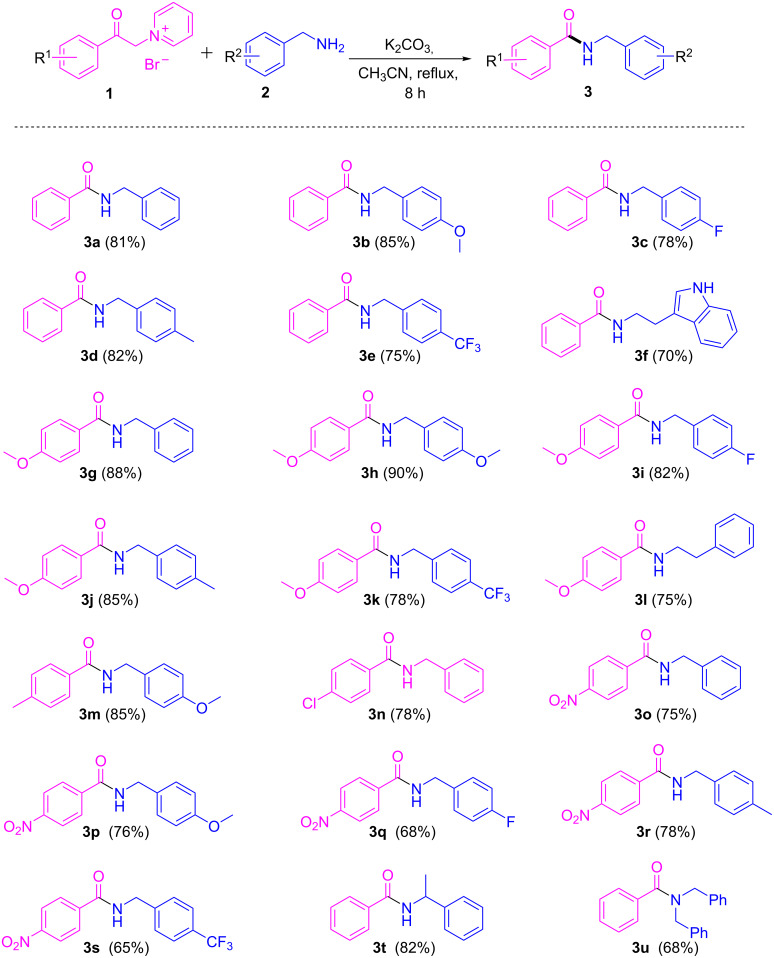
Scope of pyridinium salts and benzylamine substrates. Reaction conditions: **1** (1 mmol), **2** (1 mmol), base (2.5 mmol), solvent (5 mL), 80 °C, 8 h. Isolated yields are given in parentheses.

To understand the synthetic feasibility of the optimized reaction conditions, we extended our studies to various substituted 1-(2-oxo-2-phenylethyl)pyridin-1-ium bromides reacted with diverse benzylamines, and the results are illustrated in Scheme 2. Both electron-withdrawing and electron-donating groups on both the aryl moieties of the pyridinium salt of phenacyl bromides and benzylamines were well tolerated and delivered the corresponding products in good yields. The electron-rich pyridinium salts of phenacyl bromides reacted smoothly with the amines and furnished the corresponding products in excellent yields (**3g–l** and **3n**, Scheme 2); however, comparably lower yields were obtained with electron-withdrawing pyridinium salts of phenacyl bromides (**3m** and **3o–s**, Scheme 2). Similar results were observed with respect to the electronic nature of substituents on the benzene ring of benzylamines, where electron-donating substituents resulted in comparably higher yields than the electron-withdrawing substituents. An alkaloid amine like tryptamine was well reacted and produced the corresponding amide **3f** in a good yield. It is worth mentioning that a sterically bulkier amine like 1-phenylethanamine and a secondary amine like dibenzylamine were also found to be amenable for this transformation and delivered the corresponding amides **3t** and **3u** in good yields.

**Scheme 3:**
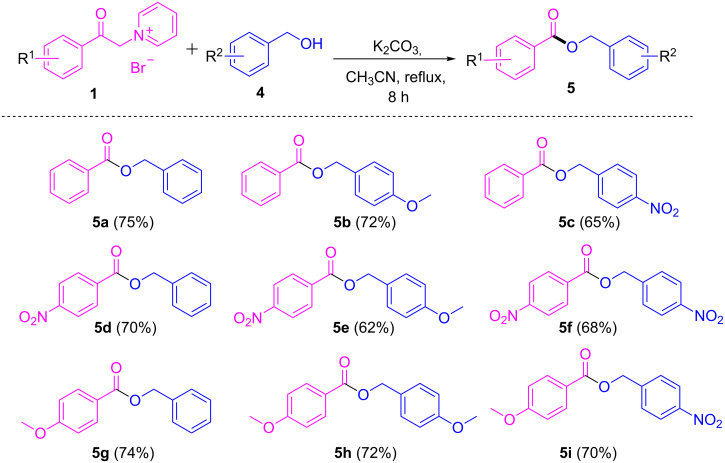
Scope of pyridinium salts and benzyl alcohol substrates. Reaction conditions: **1** (1 mmol), **4** (1 mmol), base (2.5 mmol), solvent (5 mL), 80 °C, 8 h. Isolated yields are given in parentheses.

Inspired by the above results, we thought to study the feasibility of *O*-benzoylation by utilizing benzyl alcohols under similar reaction conditions. To our pleasure, various benzyl alcohols bearing electron-withdrawing and electron-donating substituents smoothly underwent *O*-benzoylation and delivered the corresponding benzoate esters **5a–i** in good yields (Scheme 3). Similar results as for the of *N*-benzoylation were obtained with respect to the electronic nature of the substituents on 1-(2-oxo-2-phenylethyl)pyridin-1-ium bromides, where the presence of electron-withdrawing groups on the pyridinium salts of phenacyl bromides resulted in lower yields compared to electron-donating substituents.

**Scheme 4:**
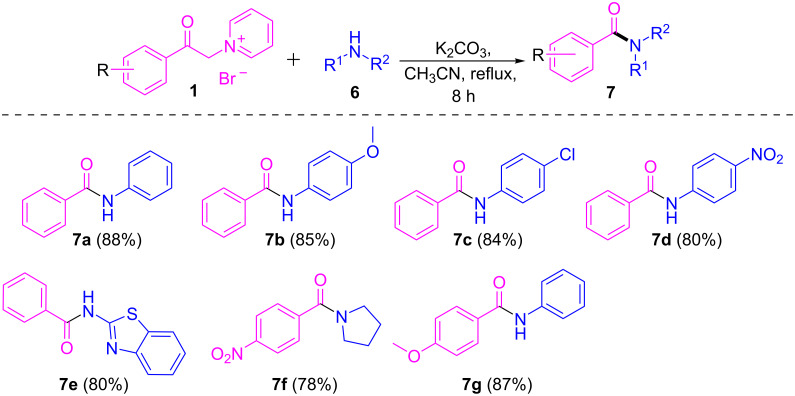
Scope of pyridinium salts, primary and secondary amine substrates. Reaction conditions: **1** (1 mmol), **6** (1 mmol), base (2.5 mmol), solvent (5 mL), 80 °C, 8 h. Isolated yields are given in parentheses.

To further investigate the synthetic applicability of the present protocol, various anilines were also utilized which yielded the corresponding amides in moderate to good yields (Scheme 4, **7a–g**). Variously substituted anilines with electron-donating and electron-withdrawing groups such as –OCH_3_, –Cl, and –NO_2_ were well tolerated under the optimized reaction conditions and delivered the corresponding products in good yields (**7b–d**). Even a heterocyclic amine such as benzo[*d*]thiazol-2-amine and a cyclic amine like pyrrolidine also reacted well and produced the corresponding amides in decent yields (**7e** and **7f**).

### Control experiments

**Scheme 5:**
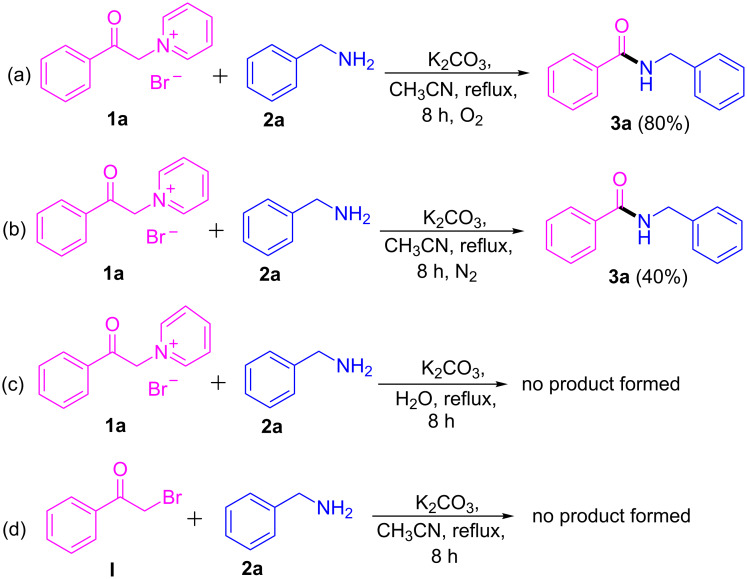
Control experiments for the oxidative cleavage of C–C bonds.

In order to elucidate the reaction mechanism for the formation of amides, a few control experiments were performed (Scheme 5). In this context we carried out the reaction under oxygen (Scheme 5a) and nitrogen atmosphere by using standard optimized conditions to afford **3a**. In the case of the reaction under a nitrogen atmosphere (Scheme 5b) the yield of the product was decreased. The product was not detected in the reaction with water as solvent (Scheme 5c). And finally, the pyridinium salt was replaced with phenacyl bromide (**I**) as a starting material and the reaction was performed under standard optimized conditions, but the expected product could not be detected (Scheme 5d).

### Plausible mechanism

**Scheme 6:**
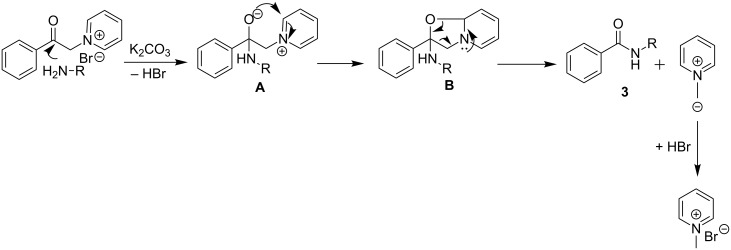
Plausible reaction mechanism for the synthesis of *N*-alkylated benzamides **3**.

A plausible reaction mechanism for the formation of amides is depicted in Scheme 6. It is assumed that initially the nucleophilic nitrogen/oxygen attacks the carbonyl carbon of the pyridinium salt of the phenacyl bromide to generate intermediate **A**. The attack of the *ortho*-position of the pyridinium moiety by the nucleophilic oxygen of intermediate **A** is then proposed to generate cyclic oxazolopyridine intermediate **B** [42–43]. The intermediate **B** then undergoes a rearrangement to afford *N*-alkylated benzamide **3** via a C–C bond cleavage with the subsequent release of pyridin-1-ium-1-ylmethanide, which further reacts with hydrogen bromide to form the 1-methylpyridin-1-ium bromide salt.

## Conclusion

In conclusion, we have developed an efficient, metal-free protocol for the *N*- and *O*-benzoylation of various amines/benzyl alcohols with pyridinium salts of phenacyl bromides for the construction of amides and esters in the presence of base via oxidative C–C bond cleavage. The promising features of this methodology include the absence of a metal catalyst, employing a simple inorganic base and operational simplicity for application in organic synthesis.

## Supporting Information

File 1 Experimental procedures, characterization data and copies of ^1^H and ^13^C NMR spectra of the compounds.
