# Supplementary material for: A metal-free approach for the synthesis of amides/esters with pyridinium salts of phenacyl bromides via oxidative C–C bond cleavage
Source: Beilstein J Org Chem. 2019 Aug 5;15:1864–71. doi: 10.3762/bjoc.15.182 (PMC6693371; doi:10.3762/bjoc.15.182)

BEILSTEIN JOURNAL OF ORGANIC CHEMISTRY

Supporting Information

for

A metal-free approach for the synthesis of amides/esters with pyridinium salts of phenacyl bromides via oxidative C–C bond cleavage

Kesari Lakshmi Manasa, Yellaiah Tangella, Namballa Hari Krishna and Mallika Alvala

Beilstein J. Org. Chem. **2019,** *15,* 1864–1871. doi:10.3762/bjoc.15.182

Experimental procedures, characterization data and copies of ^{1}H and ^{13}C NMR spectra of the compounds

License and Terms: This is a supporting information file under the terms of the Creative Commons Attribution License (http://creativecommons.org/licenses/by/4.0). Please note that the reuse, redistribution and reproduction in particular requires that the authors and source are credited.
The license is subject to the *Beilstein Journal of Organic Chemistry* terms and conditions: (https://www.beilstein-journals.org/bjoc)

Supporting information

Metal free approach for the synthesis of amides/esters with pyridinium salt of phenacyl bromides *via* oxidative C-C bond cleavage

Kesari Lakshmi Manasa,[a] Yellaiah Tangella,[b] Namballa Hari Krishna,[a] and Mallika Alvala*[a]

[a]*Department of Medicinal Chemistry, National Institute of Pharmaceutical Education and Research (NIPER), Hyderabad-500037, India*

[b]*Fluoro-Agrochemicals, CSIR-Indian Institute of Chemical Technology, Hyderabad-500007, India*

*[a]Corresponding author: Mallika Alvala, E-mail: mallikaalvala@yahoo.in

Ph: +91-9441117803

Content **Page No**

I. Material and methods S2
II. General synthetic procedure for the preparation of pyridinium salts S2
III. General synthetic procedure for the preparation of compounds (**3a–u**, **5a–i** and **7a–g**) S2–S15
IV. References S15–S16
V. Copies of NMR spectras S17–S53

1. Materials and methods

All chemicals and reagents were purchased from the commercial suppliers Alfa Aesar and Sigma-Aldrich and used without further purification. The reaction progress was monitored by Thin-layer chromatography (TLC) which was performed using pre-coated silica gel 60 F_{254} MERCK. TLC plates were visualized and analysed by exposure to UV light or iodine vapors and aqueous solution of ninhydrin. Column chromatography was performed with Merck flash silica gel with 60–120 mesh size. Melting points were determined on an electrothermal melting point apparatus and are uncorrected. FTIR spectra for the compounds were recorded on the Perkin Elmer instrument by using ATR method. Nuclear magnetic resonance spectra for ^{1}H NMR were obtained on Avance 300, 400 and 500 MHz and analyzed using Mestrenova software and the chemical shifts are reported in ppm from tetramethylsilane (0 ppm) or the solvent resonance as the internal standard ($CDCl_3$ 7.26 ppm, DMSO-d_6 2.49 ppm) and for ^{13}C NMR the chemical shifts are reported in ppm from the solvent resonance as the internal standard ($CDCl_3$ 77 ppm, DMSO-d_6 39.3 ppm). Spin multiplicities are described as s (singlet), bs (broad singlet), d (doublet), dd (doublet of doublet), ddd (doublet of doublet of doublet), t (triplet), q (quartet) and m (multiplet). Coupling constants are reported in hertz (Hz). HRMS was performed on a Varian ESI- QTOF instrument.

I. General synthetic procedure for the preparation of pyridinium salts (1)[1]

The general procedure was followed using commercially available substituted phenacyl bromides (1 equiv), pyridine (1.5 equiv) and ethyl acetate (30 mL) to provide pure salt as a white solid with the same physicochemical properties as described in the literature.

II. General synthetic procedure for the preparation of compounds (3a–u, 5a–i and 7a–g)

To a stirred solution of a pyridinium salt of phenacyl bromide (1 mmol) and the benzylamine/benzyl alcohol/amine (1 mmol) was added in CH_3CN and allowed to stir. K_2CO_3 was added to the reaction mixture and refluxed for 8 h. The reaction progress was monitored by using TLC. After complete consumption of the starting materials, the CH_3CN was evaporated under reduced pressure and the crude reaction mixture was diluted with EtOAc (35 mL) and washed with water (10 mL) and brine (10 mL), then dried over anhydrous sodium sulfate and concentrated to yield the crude amide, which was purified by using silica gel column chromatography.

N-Benzylbenzamide (**3a**)[2]

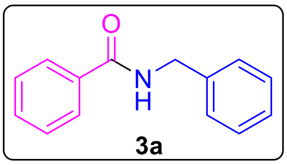

White solid; R_f = 0.42 (Ethyl acetate/*n*-Hexane, 3:7); 81% yield; mp: 106–108 °C (lit: 106-108 °C); ^{1}H NMR (400 MHz, $CDCl_3$) δ: 7.78 (dd, J = 5.3, 3.3 Hz, 2H), 7.52–7.46 (m, 1H), 7.41 (t, J = 7.6 Hz, 2H), 7.35 (s, 2H), 7.34 (s, 2H), 7.32–7.26 (m, 1H), 6.52 (s, 1H), 4.63 (d, J = 5.7 Hz, 2H); ^{13}C NMR (126 MHz, $CDCl_3$) δ: 167.5, 138.3, 134.4, 131.6, 128.8, 128.6, 127.9, 127.6, 127.0, 44.2; HRMS (ESI): *m/z* calcd for $C_{14}H_{14}NO$: 212.1069; found: 212.1061 $[M+H]^+$.

N-(4-Methoxybenzyl)benzamide (**3b**)[3]

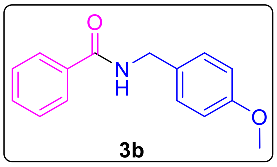

White solid; R_f = 0.38 (Ethyl acetate/*n*-Hexane, 3:7); 85% yield; mp: 94–96 °C (lit: 96-98 °C); ^{1}H NMR (400 MHz, $CDCl_3$) δ: 7.80–7.75 (m, 2H), 7.48 (t, J = 7.3 Hz, 1H), 7.41 (t, J = 7.5 Hz, 2H), 7.30–7.24 (m, 2H), 6.87 (d, J = 8.6 Hz, 2H), 6.46 (s, 1H), 4.56 (d, J = 5.5 Hz, 2H), 3.79 (s, 3H); ^{13}C NMR (101 MHz, $CDCl_3$) δ: 167.4, 159.1, 134.5, 131.5, 130.4, 129.3, 128.6, 127.0, 114.1, 55.3, 43.6; HRMS (ESI): *m/z* calcd for $C_{15}H_{16}NO_2$: 242.1175; found: 242.1170 $[M+H]^+$.

N-(4-Fluorobenzyl)benzamide (**3c**)[4]

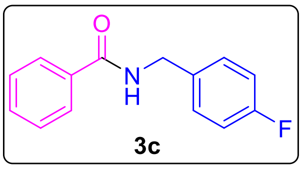

White solid; R_f = 0.34 (Ethyl acetate/*n*-Hexane, 3:7); 78% yield; mp: 112–114 °C (lit: 110-112 °C); ^{1}H NMR (400 MHz, $CDCl_3$) δ: 7.78 (dd, J = 5.3, 3.3 Hz, 2H), 7.53–7.48 (m, 1H), 7.46–7.40 (m, 2H), 7.36–7.29 (m, 2H), 7.07–6.99 (m, 2H), 6.46 (s, 1H), 4.61 (d, J = 5.8 Hz, 2H); ^{13}C NMR (101 MHz, $CDCl_3$) δ: 162.1 (d, $J_{C\text{-}F}$ = 245.4 Hz), 159.0, 134.4, 131.4, 129.2, 128.5, 127.0 (d, $J_{C\text{-}F}$ = 6.8 Hz), 115.4 (d, $J_{C\text{-}F}$ = 21.5 Hz), 114.1, 43.5; HRMS (ESI): *m/z* calcd for $C_{14}H_{13}FNO$: 230.0975; found: 230.0968 $[M+H]^+$.

N-(4-Methylbenzyl)benzamide (**3d**)[4]

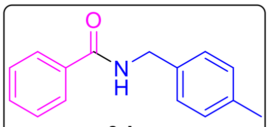

3d

White solid; R_f = 0.34 (Ethyl acetate/*n*-Hexane, 3:7); 82% yield; mp: 116–118 °C (lit: 117-119 ºC); ^{1}H NMR (300 MHz, $CDCl_3$) δ: 7.78 (d, J = 7.2 Hz, 2H), 7.54–7.46 (m, 1H), 7.42 (t, J = 7.3 Hz, 2H), 7.25 (d, J = 5.9 Hz, 2H), 7.16 (d, J = 7.8 Hz, 2H), 6.37 (s, 1H), 4.61 (d, J = 5.5 Hz, 2H), 2.35 (s, 3H); ^{13}C NMR (126 MHz, $CDCl_3$) δ: 167.4, 137.3, 135.2, 134.5, 131.5, 129.4,128.6, 127.9, 127.0, 43.9, 21.2; HRMS (ESI): *m/z* calcd for $C_{15}H_{16}NO$: 226.1226; found: 226.1219 $[M+H]^+$.

N-(4-(Trifluoromethyl)benzyl)benzamide (**3e**)[5, 6]

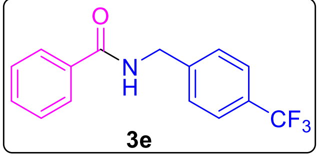

3e

White solid; R_f = 0.36 (Ethyl acetate/*n*-Hexane, 3:7); 75% yield; mp: 142–144 °C (lit: 140–141 ºC); ^{1}H NMR (400 MHz, $CDCl_3$) δ: 7.80 (d, J = 7.4 Hz, 2H), 7.59 (d, J = 8.0 Hz, 2H), 7.52 (t, J = 7.3 Hz, 1H), 7.46 (d, J = 4.0 Hz, 2H), 7.43 (t, J = 5.5 Hz, 2H), 6.65 (s, 1H), 4.69 (d, J = 5.8 Hz, 2H); ^{13}C NMR (101 MHz, $CDCl_3$) δ: 167.7, 142.5, 134.1, 131.8, 130.0, 128.7, 128.1 (q, $J_{C\text{-}F}$ = 25.5 Hz), 127.1, 125.7 (q, $J_{C\text{-}F}$ = 3.2 Hz), 124.2 (q, $J_{C\text{-}F}$ = 272.2 Hz), 43.5; HRMS (ESI): *m/z* calcd for $C_{15}H_{13}F_3NO$: 280.0943; found: 280.0940 $[M+H]^+$.

N-(2-(1*H*-Indol-3-yl)ethyl)benzamide (**3f**)[7]

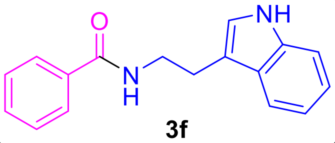

3f

Off White solid; R_f = 0.15 (Ethyl acetate/*n*-Hexane, 3:7); 70% yield; mp: 134–136 °C (lit: 135–137 ºC); ^{1}H NMR (400 MHz, $CDCl_3$) δ: 8.15 (s, 1H), 7.68 (s, 1H), 7.67–7.64 (m, 2H), 7.46 (ddd, J = 6.6, 3.9, 1.3 Hz, 1H), 7.40 (d, J = 1.5 Hz, 1H), 7.39–7.35 (m, 2H), 7.25–7.19 (m, 1H), 7.17–7.10 (m, 1H), 7.07 (d, J = 2.2 Hz, 1H), 6.23 (s, 1H), 3.81 (dd, J = 12.5, 6.6 Hz, 2H), 3.11 (t, J = 6.5 Hz, 2H); ^{13}C NMR (101 MHz, $CDCl_3$) δ: 167.6, 136.5, 134.7, 131.4, 128.6, 127.4,

126.9, 122.3, 122.2, 119.6, 118.8, 113.0, 111.4, 40.4, 25.4; HRMS (ESI): *m/z* calcd for $C_{17}H_{17}N_2O$: 265.1335; found: 265.1325 $[M+H]^+$.

N-Benzyl-4-methoxybenzamide (**3g**)[8, 9]

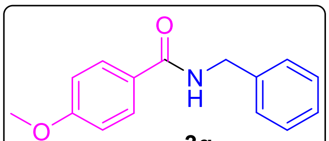

3g

White solid; R_f = 0.26 (Ethyl acetate/*n*-Hexane, 3:7); 88% yield; mp: 132–134 °C (lit: 128–130 °C); ^{1}H NMR (400 MHz, $CDCl_3$) δ: 7.79–7.73 (m, 2H), 7.35 (d, *J* = 4.4 Hz, 4H), 7.30 (dd, *J* = 8.4, 3.9 Hz, 1H), 6.93–6.89 (m, 2H), 6.35 (s, 1H), 4.63 (d, *J* = 5.6 Hz, 2H), 3.84 (s, 3H); ^{13}C NMR (101 MHz, $CDCl_3$) δ: 167.0, 162.3, 138.5, 128.9, 128.8, 128.0, 127.6, 126.7, 113.8, 55.5, 44.1; HRMS (ESI): *m/z* calcd for $C_{15}H_{16}NO_2$: 242.1175; found: 242.1170 $[M+H]^+$.

4-Methoxy-*N*-(4-methoxybenzyl)benzamide (**3h**)[10]

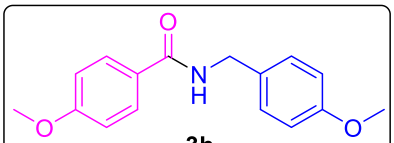

3h

White solid; R_f = 0.15 (Ethyl acetate/*n*-Hexane, 3:7); 90% yield; mp: 158–160 °C (lit: 160–162 °C); ^{1}H NMR (400 MHz, $CDCl_3$) δ: 7.74 (d, *J* = 8.8 Hz, 2H), 7.28 (d, *J* = 8.6 Hz, 2H), 6.91 (d, *J* = 8.8 Hz, 2H), 6.88 (d, *J* = 8.6 Hz, 2H), 6.27 (s, 1H), 4.56 (d, *J* = 5.5 Hz, 2H), 3.84 (s, 3H), 3.80 (s, 3H); ^{13}C NMR (101 MHz, $CDCl_3$) δ: 166.9, 162.2, 159.1, 130.6, 129.3, 128.8, 126.8, 114.1, 113.8, 55.4, 55.3, 43.6; HRMS (ESI): *m/z* calcd for $C_{16}H_{18}NO_3$: 272.1281; found: 272.1276 $[M+H]^+$.

N-(4-Fluorobenzyl)-4-methoxybenzamide (**3i**)[10]

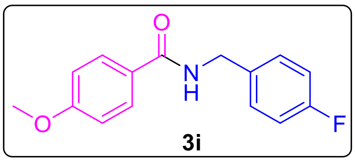

3i

White solid; R_f = 0.23 (Ethyl acetate/*n*-Hexane, 3:7); 82% yield; mp: 132–134 °C (lit: 134–136 °C); ^{1}H NMR (400 MHz, $CDCl_3$) δ: 7.75 (d, *J* = 8.7 Hz, 2H), 7.31 (dd, *J* = 8.0, 5.5 Hz, 2H), 7.02 (t, *J* = 8.6 Hz, 2H), 6.91 (d, *J* = 8.7 Hz, 2H), 6.43 (s, 1H), 4.58 (d, *J* = 5.6 Hz, 2H),

3.84 (s, 3H); ^{13}C NMR (101 MHz, $CDCl_3$+DMSO–d_6) δ: 166.5, 161.4 (d, $J_{C\text{-}F}$ = 244.6 Hz), 161.6, 134.8, 128.9 (d, $J_{C\text{-}F}$ = 8.0 Hz), 128.8, 126.3, 114.7 (d, $J_{C\text{-}F}$ = 21.3 Hz), 113.1, 55.0, 42.4; HRMS (ESI): *m/z* calcd for $C_{15}H_{15}FNO_2$: 260.1081; found: 260.1075 $[M+H]^+$.

4-Methoxy-*N*-(4-methylbenzyl)benzamide (**3j**)

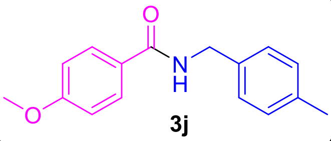

3j

White solid; R_f = 0.21 (Ethyl acetate/*n*-Hexane, 3:7); 85% yield; mp: 167–169 °C; FT-IR (cm^{-1}): 3273.3, 3004.9, 2970.7, 2955.5, 2836.1, 1738.8, 1633.4, 1603.5; ^{1}H NMR (400 MHz, $CDCl_3$) δ: 7.77–7.72 (m, 2H), 7.24 (d, J = 8.0 Hz, 2H), 7.15 (d, J = 7.9 Hz, 2H), 6.93–6.88 (m, 2H), 6.30 (s, 1H), 4.58 (d, J = 5.6 Hz, 2H), 3.84 (s, 3H), 2.34 (s, 3H); ^{13}C NMR (101 MHz, $CDCl_3$) δ: 166.9, 162.2, 137.3, 135.4, 129.5, 128.8, 128.0, 126.8, 113.8, 55.5, 43.9, 21.2; HRMS (ESI): *m/z* calcd for $C_{16}H_{18}NO_2$: 256.1332; found: 256.1327 $[M+H]^+$.

4-Methoxy-*N*-(4-(trifluoromethyl)benzyl)benzamide (**3k**)

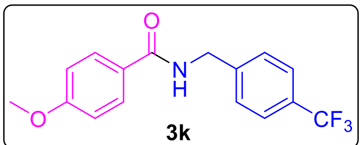

3k

White solid; R_f = 0.23 (Ethyl acetate/*n*-Hexane, 3:7); 78% yield; mp: 188–190 °C; FT-IR (cm^{-1}): 3298.2, 2845.8, 1633.9, 1619.1, 1602.5, 1548.1, 1461.1, 1442.5; ^{1}H NMR (500 MHz, $CDCl_3$) δ: 7.79–7.75 (m, 2H), 7.60 (d, J = 8.1 Hz, 2H), 7.46 (d, J = 8.0 Hz, 2H), 6.95–6.91 (m, 2H), 6.46 (s, 1H), 4.69 (d, J = 5.9 Hz, 2H), 3.85 (s, 3H); ^{13}C NMR (75 MHz, $CDCl_3$+DMSO–d_6) δ: 165.2, 160.6, 143.0, 127.9, 126.6 (q, $J_{C\text{-}F}$ = 24.9 Hz), 125.1, 124.9 (q, $J_{C\text{-}F}$ = 271.7 Hz), 123.7 (q, $J_{C\text{-}F}$ = 3.4 Hz), 112.0, 53.9, 41.4; HRMS (ESI): *m/z* calcd for $C_{16}H_{15}F_3NO_2$: 310.1049; found: 310.1046 $[M+H]^+$.

4-Methoxy-*N*-phenethylbenzamide (**3l**)[10]

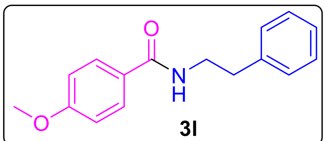

3l

White solid; R_f = 0.21 (Ethyl acetate/*n*-Hexane, 3:7); 75% yield; mp: 116–118 °C (lit: 117-118 °C); ^{1}H NMR (400 MHz, $CDCl_3$) δ: 7.68–7.63 (m, 2H), 7.35–7.30 (m, 2H), 7.24 (d, J = 7.9 Hz, 3H), 6.92–6.87 (m, 2H), 6.05 (s, 1H), 3.83 (s, 3H), 3.70 (dd, J = 12.8, 6.8 Hz, 2H), 2.93 (t, J = 6.9 Hz, 2H); ^{13}C NMR (101 MHz, $CDCl_3$) δ: 155.9, 141.5, 127.9, 122.5, 122.4, 122.3, 120.6, 120.2, 102.6, 47.5, 39.7, 34.4; HRMS (ESI): *m/z* calcd for $C_{16}H_{18}NO_2$: 256.1332; found: 256.1347 $[M+H]^+$.

N-(4-Methoxybenzyl)-4-methylbenzamide (**3m**)[11]

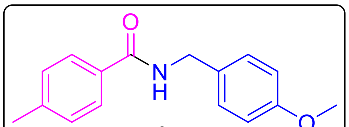

3m

White solid; R_f = 0.24 (Ethyl acetate/*n*-Hexane, 3:7); 85% yield; mp: 114–116 °C (lit: 115-117 °C); ^{1}H NMR (400 MHz, $CDCl_3$) δ: 7.67 (d, J = 8.1 Hz, 2H), 7.28 (d, J = 8.6 Hz, 2H), 7.22 (d, J = 7.9 Hz, 2H), 6.88 (d, J = 8.6 Hz, 2H), 6.33 (s, 1H), 4.57 (d, J = 5.5 Hz, 2H), 3.80 (s, 3H), 2.38 (s, 3H); ^{13}C NMR (101 MHz, $CDCl_3$) δ: 167.3, 159.2, 142.0, 131.7, 130.5, 129.4, 129.3, 127.0, 114.2, 55.4, 43.6, 21.5; HRMS (ESI): *m/z* calcd for $C_{16}H_{18}NO_2$: 256.1332; found: 256.1339 $[M+H]^+$.

N-Benzyl-4-chlorobenzamide (**3n**)[11]

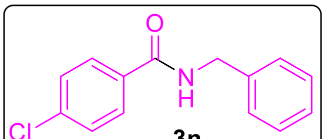

3n

Off White solid; R_f = 0.45 (Ethyl acetate/*n*-Hexane, 3:7); 78% yield; mp: 162–164 °C (lit: 164–165 °C); ^{1}H NMR (400 MHz, $CDCl_3$) δ: 7.75–7.70 (m, 2H), 7.42–7.38 (m, 2H), 7.37 (t, J = 1.5 Hz, 1H), 7.35 (d, J = 2.8 Hz, 3H), 7.34–7.28 (m, 1H), 6.38 (s, 1H), 4.63 (d, J = 5.6 Hz, 2H); ^{13}C NMR (75 MHz, $CDCl_3$+DMSO–d_6) δ: 165.8, 138.5, 136.6, 132.5, 128.5, 127.9, 127.8, 127.1, 126.6, 43.1; HRMS (ESI): *m/z* calcd for $C_{14}H_{13}ClNO$: 246.0680; found: 246.0682 $[M+H]^+$.

N-Benzyl-4-nitrobenzamide (**3o**)[9, 12]

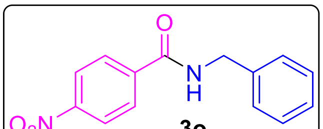

3o

Cream colour solid; R_f = 0.27 (Ethyl acetate/*n*-Hexane, 3:7); 75% yield; mp: 134–136 °C (lit: 134–137°C); ^{1}H NMR (400 MHz, $CDCl_3$) δ: 8.27 (d, J = 8.6 Hz, 2H), 7.95 (d, J = 8.6 Hz, 2H), 7.41–7.29 (m, 5H), 6.54 (s, 1H), 4.66 (d, J = 5.6 Hz, 2H); ^{13}C NMR (101 MHz, $CDCl_3$) δ: 164.8, 148.7, 139.7, 138.1, 128.3, 128.0, 127.1, 126.7, 122.7, 43.2; HRMS (ESI): *m/z* calcd for $C_{14}H_{13}N_2O_3$: 257.0920; found: 257.0914 $[M+H]^+$.

N-(4-Methoxybenzyl)-4-nitrobenzamide (**3p**)[9, 13]

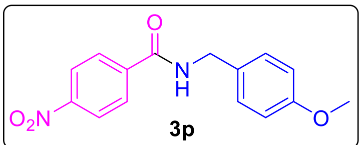

3p

White solid; R_f = 0.21 (Ethyl acetate/*n*-Hexane, 3:7); 76% yield; mp: 136–138 °C (lit: 136-137 °C); ^{1}H NMR (400 MHz, $CDCl_3$) δ: 8.26 (d, J = 8.2 Hz, 2H), 7.93 (d, J = 8.2 Hz, 2H), 7.28 (d, J = 8.4 Hz, 2H), 6.89 (d, J = 8.0 Hz, 2H), 6.50 (s, 1H), 4.58 (d, J = 4.9 Hz, 2H), 3.80 (s, 3H); ^{13}C NMR (101 MHz, $CDCl_3$) δ: 165.3, 159.4, 149.6, 140.1, 129.6, 129.4, 128.3, 123.9, 114.3, 55.4, 44.0; HRMS (ESI): *m/z* calcd for $C_{15}H_{15}N_2O_4$: 287.1026; found: 287.1021 $[M+H]^+$.

N-(4-Fluorobenzyl)-4-nitrobenzamide (**3q**)[14]

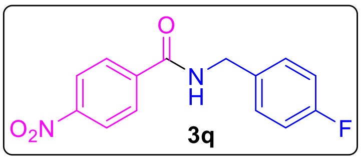

3q

Cream colour solid; R_f = 0.26 (Ethyl acetate/*n*-Hexane, 3:7); 68% yield; mp:128–130 °C (lit: 129-132 °C); ^{1}H NMR (400 MHz, $CDCl_3$) δ: 8.28 (d, J = 8.7 Hz, 2H), 7.95 (d, J = 8.7 Hz, 2H), 7.34 (dd, J = 8.3, 5.4 Hz, 2H), 7.05 (t, J = 8.6 Hz, 2H), 6.51 (s, 1H), 4.63 (d, J = 5.6 Hz, 2H); ^{13}C NMR (101 MHz, $CDCl_3$+DMSO–d_6) δ: 164.4, 161.0 (d, $J_{C\text{-}F}$ = 244.7 Hz), 148.5, 139.3, 133.9 (d, $J_{C\text{-}F}$ = 2.4 Hz), 128.6 (d, $J_{C\text{-}F}$ = 7.9 Hz), 128.0, 122.5, 114.4 (d, $J_{C\text{-}F}$ = 21.3 Hz), 42.2; HRMS (ESI): *m/z* calcd for $C_{14}H_{12}FN_2O_3$: 275.0826; found: 275.0823 $[M+H]^+$.

N-(4-Methylbenzyl)-4-nitrobenzamide (**3r**)[15]

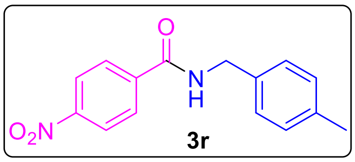

3r

White solid; R_f = 0.30 (Ethyl acetate/*n*-Hexane, 3:7); 78% yield; mp: 152–154 °C (lit: 154-155 °C); ^{1}H NMR (400 MHz, $CDCl_3$) δ: 8.26 (d, J = 8.6 Hz, 2H), 7.93 (d, J = 8.6 Hz, 2H), 7.24 (s, 2H), 7.18 (d, J = 7.8 Hz, 2H), 6.49 (s, 1H), 4.61 (d, J = 5.2 Hz, 2H), 2.35 (s, 3H); ^{13}C NMR (101 MHz, $CDCl_3$+DMSO–d_6) δ: 165.2, 149.1, 140.0, 136.8, 135.1, 129.0, 128.5, 127.6, 123.2, 43.5, 20.8; HRMS (ESI): *m/z* calcd for $C_{15}H_{15}N_2O_3$: 271.1077; found: 271.1072 $[M+H]^+$.

4-Nitro-*N*-(4-(trifluoromethyl)benzyl)benzamide (**3s**)

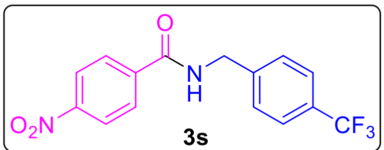

3s

White solid; R_f = 0.26 (Ethyl acetate/*n*-Hexane, 3:7); 65% yield; mp: 166–168 °C; FT-IR (cm^{-1}): 3285.3, 2970.7, 1738.7, 1644.2, 1621.1, 1599.6, 1547.8, 1521.7; ^{1}H NMR (400 MHz, $CDCl_3$) δ: 8.29 (d, J = 8.5 Hz, 2H), 7.96 (d, J = 8.5 Hz, 2H), 7.62 (d, J = 7.8 Hz, 2H), 7.48 (d, J = 7.7 Hz, 2H), 6.64 (s, 1H), 4.73 (d, J = 5.5 Hz, 2H); ^{13}C NMR (101 MHz, $CDCl_3$+ DMSO–d_6) δ: 165.23, 149.15, 142.68, 139.64, 129.13, 128.87, 128.57, 127.74, 126.13 (q, J_{C-F} = 245.6 Hz), 123.12 (q, J_{C-F} = 2.7 Hz), 43.14; HRMS (ESI): *m/z* calcd for $C_{15}H_{12}F_3N_2O_3$: 325.0794; found: 325.0794 $[M+H]^+$.

N-(1-Phenylethyl)benzamide (**3t**)[16]

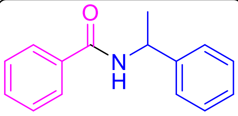

3t

White solid; R_f = 0.38 (Ethyl acetate/*n*-Hexane, 3:7); 82% yield; mp: 118–120 °C (lit: 119-120 °C); ^{1}H NMR (400 MHz, $CDCl_3$) δ: 7.80–7.75 (m, 2H), 7.52–7.46 (m, 1H), 7.45–7.42 (m, 2H), 7.42–7.40 (m, 1H), 7.38 (dd, J = 5.1, 1.7 Hz, 2H), 7.37–7.33 (m, 1H), 7.29 (ddd, J = 7.0, 3.9, 1.5 Hz, 1H), 6.32 (s, 1H), 5.35 (d, J = 7.0 Hz, 1H), 1.62 (d, J = 4.4 Hz, 3H); ^{13}C NMR (101 MHz, $CDCl_3$) δ: 166.7, 143.2, 134.7, 131.6, 128.8, 128.6, 127.6, 127.0, 126.3, 49.3, 21.8; HRMS (ESI): *m/z* calcd for $C_{15}H_{16}NO$: 226.1226; found: 226.1233 $[M+H]^+$.

N,*N*-Dibenzylbenzamide (**3u**)[17]

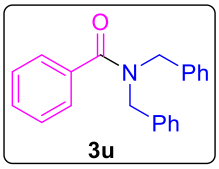

White solid; R_f = 0.51 (Ethyl acetate/*n*-Hexane, 3:7); 68% yield; mp: 108–110 °C (lit: 108-109 °C); ^{1}H NMR (500 MHz, $CDCl_3$) δ: 7.51–7.49 (m, 2H), 7.40–7.37 (m, 4H), 7.36 (t, J = 5.5 Hz, 4H), 7.32 (s, 1H), 7.30 (d, J = 7.2 Hz, 2H), 7.15 (s, 2H), 4.71 (s, 2H), 4.41 (s, 2H); ^{13}C NMR (101 MHz, $CDCl_3$) δ: 172.3, 137.0, 136.5, 136.2, 129.7, 128.9, 128.8, 128.6, 128.5, 127.6, 127.1, 126.8, 51.6, 46.9; HRMS (ESI): *m/z* calcd for $C_{21}H_{20}NO$: 302.1539; found: 302.1566 $[M+H]^+$.

Benzyl benzoate (**5a**)

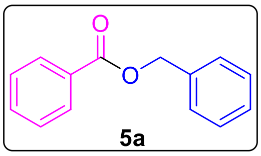

Yellow liquid; R_f = 0.53 (Ethyl acetate/*n*-Hexane, 3:7); 75% yield; ^{1}H NMR (500 MHz, $CDCl_3$) δ: 8.08 (dd, J = 8.4, 1.3 Hz, 2H), 7.58– 7.54 (m, 1H), 7.47–7.44 (m, 2H), 7.44–7.41 (m, 2H), 7.41–7.37 (m, 2H), 7.35 (dt, J = 9.7, 4.3 Hz, 1H), 5.37 (s, 2H); ^{13}C NMR (101 MHz, $CDCl_3$) δ: 166.5, 136.1, 133.1, 130.2, 129.8, 128.7, 128.4, 128.3, 128.2, 66.8; HRMS (ESI): *m/z* calcd for $C_{14}H_{13}O_2$: 213.0910; found: 213.0907 $[M+H]^+$.

4-Methoxybenzyl benzoate (**5b**)

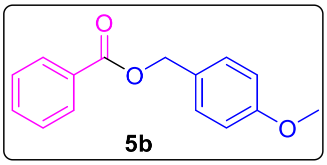

Colourless Liquid; R_f = 0.52 (Ethyl acetate/*n*-Hexane, 3:7); 72% yield; ^{1}H NMR (400 MHz, $CDCl_3$) δ: 8.06 (ddd, J = 7.0, 3.2, 1.5 Hz, 2H), 7.55 (ddd, J = 6.9, 4.1, 1.4 Hz, 1H), 7.45–7.41 (m, 2H), 7.41–7.37 (m, 2H), 6.94–6.90 (m, 2H), 5.30 (s, 2H), 3.82 (s, 3H); ^{13}C NMR (101 MHz, $CDCl_3$) δ: 166.6, 159.7, 133.0, 130.3, 130.1, 129.7, 128.4, 128.2, 114.0, 66.6, 55.4; HRMS (ESI): *m/z* calcd for $C_{15}H_{15}O_3$: 243.1015; found: 243.1013 $[M+H]^+$.

4-Nitrobenzyl benzoate (**5c**)[18]

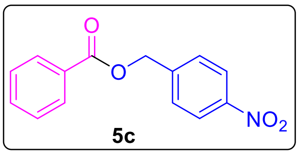

White solid; R_f = 0.62 (Ethyl acetate/*n*-Hexane, 3:7); 65% yield; mp: 91–93 °C (lit: 88-89 °C); ^{1}H NMR (400 MHz, $CDCl_3$) δ: 8.25 (d, J = 8.7 Hz, 2H), 8.11–8.07 (m, 2H), 7.60 (dd, J = 10.1, 4.9 Hz, 3H), 7.48 (t, J = 7.7 Hz, 2H), 5.47 (s, 2H); ^{13}C NMR (126 MHz, $CDCl_3$) δ: 166.2, 147.8, 143.4, 133.6, 129.8, 129.6, 128.7, 128.4, 124.0, 65.3; HRMS (ESI): *m/z* calcd for $C_{14}H_{12}NO_4$: 258.0760; found: 258.0753 $[M+H]^+$.

Benzyl 4-nitrobenzoate (**5d**)[19]

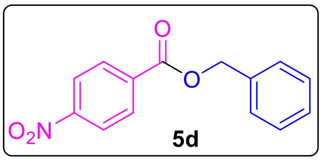

White solid; R_f = 0.34 (Ethyl acetate/*n*-Hexane, 3:7); 70% yield; mp: 84–86 °C (lit: 82-83 °C); ^{1}H NMR (500 MHz, $CDCl_3$) δ: 8.28 (d, J = 8.9 Hz, 2H), 8.24 (d, J = 8.9 Hz, 2H), 7.46 (d, J = 7.0 Hz, 2H), 7.44–7.36 (m, 3H), 5.41 (s, 2H); ^{13}C NMR (101 MHz, $CDCl_3$) δ: 164.6, 150.7, 135.6, 135.3, 130.9, 128.8, 128.8, 128.5, 123.7, 67.8; HRMS (ESI): *m/z* calcd for $C_{14}H_{12}NO_4$: 258.0760; found: 258.0758 $[M+H]^+$.

4-Methoxybenzyl 4-nitrobenzoate (**5e**)

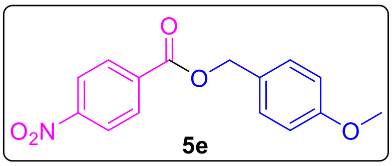

White solid; R_f = 0.62 (Ethyl acetate/*n*-Hexane, 3:7); 62% yield; mp: 103–105 °C; FT-IR (cm^{-1}): 3004.7, 2970.7, 2841.6, 1738.1, 1720.8, 1609.9, 1586.2, 1523.3; ^{1}H NMR (400 MHz, $CDCl_3$) δ: 8.27 (d, J = 8.9 Hz, 2H), 8.21 (d, J = 8.9 Hz, 2H), 7.40 (d, J = 8.6 Hz, 2H), 6.93 (d, J = 8.6 Hz, 2H), 5.34 (s, 2H), 3.83 (s, 3H); ^{13}C NMR (101 MHz, $CDCl_3$) δ: 164.7, 160.0, 150.6, 135.7, 130.9, 130.5, 127.4, 123.6, 114.2, 67.6, 55.4; HRMS (ESI): *m/z* calcd for $C_{15}H_{14}NO_5$: 288.0866; found: 288.0855 $[M+H]^+$.

4-Nitrobenzyl 4-nitrobenzoate (**5f**)[20]

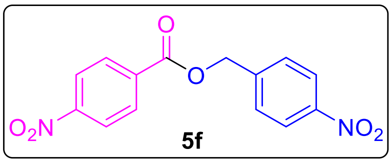

White solid; R_f = 0.50 (Ethyl acetate/*n*-Hexane, 3:7); 68% yield; mp: 164–166 °C (lit: 168-170 °C); ^{1}H NMR (400 MHz, $CDCl_3$) δ: 8.32 (d, J = 8.7 Hz, 2H), 8.26 (t, J = 8.3 Hz, 4H), 7.62 (d, J = 8.4 Hz, 2H), 5.50 (s, 2H); ^{13}C NMR (75 MHz, $CDCl_3$+DMSO–d_6) δ: 163.9, 150.4, 147.2, 143.1, 134.7, 130.8, 128.7, 123.8, 123.5, 65.7; HRMS (ESI): *m/z* calcd for $C_{14}H_{11}N_2O_6$: 303.0611; found: 303.0609 $[M+H]^+$.

Benzyl 4-methoxybenzoate (**5g**)

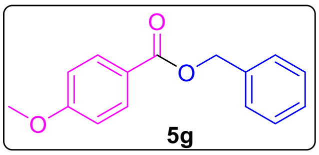

Colourless Liquid: R_f = 0.54 (Ethyl acetate/*n*-Hexane, 3:7); 74% yield; ^{1}H NMR (400 MHz, $CDCl_3$) δ: 8.06–7.98 (m, 2H), 7.44 (dd, J = 8.4, 1.2 Hz, 2H), 7.41–7.36 (m, 2H), 7.35 (dd, J = 5.0, 3.5 Hz, 1H), 6.94–6.89 (m, 2H), 5.34 (s, 2H), 3.86 (s, 3H); ^{13}C NMR (101 MHz, $CDCl_3$) δ: 166.3, 163.5, 136.4, 131.8, 128.6, 128.2, 128.2, 122.6, 113.7, 66.5, 55.5; HRMS (ESI): *m/z* calcd for $C_{15}H_{15}O_3$: 243.1015; found: 243.1014 $[M+H]^+$.

4-Methoxybenzyl 4-methoxybenzoate (**5h**)

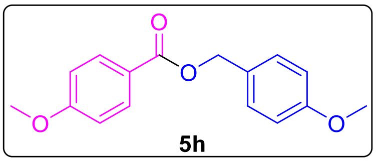

Colourless Liquid; R_f = 0.60 (Ethyl acetate/*n*-Hexane, 3:7); 72% yield; ^{1}H NMR (500 MHz, $CDCl_3$) δ: 8.03–7.99 (m, 2H), 7.40–7.36 (m, 2H), 6.93–6.90 (m, 2H), 6.90–6.88 (m, 2H), 5.27 (s, 2H), 3.85 (s, 3H), 3.82 (s, 3H); ^{13}C NMR (101 MHz, $CDCl_3$) δ: 166.3, 163.4, 159.7, 131.8, 130.1, 128.5, 122.8, 114.0, 113.6, 66.3, 55.5, 55.4; HRMS (ESI): *m/z* calcd for $C_{16}H_{17}O_4$: 273.1121; found: 273.1118 $[M+H]^+$.

4-Nitrobenzyl 4-methoxybenzoate (**5i**)[21]

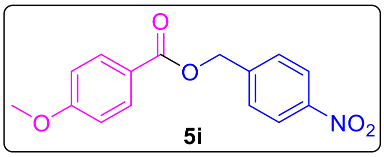

White solid; R_f = 0.53 (Ethyl acetate/*n*-Hexane, 3:7); 70% yield; mp: 136–138 °C (lit: 132-134 °C); ^{1}H NMR (400 MHz, $CDCl_3$) *δ*: 8.25 (d, *J* = 8.3 Hz, 2H), 8.04 (d, *J* = 8.5 Hz, 2H), 7.60 (d, *J* = 8.1 Hz, 2H), 6.95 (d, *J* = 8.5 Hz, 2H), 5.43 (s, 2H), 3.88 (s, 3H); ^{13}C NMR (101 MHz, $CDCl_3$) *δ*: 165.9, 163.9, 147.8, 143.8, 131.9, 128.3, 123.9, 121.9, 113.9, 65.0, 55.6; HRMS (ESI): *m/z* calcd for $C_{15}H_{14}NO_5$: 288.0866; found: 288.0865 $[M+H]^+$.

N-Phenylbenzamide (**7a**)[2, 6]

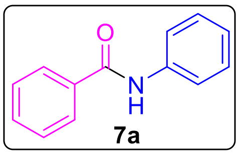

White solid; R_f = 0.46 (Ethyl acetate/*n*-Hexane, 3:7); 88% yield; mp: 162–164 °C (lit: 162–163 °C); ^{1}H NMR (300 MHz, $CDCl_3$) *δ*: 7.87 (d, *J* = 7.3 Hz, 2H), 7.83 (bs, 1H), 7.64 (d, *J* = 7.9 Hz, 2H), 7.55 (t, *J* = 7.3 Hz, 1H), 7.39 (t, *J* = 7.6 Hz, 2H), 7.37 (t, *J* = 7.7 Hz, 2H), 7.16 (t, *J* = 7.4 Hz, 1H); ^{13}C NMR (75 MHz, $CDCl_3$+DMSO–d_6) *δ*: 164.9, 137.9, 134.1, 130.1, 127.3, 127.0, 126.5, 122.5, 119.4; HRMS (ESI): *m/z* calcd for $C_{13}H_{12}NO$: 198.0913; found: 198.0915 $[M+H]^+$.

N-(4-Methoxyphenyl)benzamide (**7b**)[6, 22]

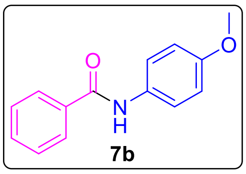

White solid; R_f = 0.51 (Ethyl acetate/*n*-Hexane, 3:7); 85% yield; mp: 150–152 °C (lit: 152–155 °C); ^{1}H NMR (500 MHz, $CDCl_3$) *δ*: 7.86 (d, *J* = 7.3 Hz, 2H), 7.79 (s, 1H), 7.55 (s, 1H), 7.53 (dd, *J* = 5.0, 2.3 Hz, 2H), 7.47 (t, *J* = 7.5 Hz, 2H), 6.93–6.88 (m, 2H), 3.81 (s, 3H); ^{13}C NMR (75 MHz, $CDCl_3$+DMSO–d_6) *δ*: 164.3, 154.5, 134.0, 130.9, 129.8, 126.8, 126.3, 120.9, 112.3, 53.9; HRMS (ESI): *m/z* calcd for $C_{14}H_{14}NO_2$: 228.1019; found: 228.1024 $[M+H]^+$.

N-(4-Chlorophenyl)benzamide (**7c**)[22]

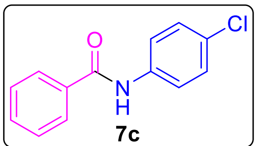

7c

White solid; R_f = 0.46 (Ethyl acetate/*n*-Hexane, 3:7); 84% yield; mp: 204–207 °C (lit: 202–204 °C); ^{1}H NMR (300 MHz, $CDCl_3$+DMSO–d_6) δ: 10.23 (s, 1H), 7.96 (dd, *J* = 1.7, 8.5 Hz, 2H), 7.82 (d, *J* = 9.0 Hz, 2H), 7.59–7.45 (m, 3H), 7.30 (d, *J* = 8.8 Hz, 2H); ^{13}C NMR (75 MHz, $CDCl_3$+DMSO–d_6) δ: 165.4, 137.6, 134.5, 131.0, 127.9, 127.8, 127.3, 121.4; HRMS (ESI): *m/z* calcd for $C_{13}H_{11}ClNO$: 232.0524; found: 232.0534 $[M+H]^+$.

N-(4-Nitrophenyl)benzamide **(7d)**[6, 22]

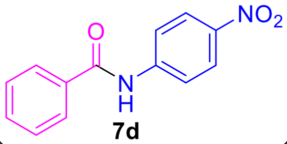

7d

Off White solid; R_f = 0.51 (Ethyl acetate/*n*-Hexane, 3:7); 80% yield; mp: 201–203 °C (lit: 200–202 °C); ^{1}H NMR (500 MHz, $CDCl_3$) δ: 10.25 (s, 1H), 8.25–8.18 (m, 2H), 8.07–8.03 (m, 2H), 8.00-7.96 (m, 2H), 7.58 (dd, *J* = 10.5, 4.2 Hz, 1H), 7.50 (t, *J* = 7.5 Hz, 2H); ^{13}C NMR (126 MHz, $CDCl_3$) δ: 166.5, 144.7, 143.6, 134.5, 132.3, 128.8, 127.7, 125.0, 119.8; HRMS (ESI): *m/z* calcd for $C_{13}H_{11}N_2O_3$: 243.0764; found: 243.0772 $[M+H]^+$.

N-(Benzo[*d*]thiazol-2-yl)benzamide (**7e**)[23]

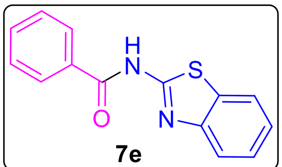

7e

White solid; R_f = 0.50 (Ethyl acetate/*n*-Hexane, 3:7); 80% yield; mp: 69–70 °C (lit: 66–68 °C); ^{1}H NMR (400 MHz, $CDCl_3$) δ: 11.34 (bs, 1H), 8.00 (dd, *J* = 1.0, 8.1 Hz, 2H), 7.89–7.1 (m, 1H), 7.57 (t, *J* = 7.5 Hz, 1H), 7.44 (t, *J* = 7.5 Hz, 2H), 7.39–7.27 (m, 3H); ^{13}C NMR (125 MHz, $CDCl_3$) δ: 166.0, 159.7, 147.7, 133.0, 132.0, 131.8, 128.9, 127.9, 126.0, 123.9, 121.3, 120.6; HRMS (ESI): *m/z* calcd for $C_{14}H_{11}N_2OS$: 255.0587; found: 255.0594 $[M+H]^+$.

(4-Nitrophenyl)(pyrrolidin-1-yl)methanone (**7f**)[24]

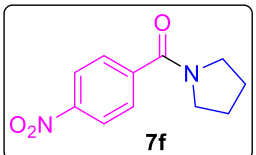

7f

Off White solid; R_f = 0.15 (Ethyl acetate/*n*-Hexane, 3:7); 78% yield; mp: 92–94 °C (lit: 94-96 °C); ^{1}H NMR (400 MHz, $CDCl_3$) δ: 8.22 (d, J = 7.0 Hz, 2H), 7.64 (d, J = 7.8 Hz, 2H), 3.48 (d, J = 110.6 Hz, 4H), 1.92 (d, J = 27.5 Hz, 4H); ^{13}C NMR (101 MHz, $CDCl_3$) δ: 167.4, 148.4, 143.1, 128.1, 123.7, 49.4, 46.4, 26.4, 24.3; HRMS (ESI): *m/z* calcd for $C_{11}H_{13}N_2O_3$: 221.0920; found: 221.0952 $[M+H]^+$.

4-Methoxy-*N*-phenylbenzamide (**7g**)[25]

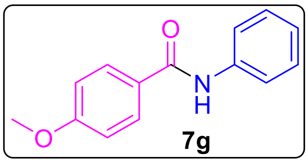

White solid; R_f = 0.58 (Ethyl acetate/*n*-Hexane, 3:7); 87% yield; mp: 164–166 °C (lit: 161-162 °C); ^{1}H NMR (400 MHz, $CDCl_3$) δ: 7.86–7.82 (m, 2H), 7.80 (s, 1H), 7.63 (d, J = 7.6 Hz, 2H), 7.37 (d, J = 7.6 Hz, 2H), 7.14 (t, J = 7.4 Hz, 1H), 6.99–6.93 (m, 2H), 3.87 (s, 3H); ^{13}C NMR (75 MHz, $CDCl_3$+DMSO–d_6) δ: 164.0, 160.7, 138.0, 128.3, 127.1, 125.9, 122.1, 119.3, 112.1, 54.0; HRMS (ESI): *m/z* calcd for $C_{14}H_{14}NO_2$: 228.1019; found: 228.1025 $[M+H]^+$.

III. References

1. Liliana, L.; Aliana, G.; Dalila, B.; Benoit, R.; Joelle, D.; Elena, B. *Bioorg. Med. Chem. Lett*. 2015, *25* 3975–3979.
2. Li, J.; Xu, F.; Zhang, Y.; Shen, Q. *J. Org. Chem*. **2009**, *74*, 2575-2577.
3. Thale, P. B.; Borase, P. N.; Shankarling, G. S. *RSC Advances*. **2016**, 6, 52724 – 52728.
4. Howard, E. L., Guzzardi, N.; Tsanova, V. G.; Stika, A.; Patel, B. *Eur. J. Org. Chem*. **2018**, 6, 794–797.
5. Wang, N.; Zou, X.; Ma, J.; Li, F. *Chem. Comm*. **2014**, *50*, 8303–8305.
6. Yuan, Y-C.; Kamaraj, R.; Bruneau, C.; Labasque, T.; Roisnel, T.; Gramage-Doria, R. *Org. Lett.* **2017**, *19*, 6404-6407.
7. Wu, G.; Lv, T.; Mo, W.; Yang, X.; Gao, Y.; Chen, H.; *Tetrahedron. Lett*. **2017**, 58, 1395–1398.
8. Martinelli, J. R.; Clark, T. P.; Watson, D. A.; Munday, R. H.; Buchwald, S. L. *Angew. Chem. Int. Ed.* **2007**, *46*, 8460-8463.
9. Cline, G. W.; Hanna, S. B. *J. Am. Chem. Soc.***1987**, *109*, 3087-3091
10. Tu, Y.; Yuan, L.; Wang, T.; Wang, C.; Ke, J.; Zhao, J. *J. Org. Chem*. **2017,** *82*, 4970–4976

11. Katritzky, A. R.; Zhang, S.; Wang, M.; Kolb, H. C.; Steel, P. J. *J. Heterocyclic Chem.* **2002,** *39*, 759 – 765
12. Achar, T. K.; Mal, P. *J. Org. Chem*. **2015**, *80*, 666–672.
13. Jiang, L.; Yu, J.; Niu, F.; Zhang, D.; Sun, X. *Heteroatom Chem*. **2017,** *28*, E21364.
14. Kumarachar, T.; Mal, P. *Adv. Synth. Cat***. 2015,** *357*, 3977–3985.
15. Nammalwar, B.; Muddala, N. P.; Watts, F. M.; Bunce, R. A.*Tetrahedron.* **2015**, *71*, 9101–9111
16. Noji, M.; Ohno, T.; Fuji, K.; Futaba, N.; Tajima, H.; Ishii, K. *J. Org. Chem*. **2003**, *68*, 9340– 9347.
17. Zhang, J.; Hou, Y.; Ma, Y.; Szostak, M. *J. Org. Chem.* **2019**, *84*, 338 – 345.
18. Gaspa, S.; Porcheddu, A.; De Luca, L. *Adv. Syn. Cat.* **2016**, *358*, 154 – 158.
19. Iranpoor, N.; Firouzabadi, H.; Khalili, D. *Org. Biomol. Chem*. **2010**, *8,* 4436-4443.
20. Lyons, E.; Reid, E. E. *J. Am. Chem. Soc*. **1917**, *39*, 1727– 1735.
21. Kim, J. H.; Park, H.; Chung, Y. K. *RSC Advances.* **2017**, *7*, 190 – 194
22. Nahakpam, L.; Chipem, F. A. S.; Chingakham, B. S.; Laitonjam, W. S. *New J. Chem.* **2015**, *39*, 2240–2247.
23. Lin, Y. M.; Yi, W. B.; Shen, W. Z.; Lu, G. P. *Org. Lett*. **2016**, *18*, 592–595.
24. Leow, D. *Org. Lett*. **2014**, *16*, 5812-5815.
25. Tambade, P. J.; Patil, Y. P.; Bhanushali, M. J.; Bhanage, B. M. *Synthesis.* **2008**, *15*, 2347–2352.

V. NMR Spectra

Copies of ^{1}H NMR and ^{13}C NMR spectra of **3a**

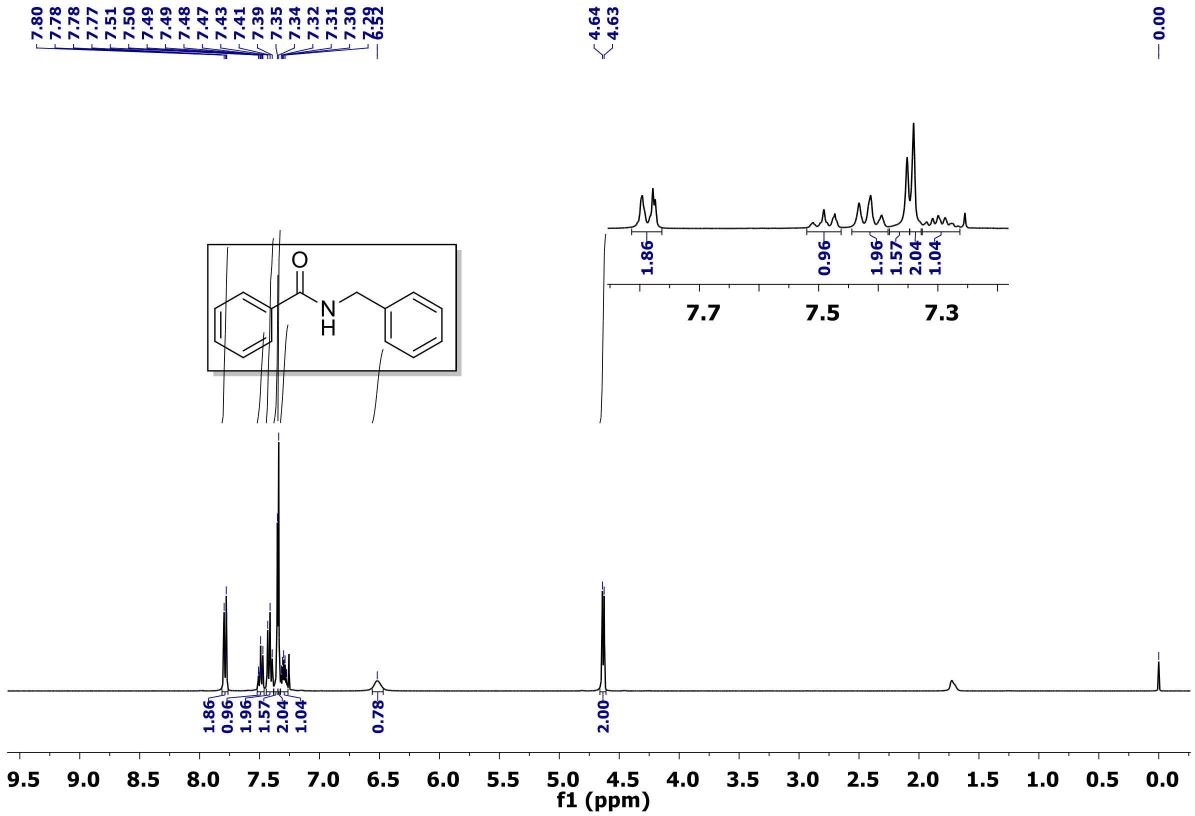

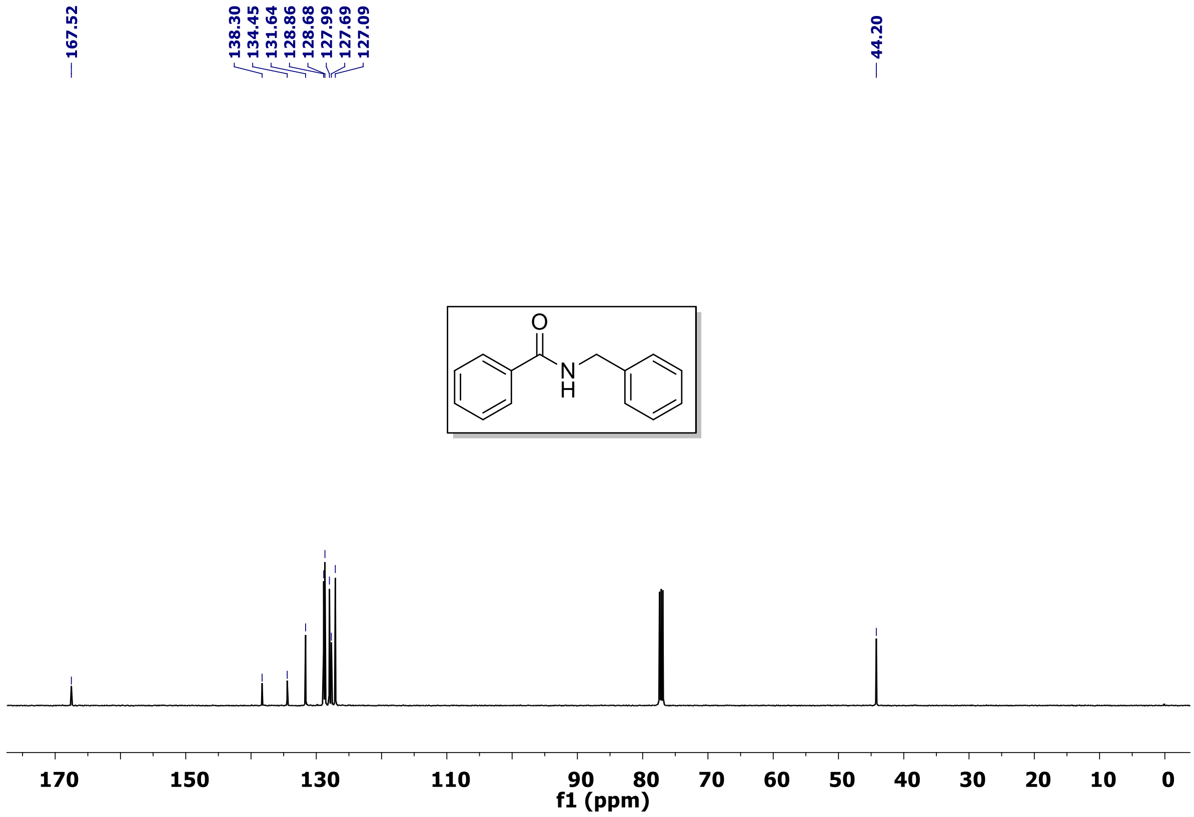

Copies of ^{1}H NMR and ^{13}C NMR spectra of **3b**

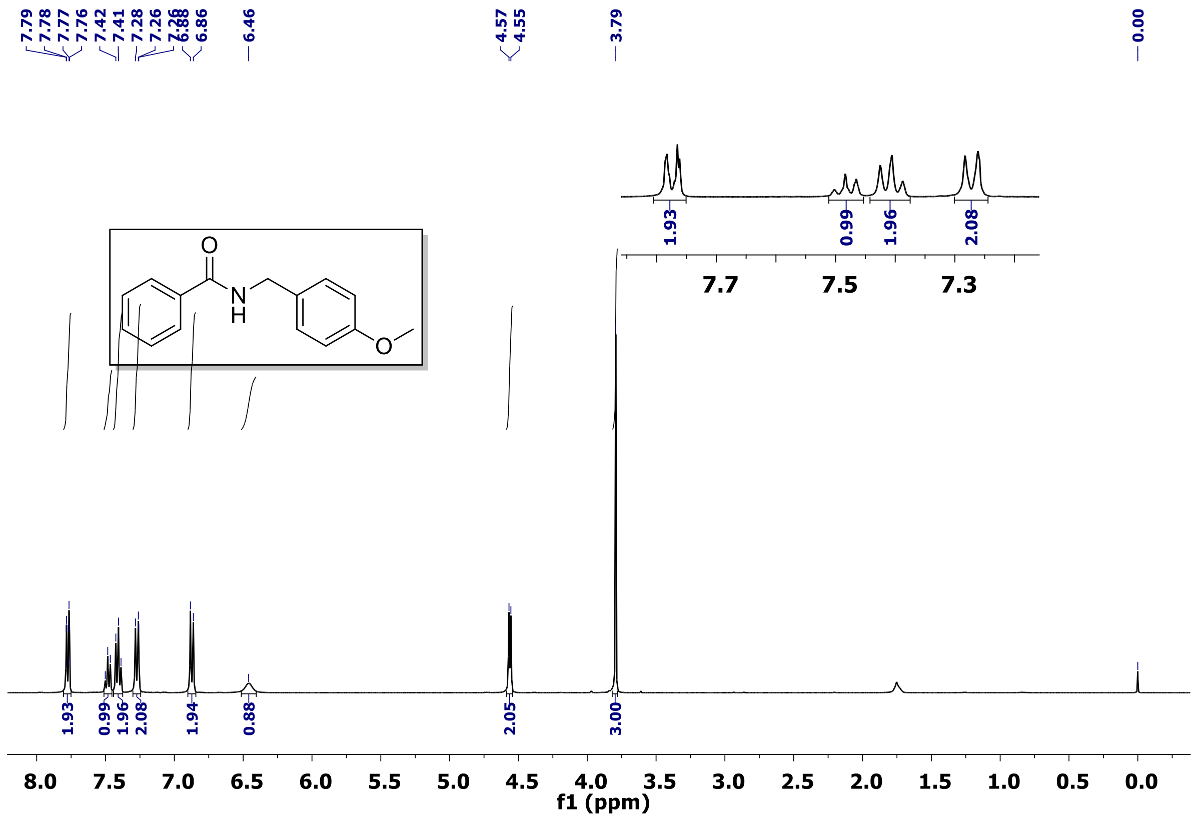

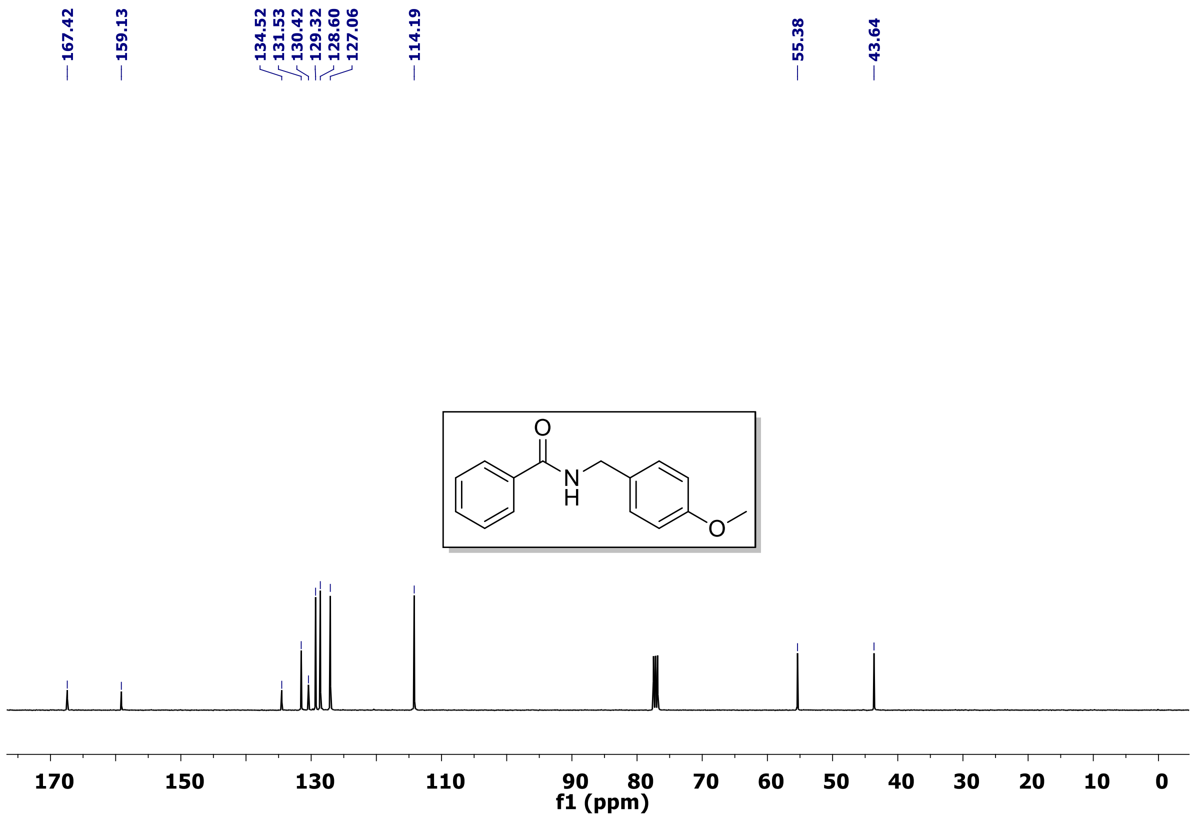

Copies of ^{1}H NMR and ^{13}C NMR spectra of **3c**

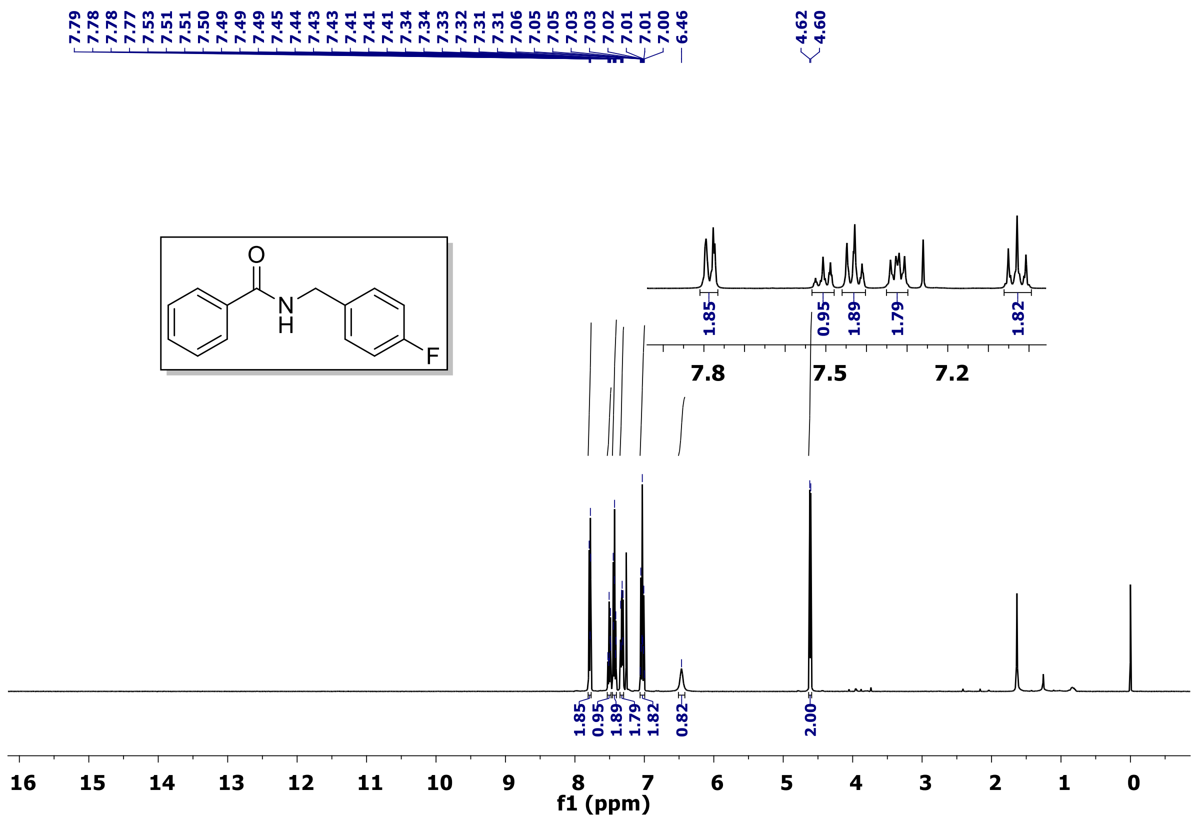

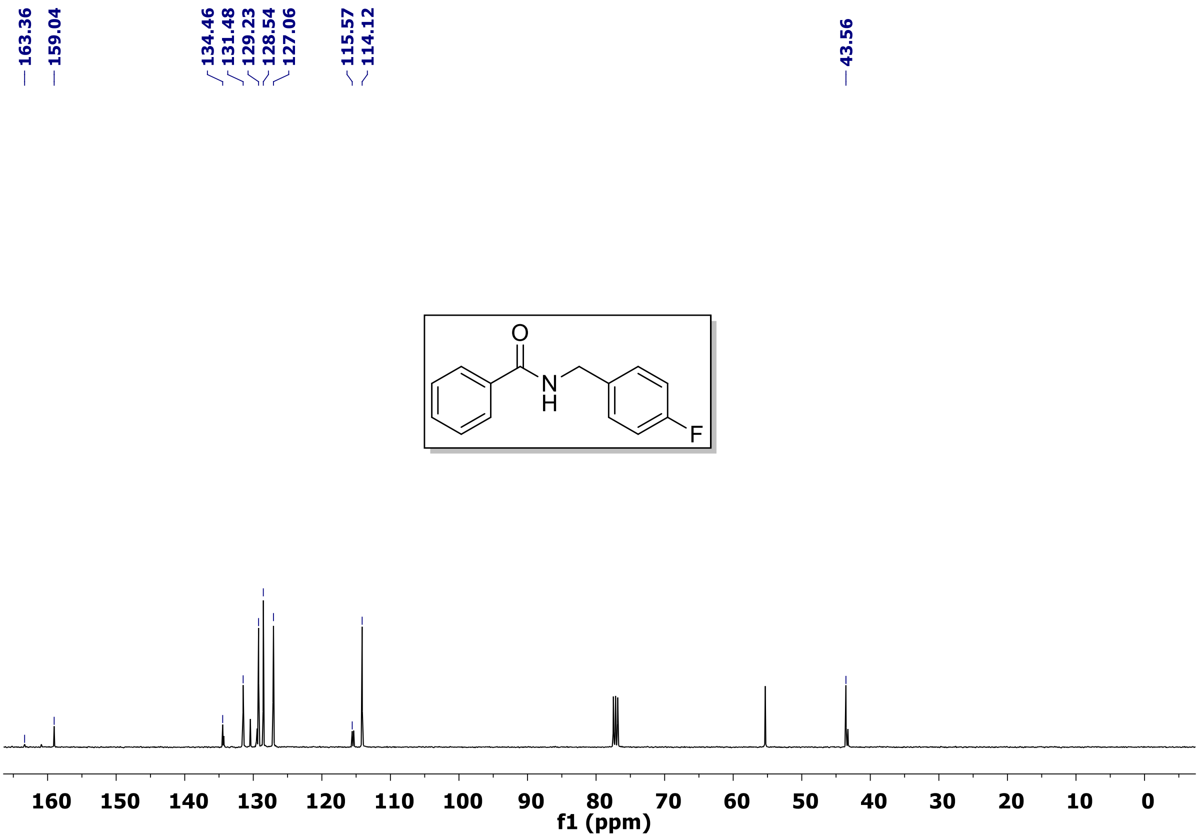

Copies of ^{1}H NMR and ^{13}C NMR spectra of **3d**

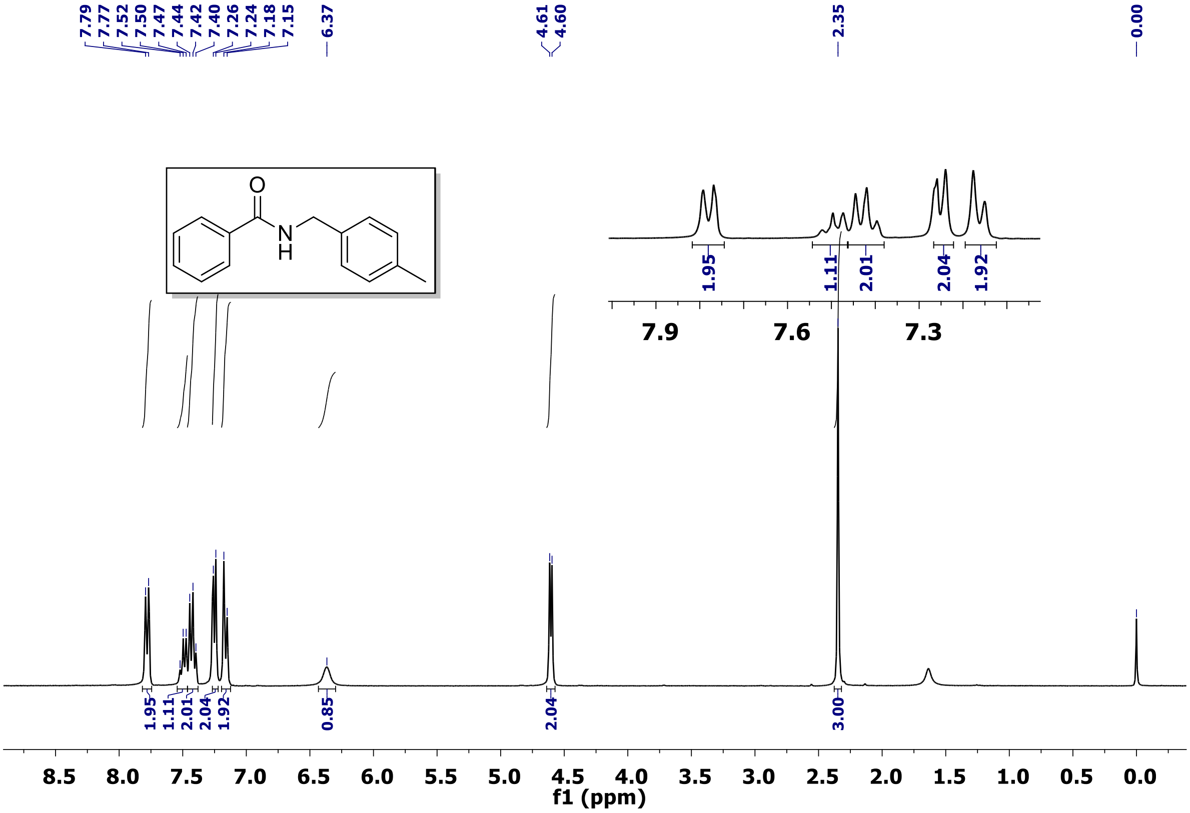

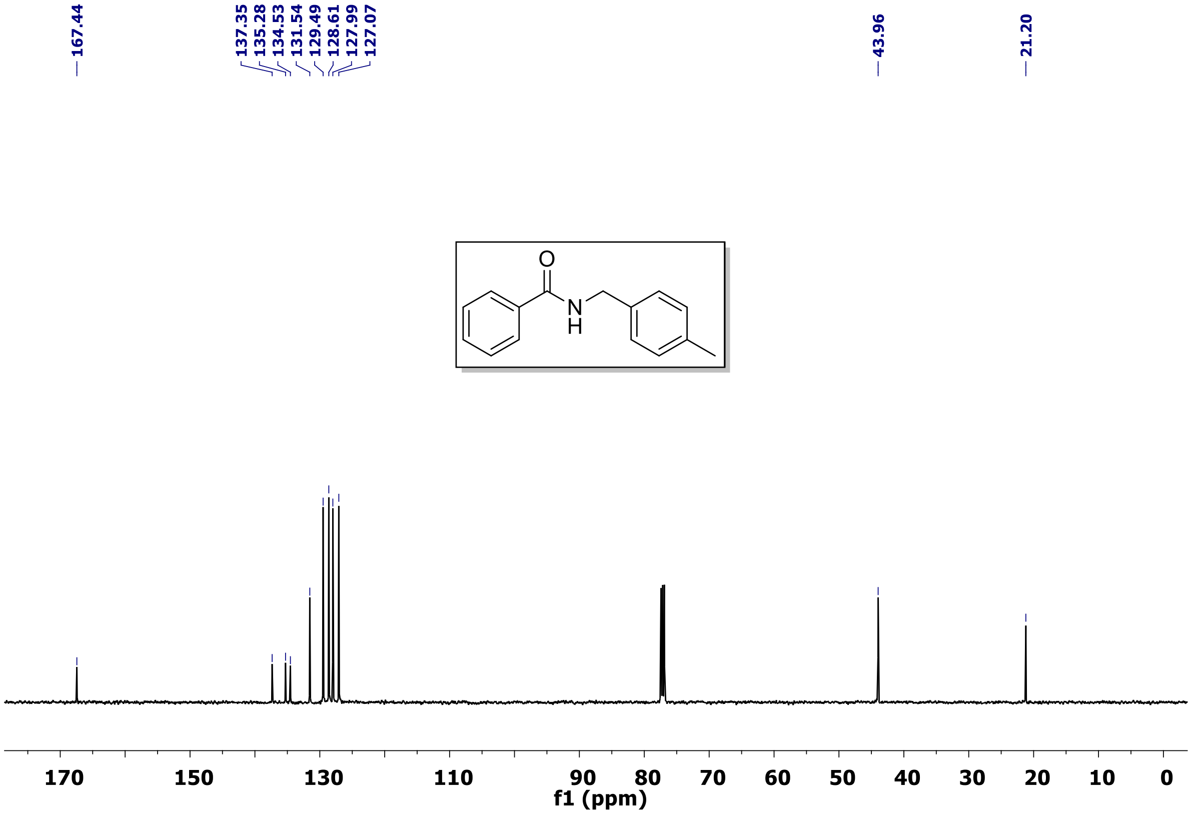

Copies of ^{1}H NMR and ^{13}C NMR spectra of **3e**

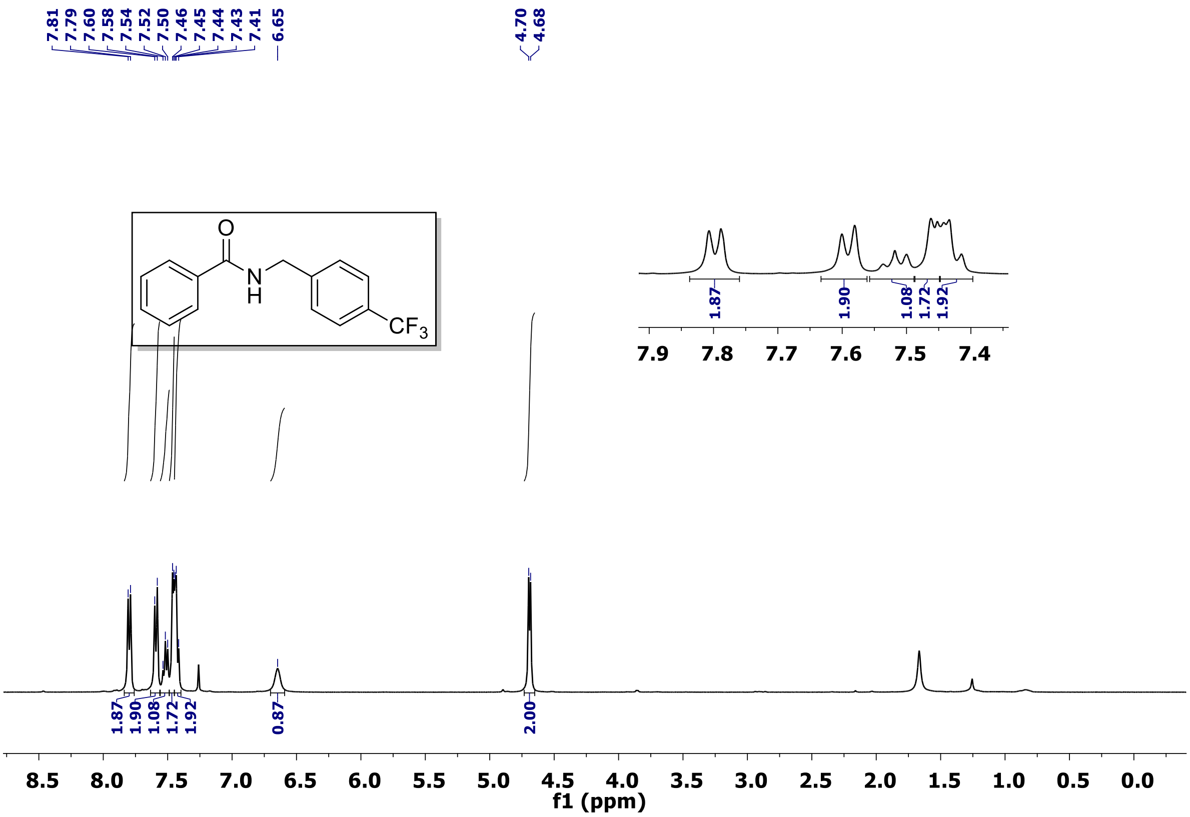

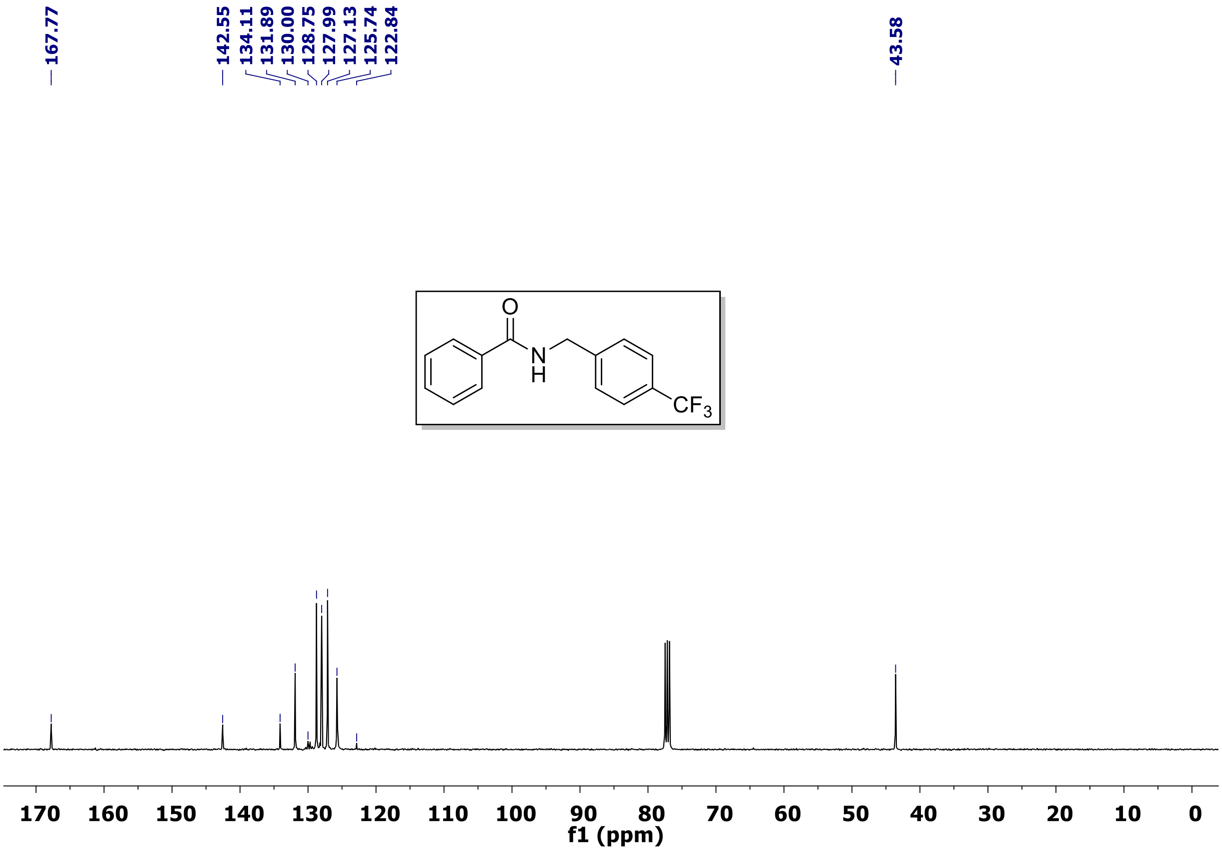

Copies of ^{1}H NMR and ^{13}C NMR spectra of **3f**

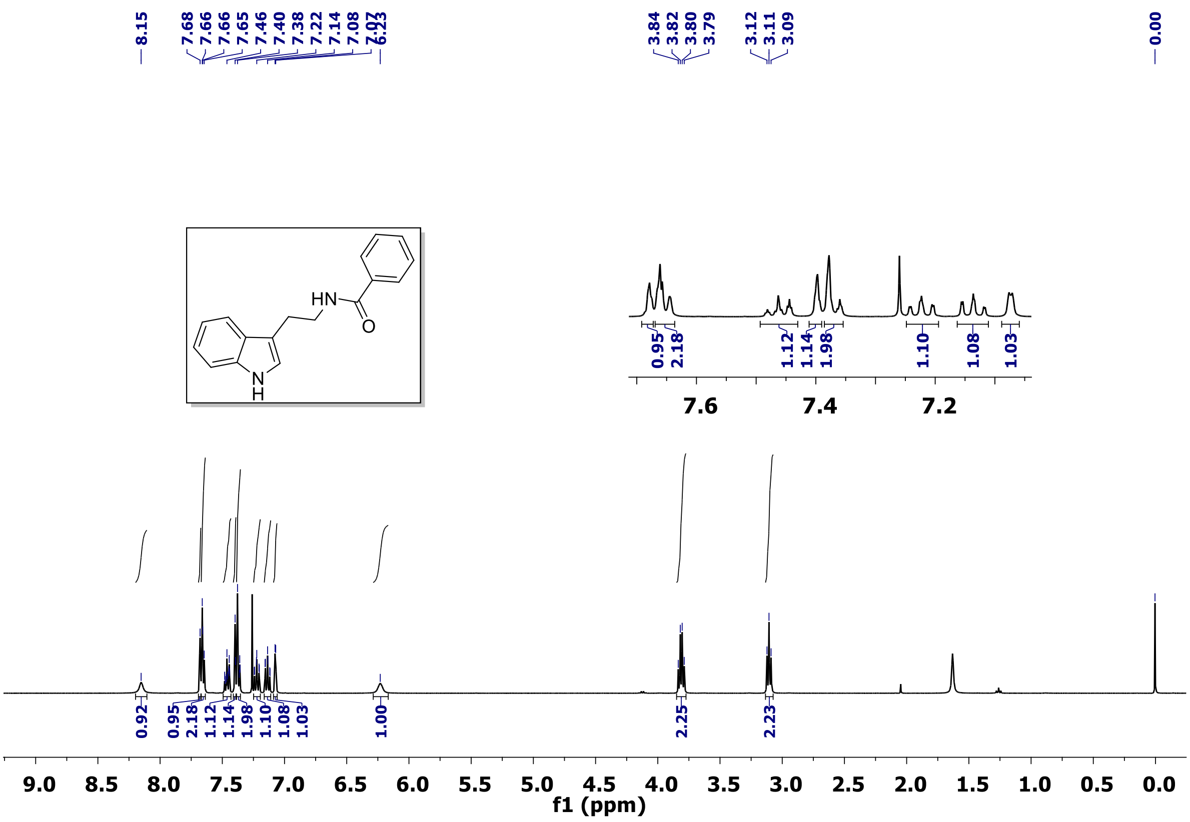

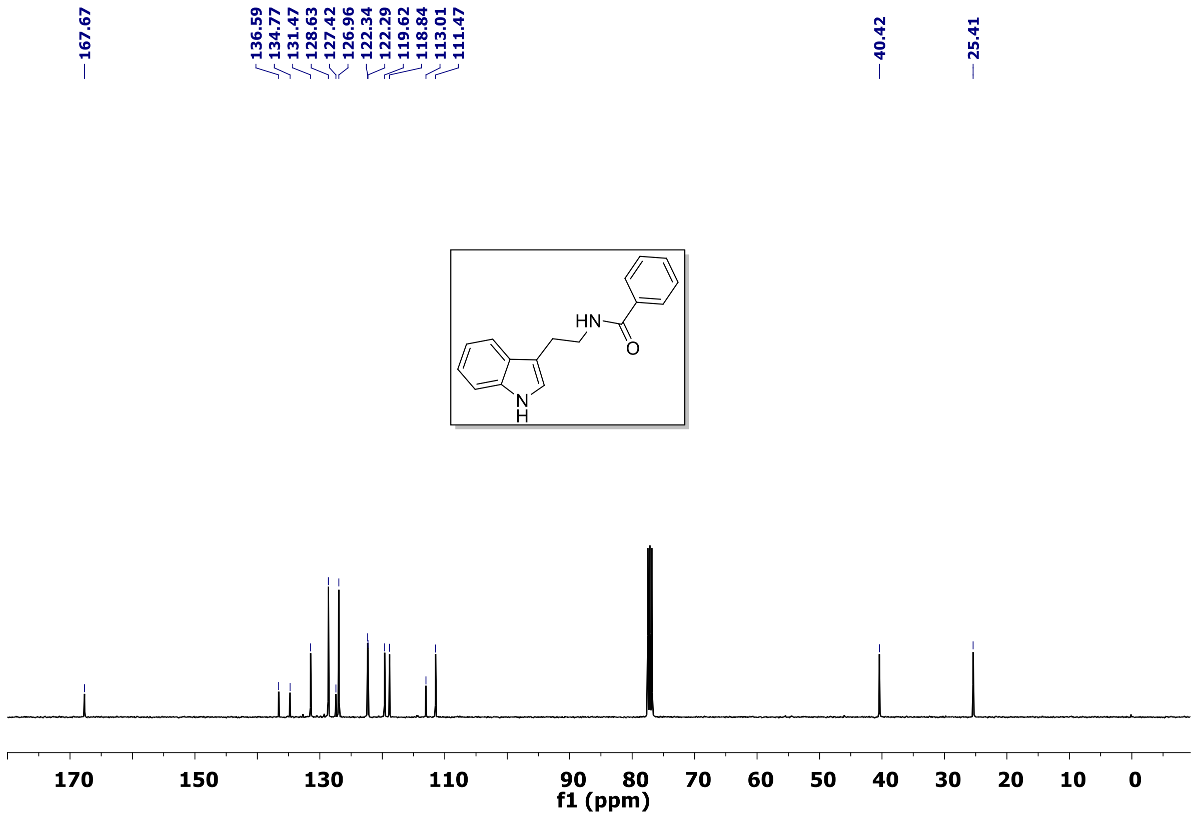

Copies of ^{1}H NMR and ^{13}C NMR spectra of **3g**

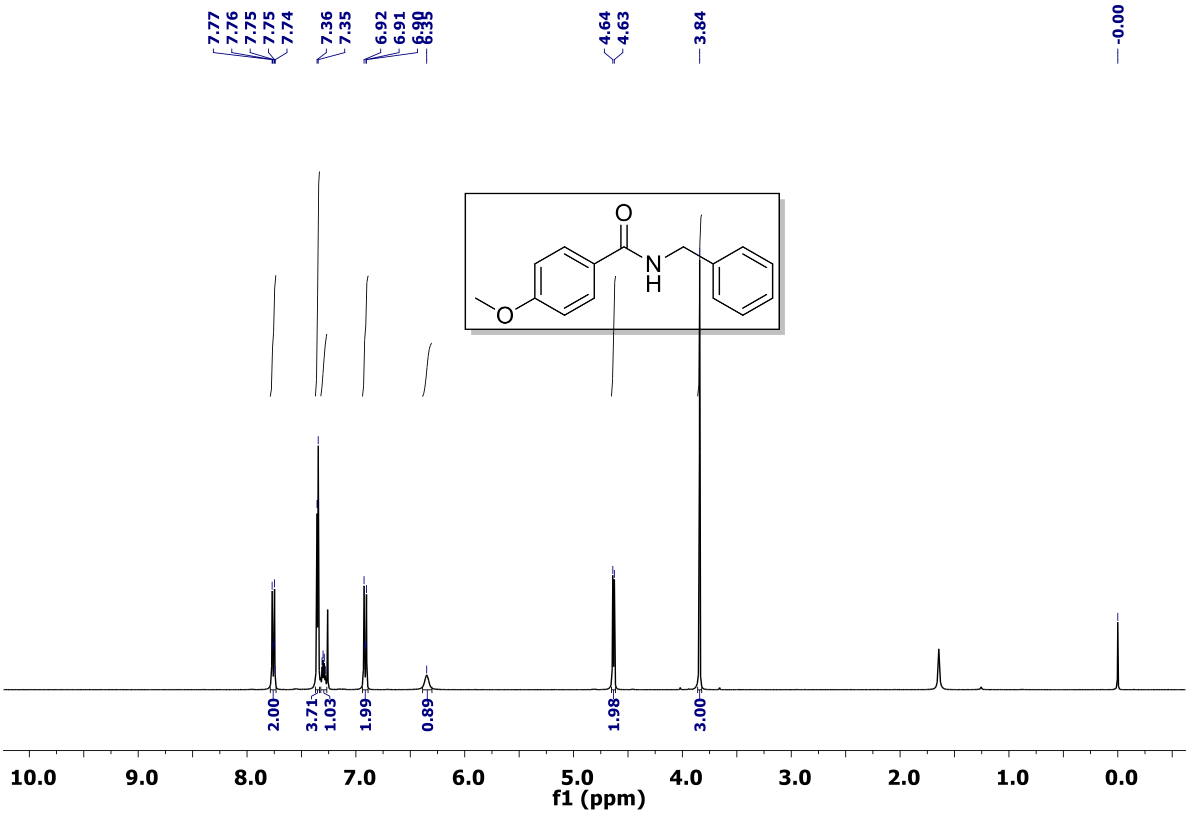

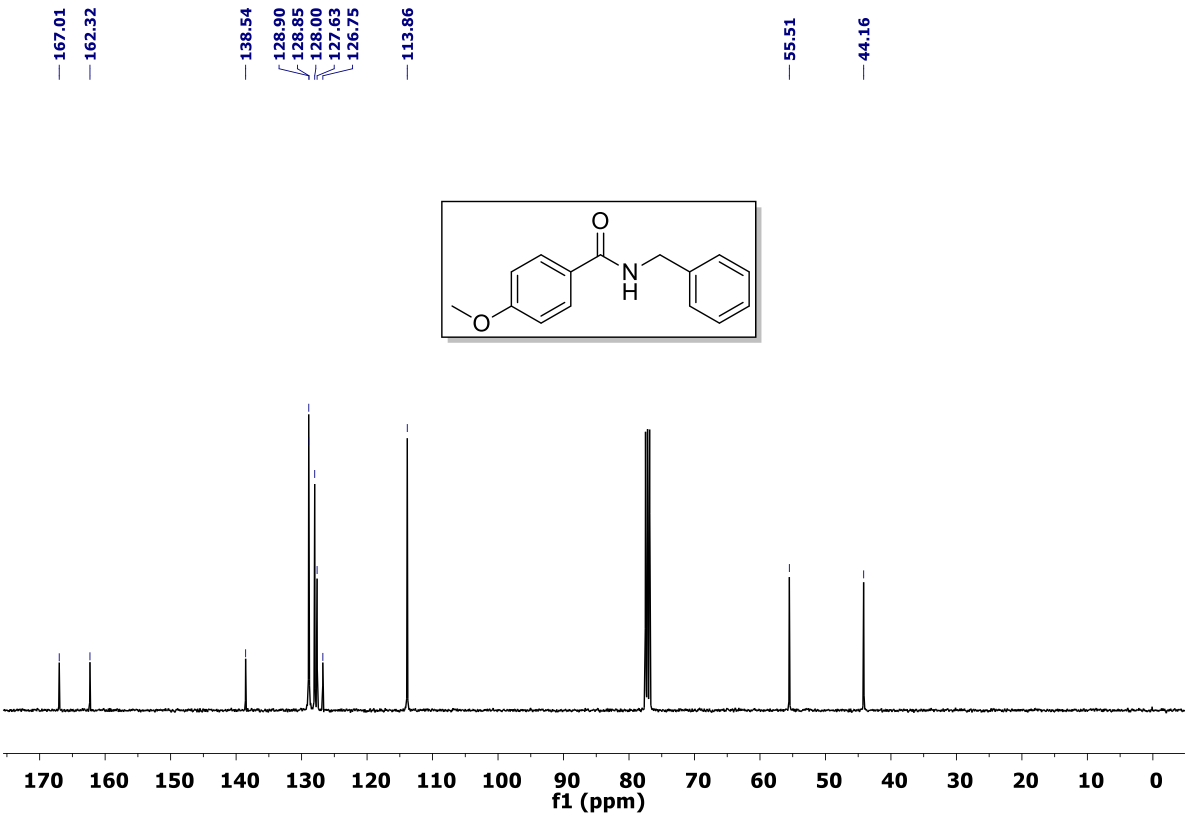

Copies of ^{1}H NMR and ^{13}C NMR spectra of **3h**

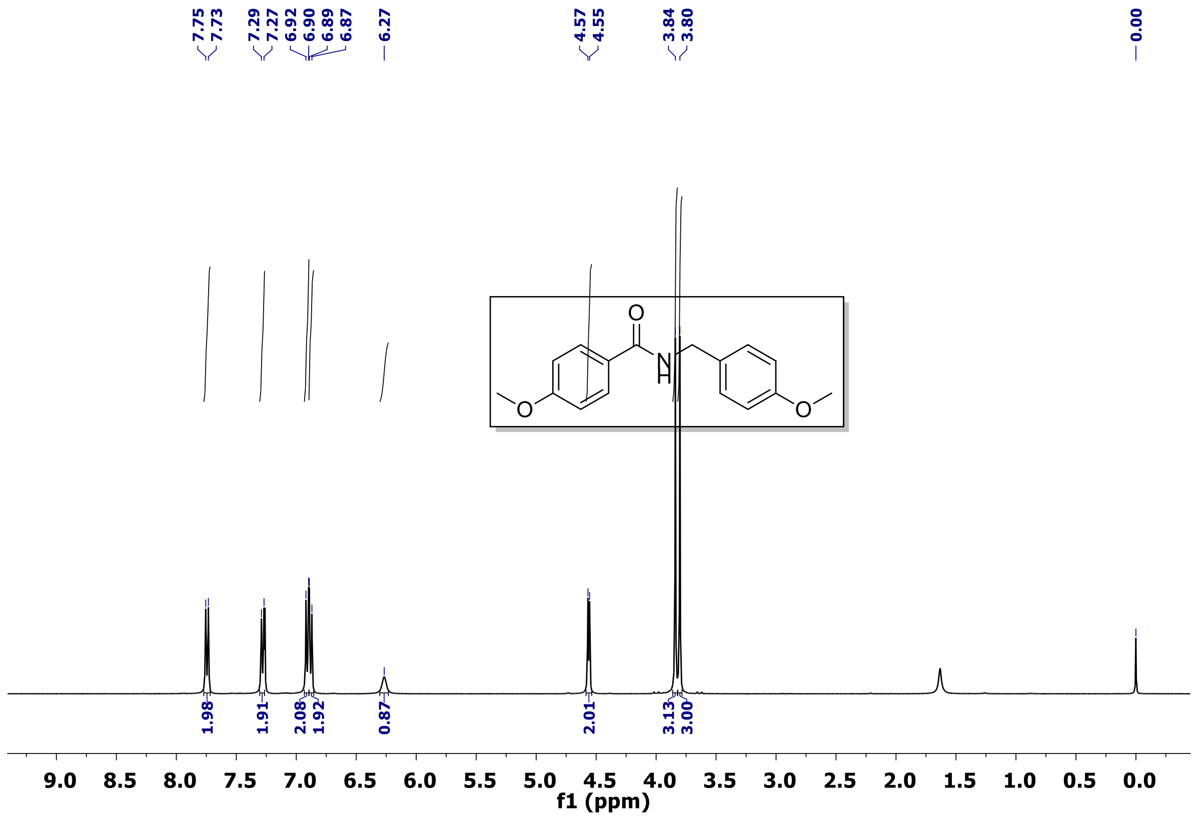

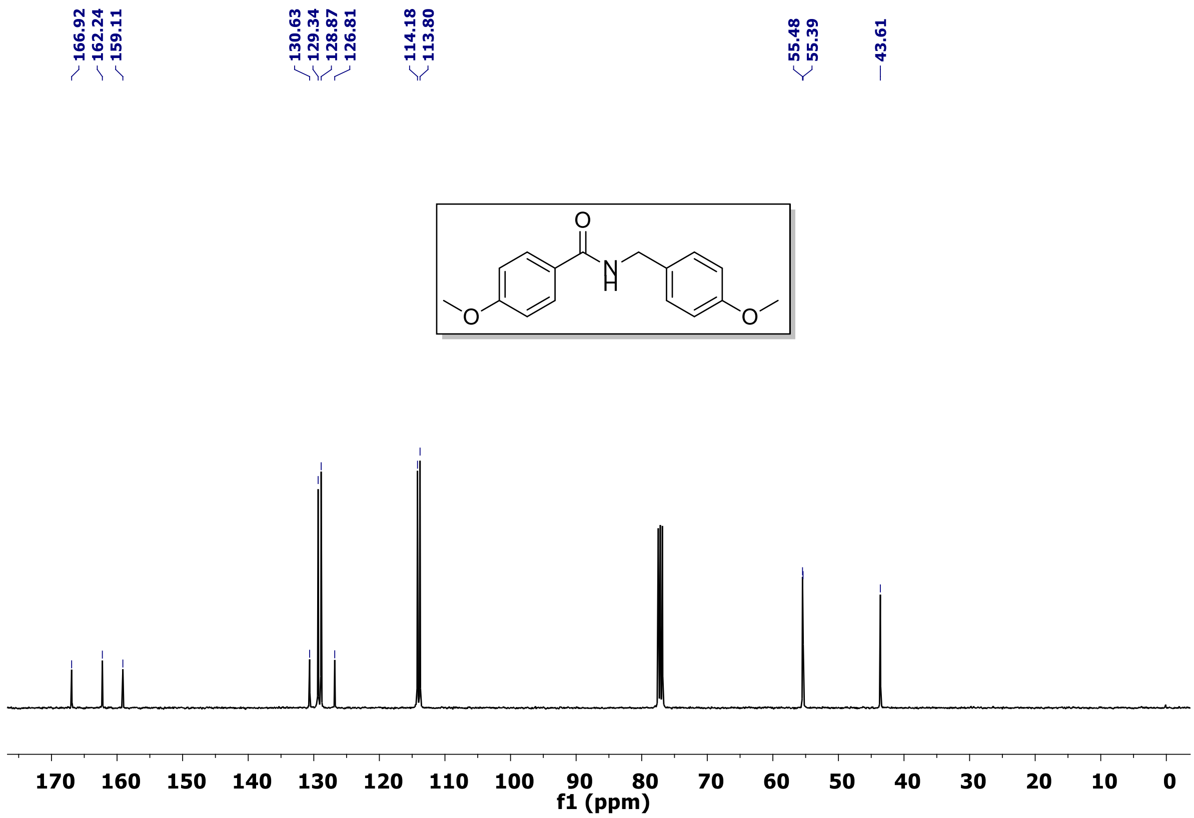

Copies of ^{1}H NMR and ^{13}C NMR spectra of **3i**

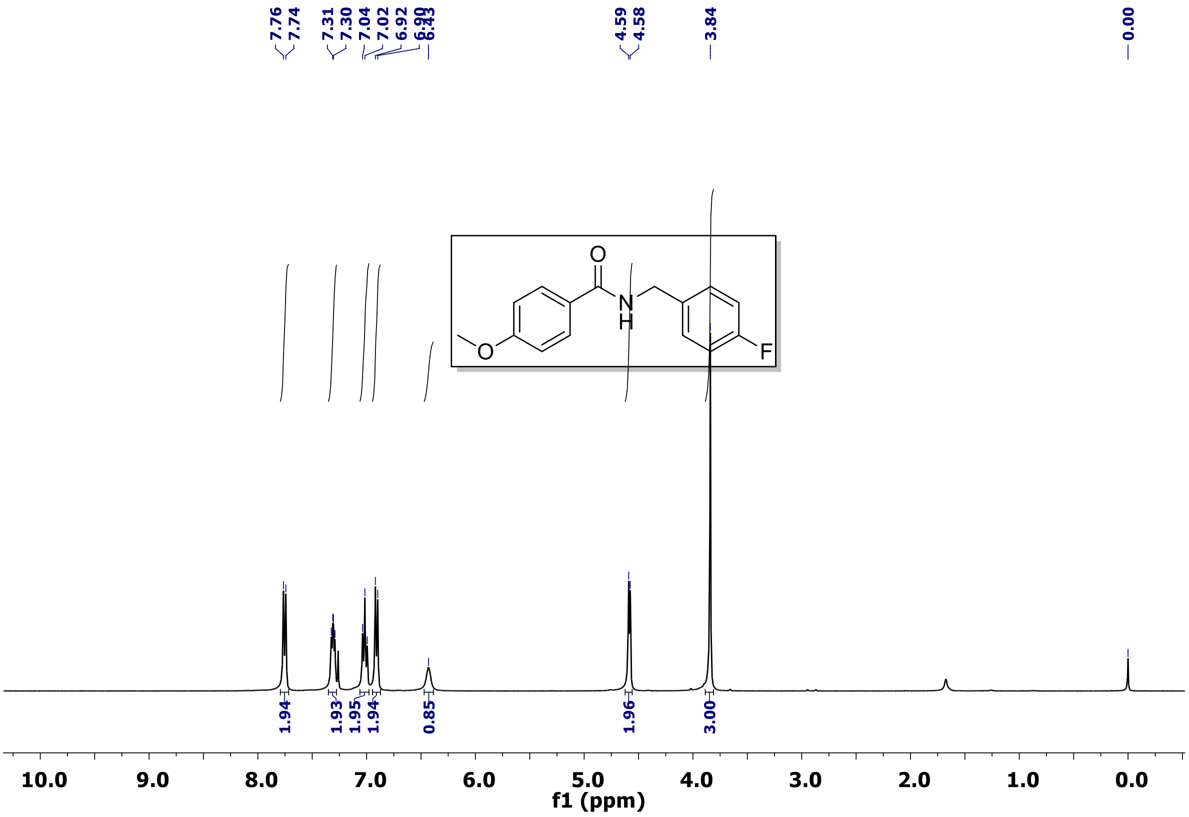

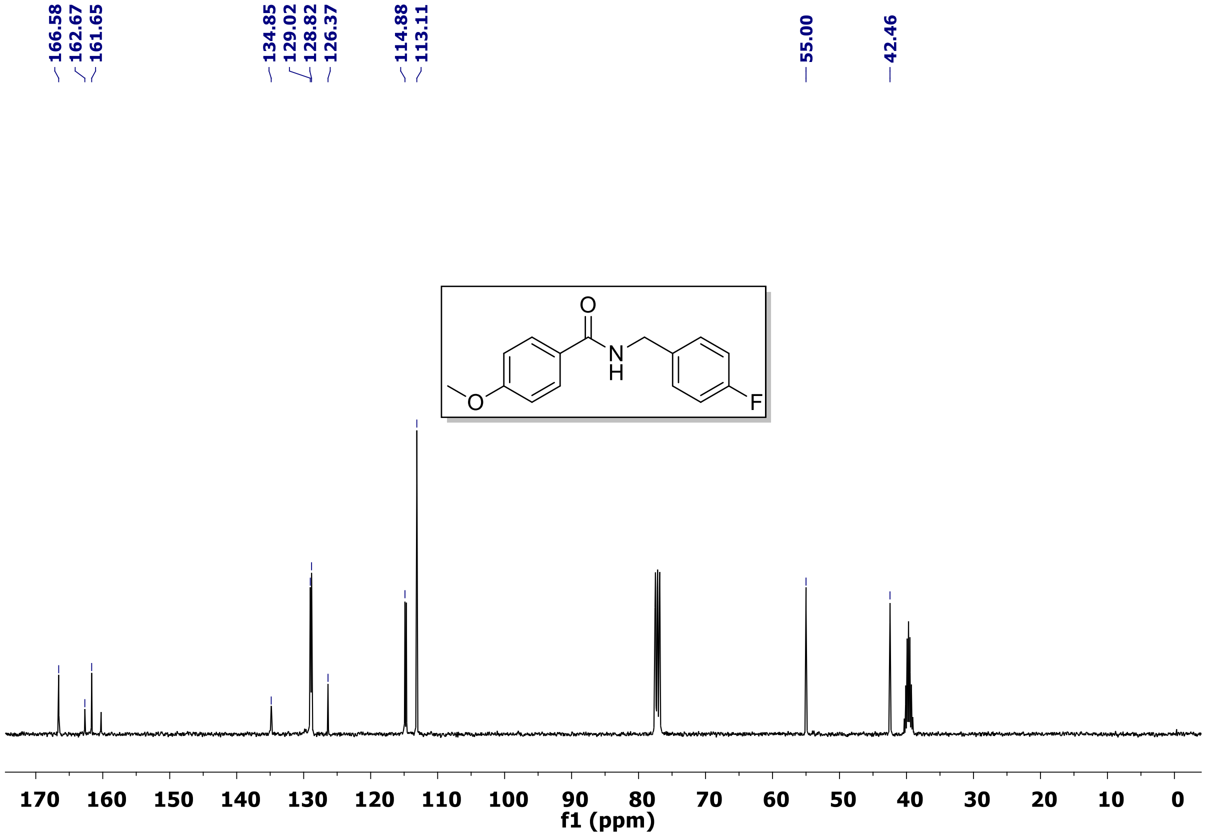

Copies of ^{1}H NMR and ^{13}C NMR spectra of **3j**

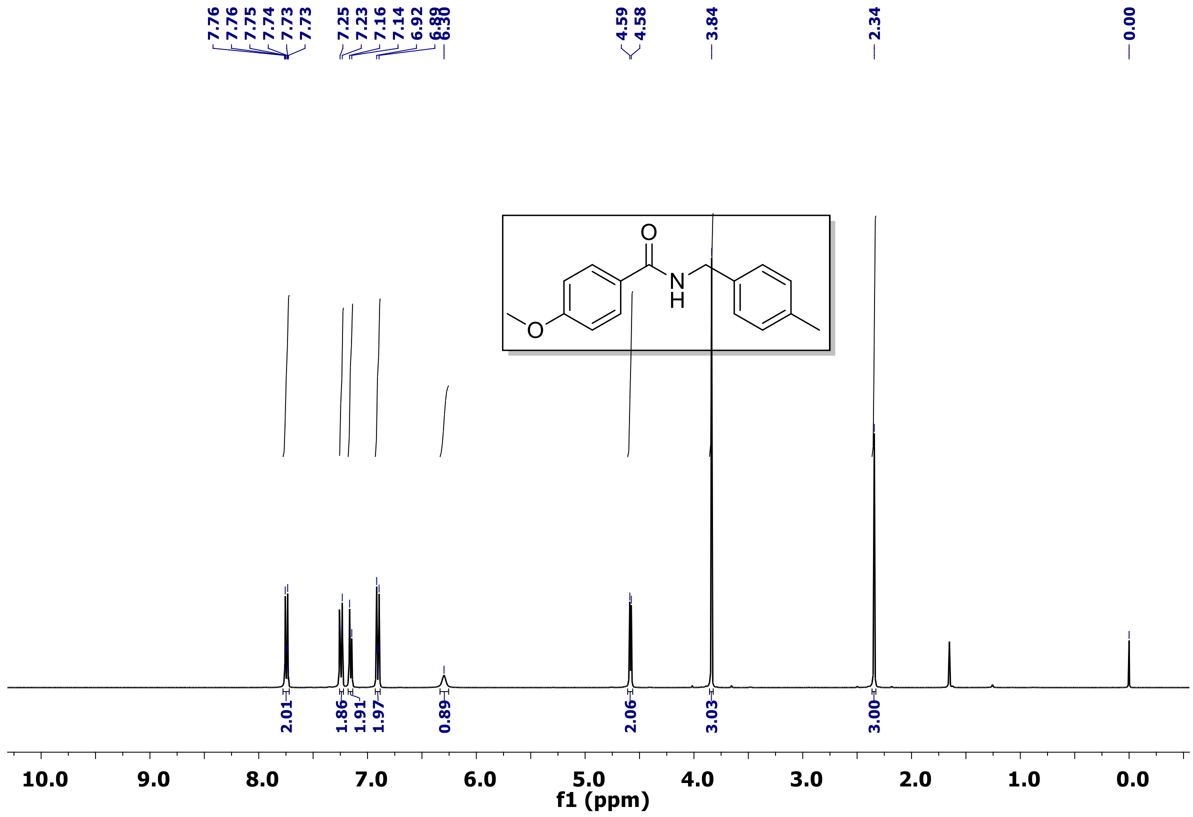

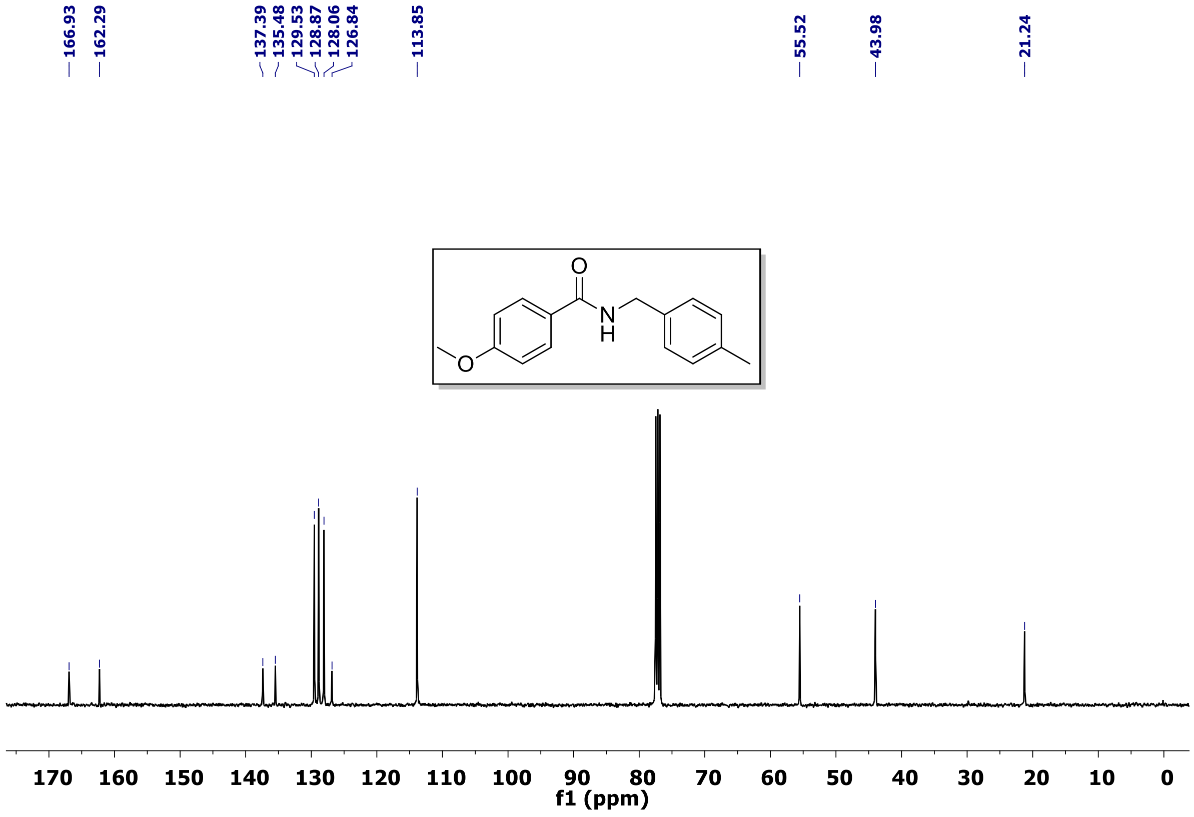

Copies of ^{1}H NMR and ^{13}C NMR spectra of **3k**

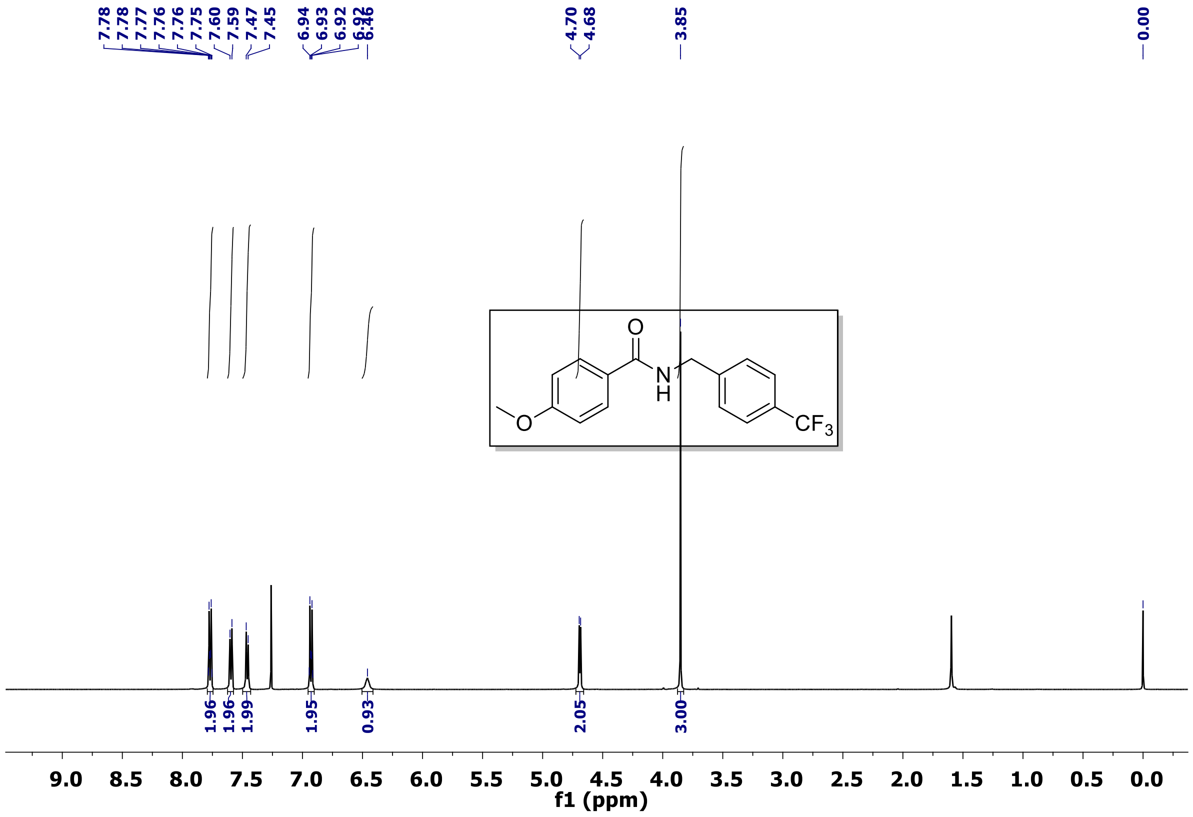

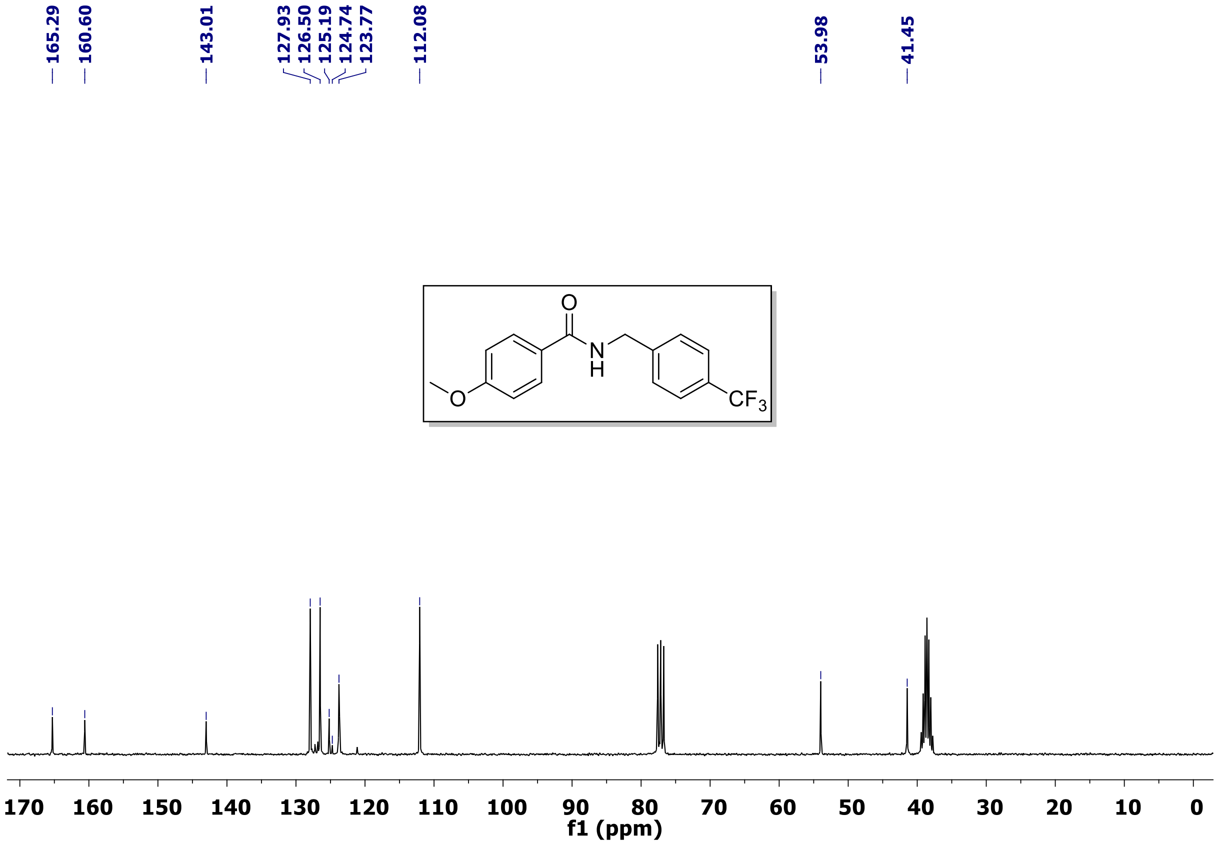

Copies of ^{1}H NMR and ^{13}C NMR spectra of **3l**

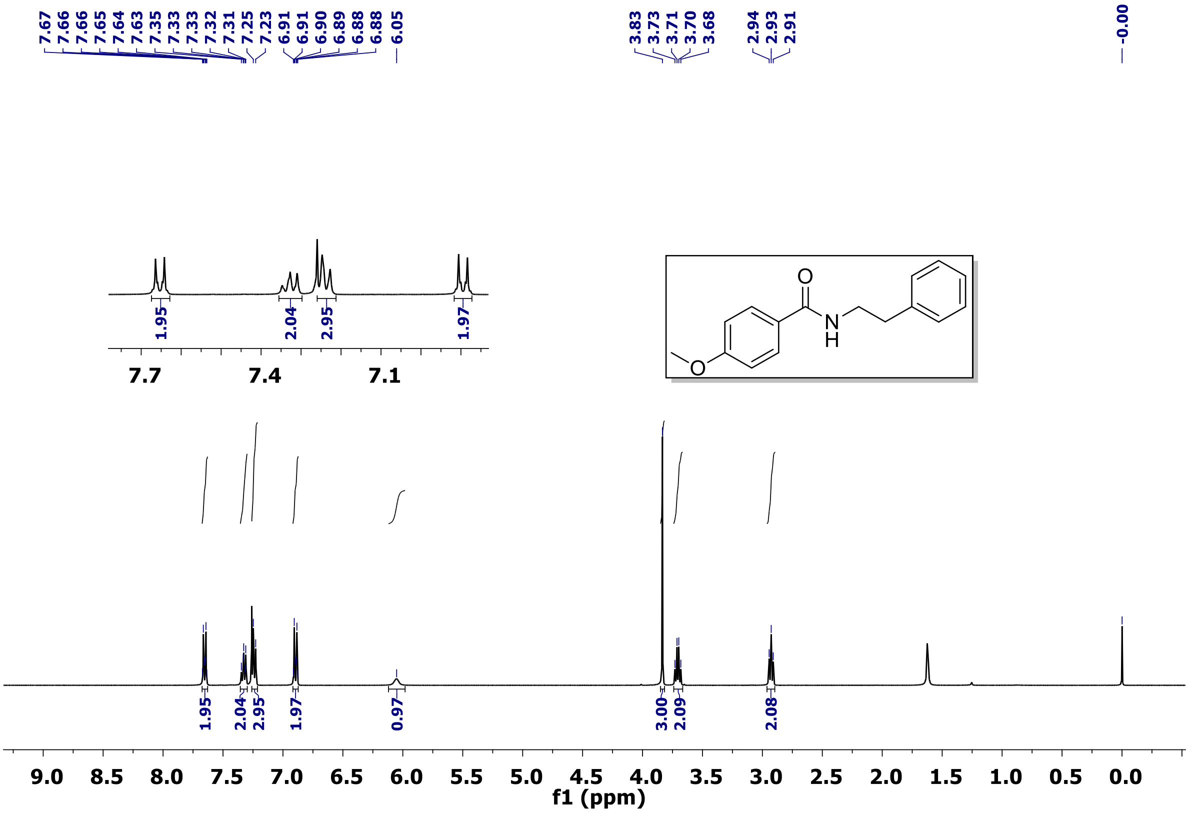

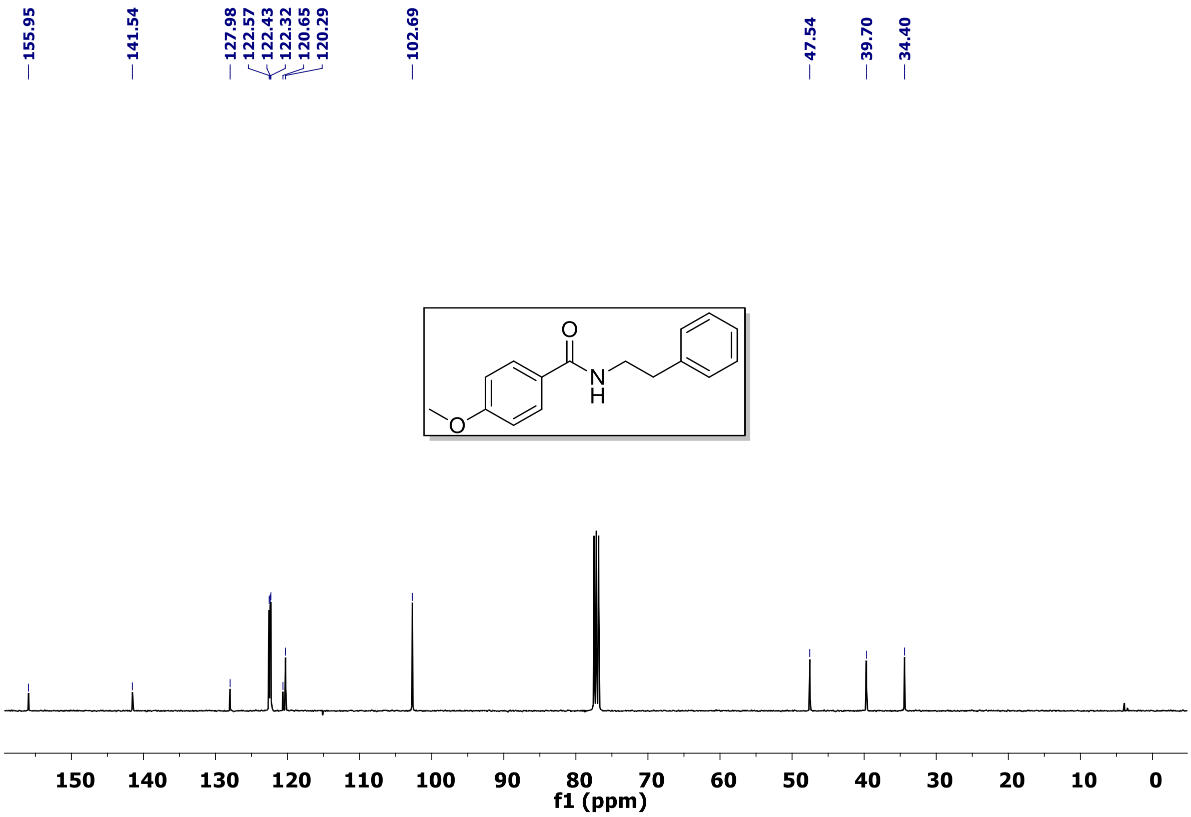

Copies of ^{1}H NMR and ^{13}C NMR spectra of **3m**

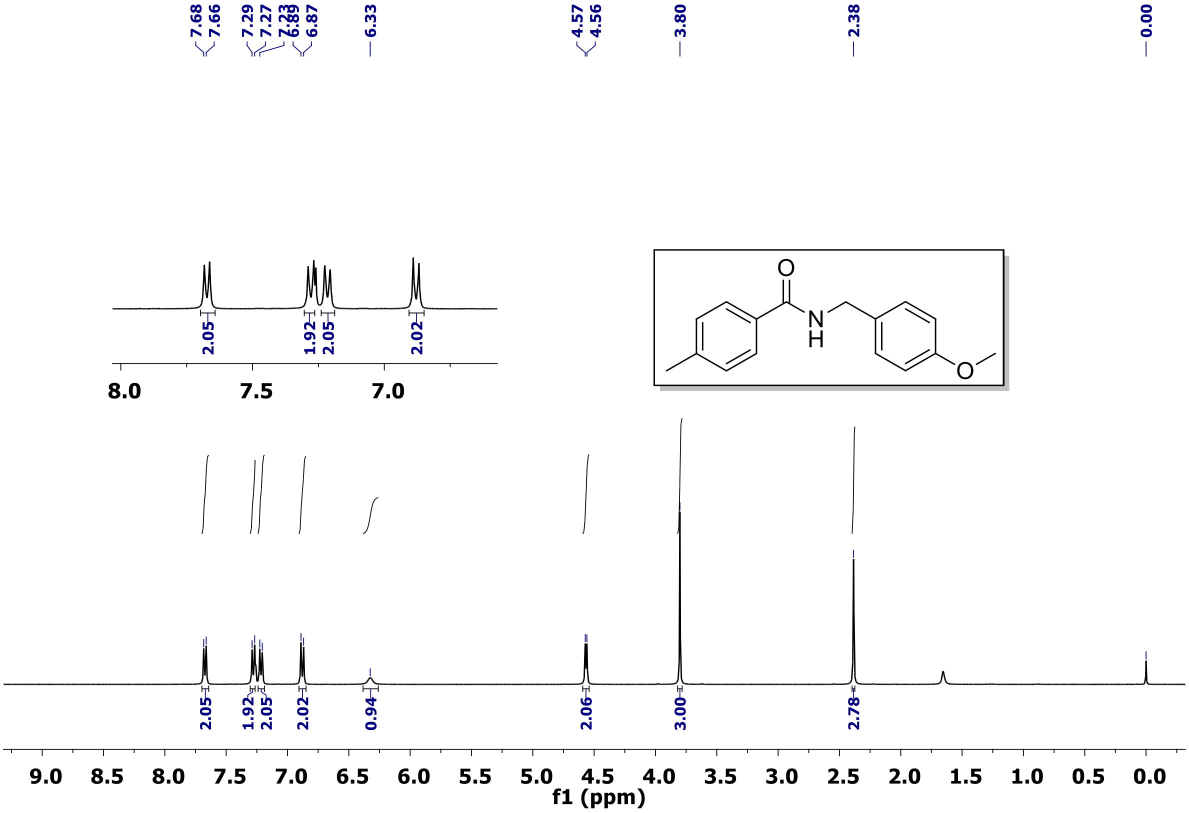

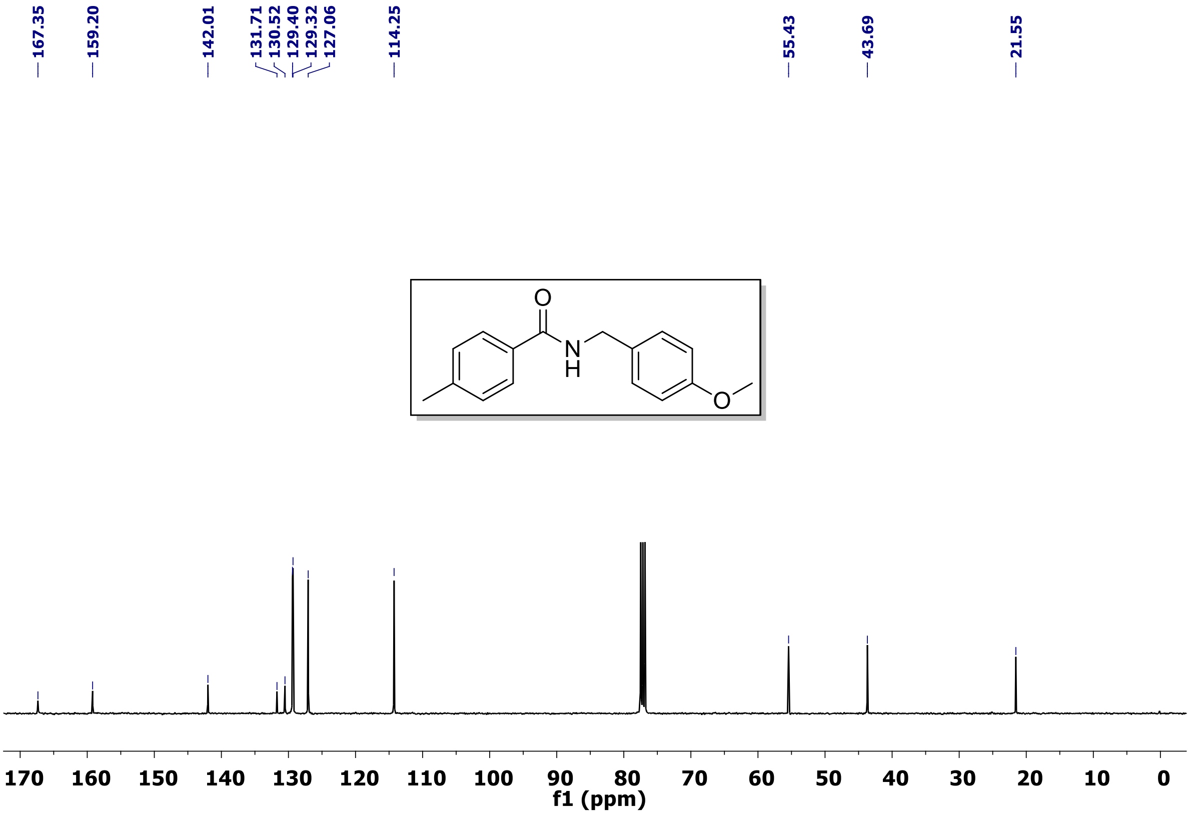

Copies of ^{1}H NMR and ^{13}C NMR spectra of **3n**

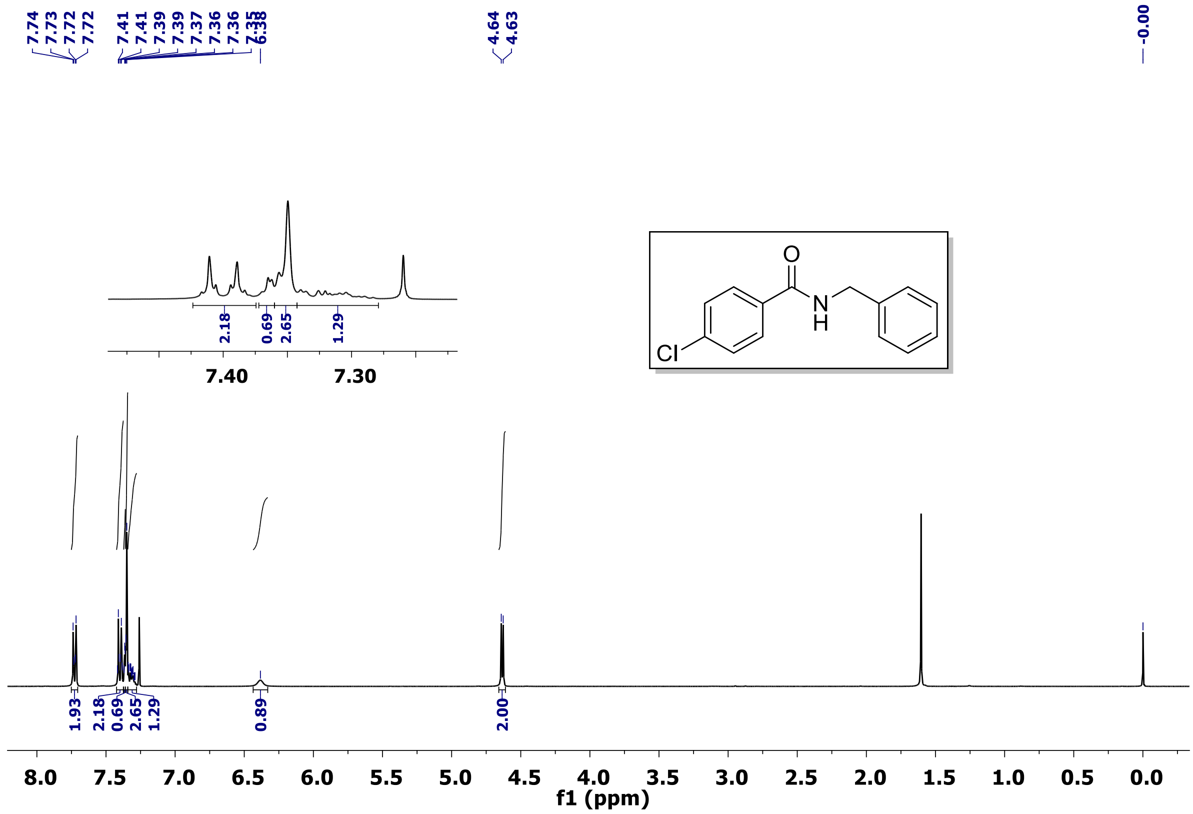

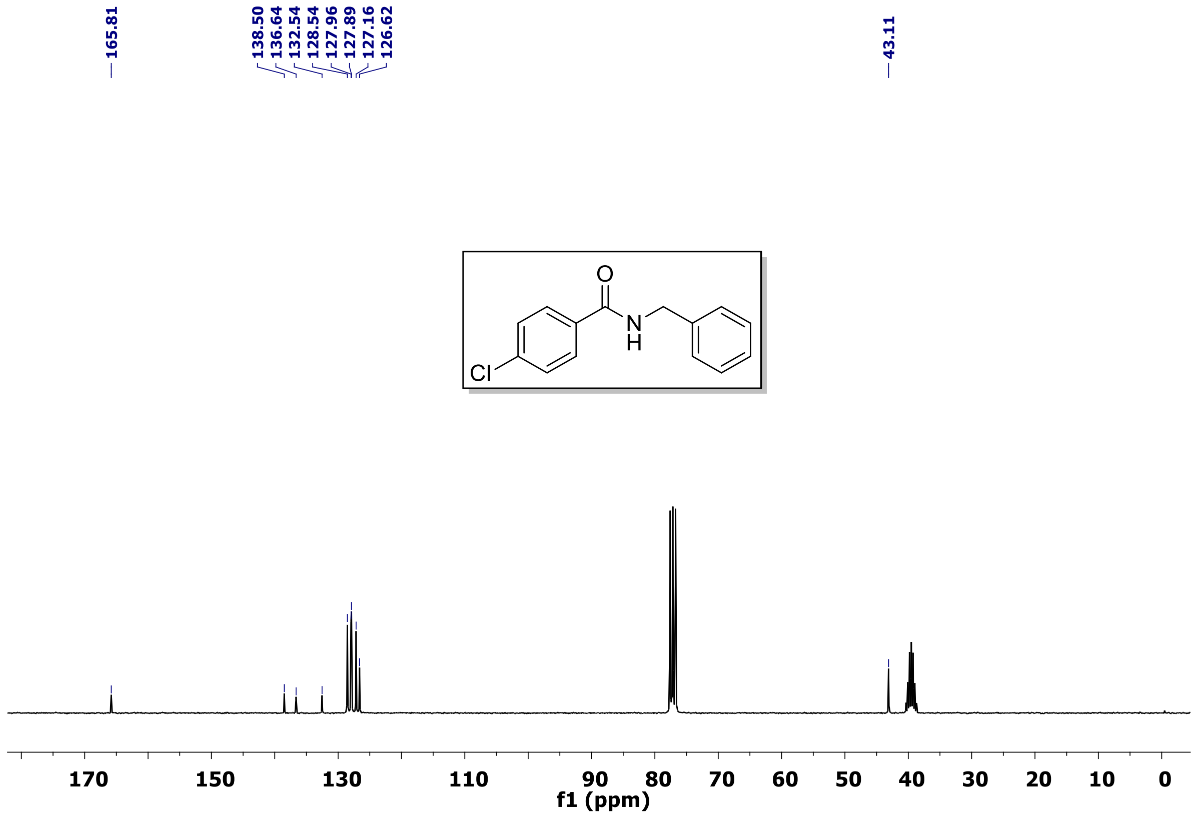

Copies of ^{1}H NMR and ^{13}C NMR spectra of **3o**

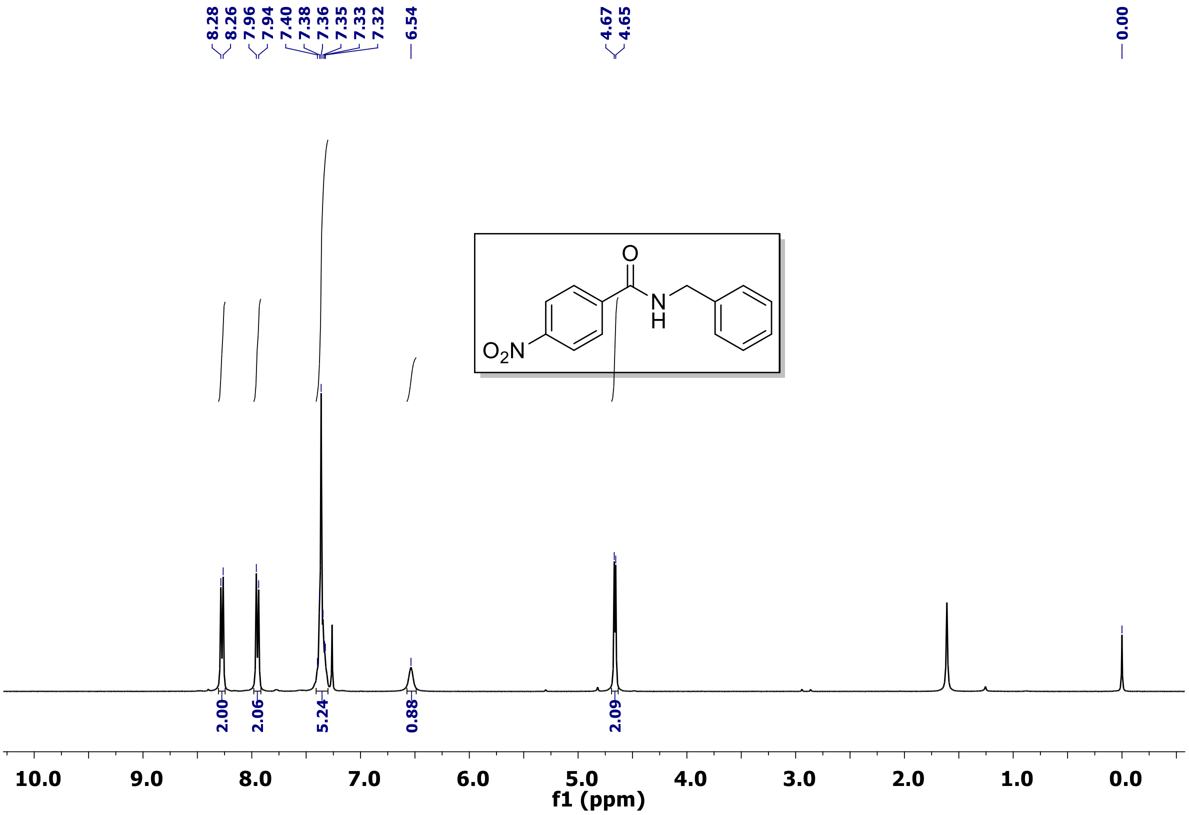

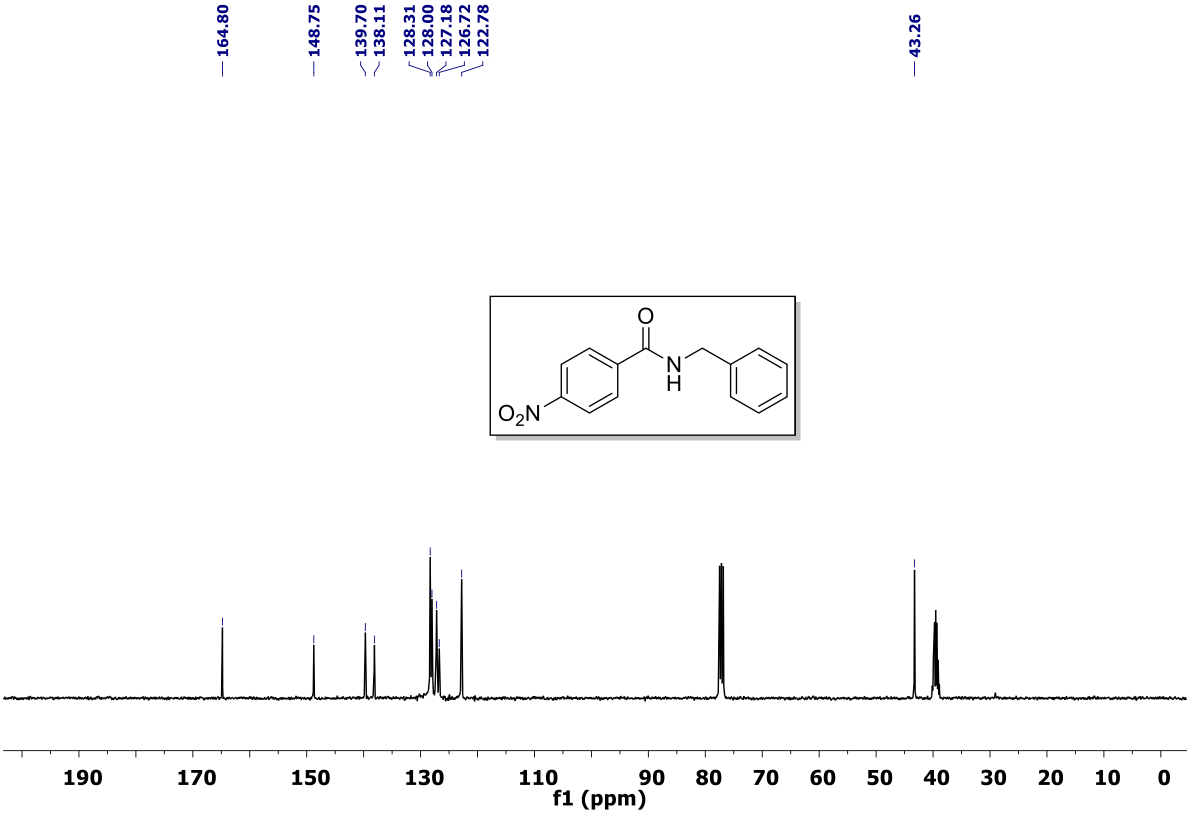

Copies of ^{1}H NMR and ^{13}C NMR spectra of **3p**

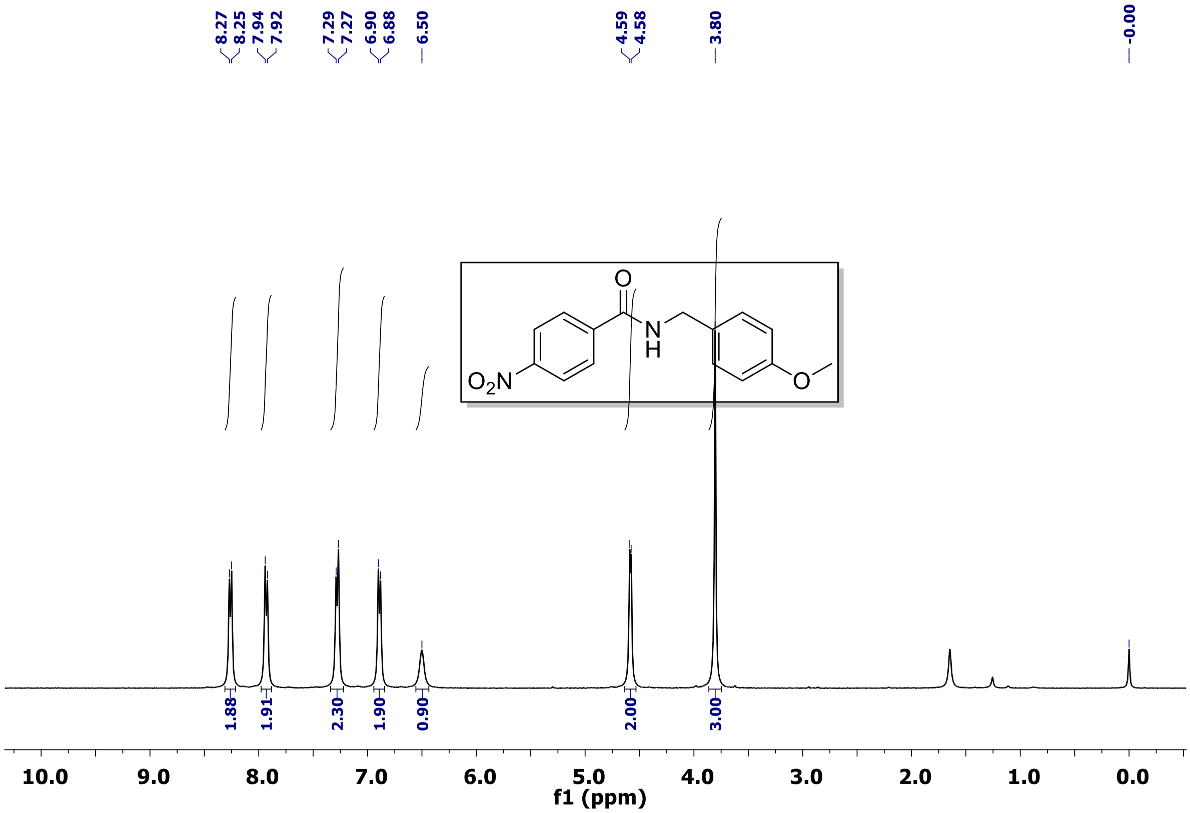

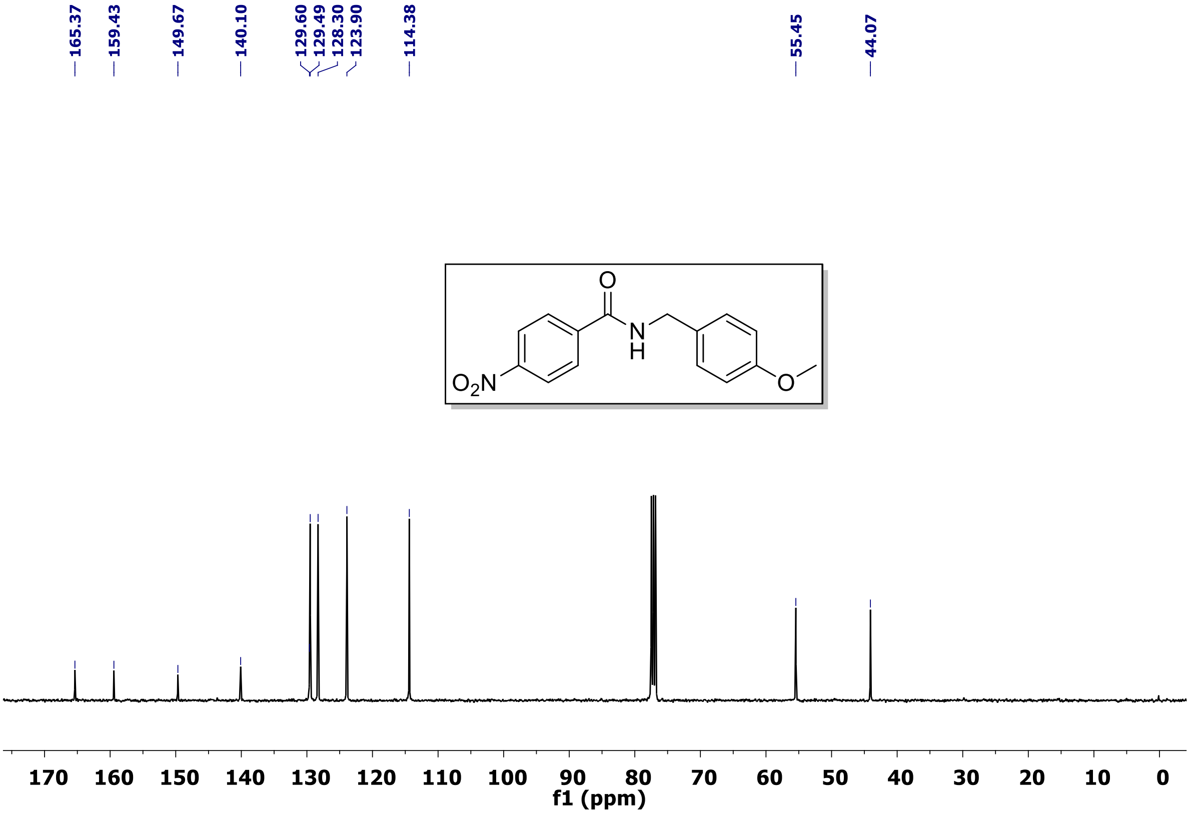

Copies of ^{1}H NMR and ^{13}C NMR spectra of **3q**

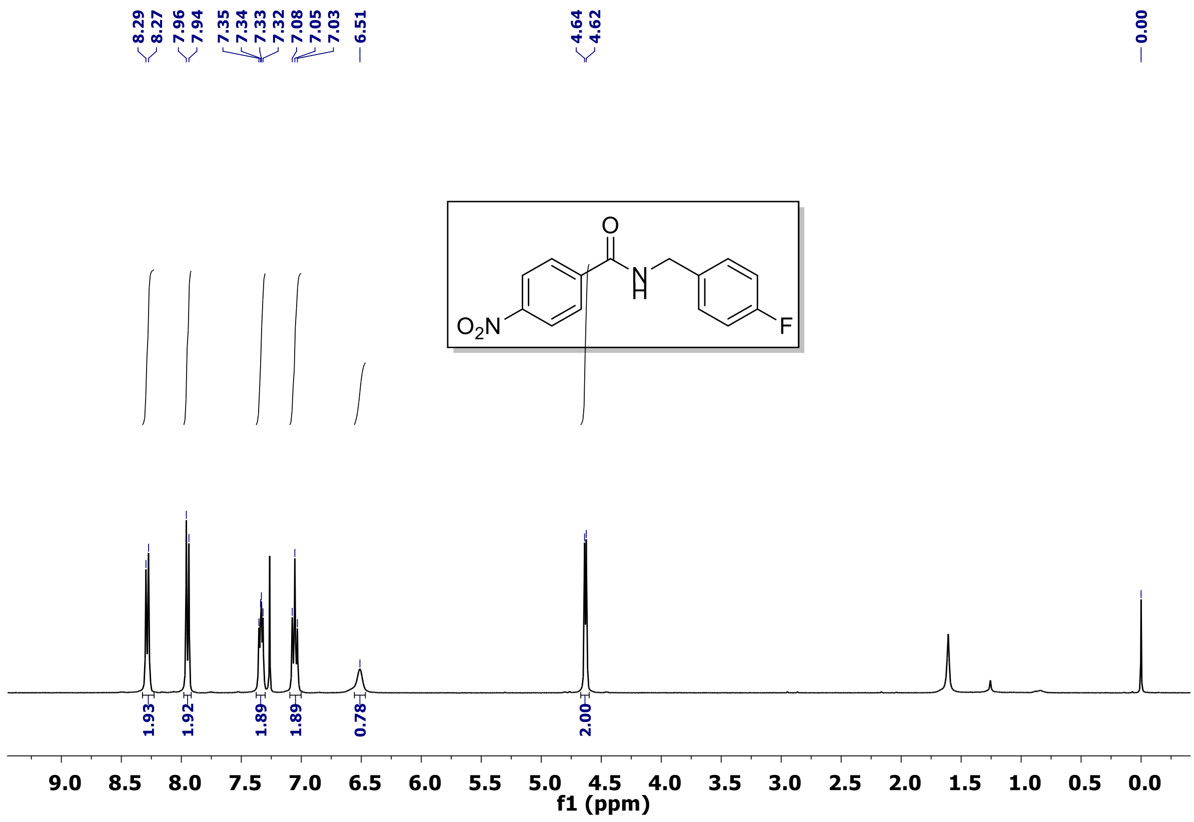

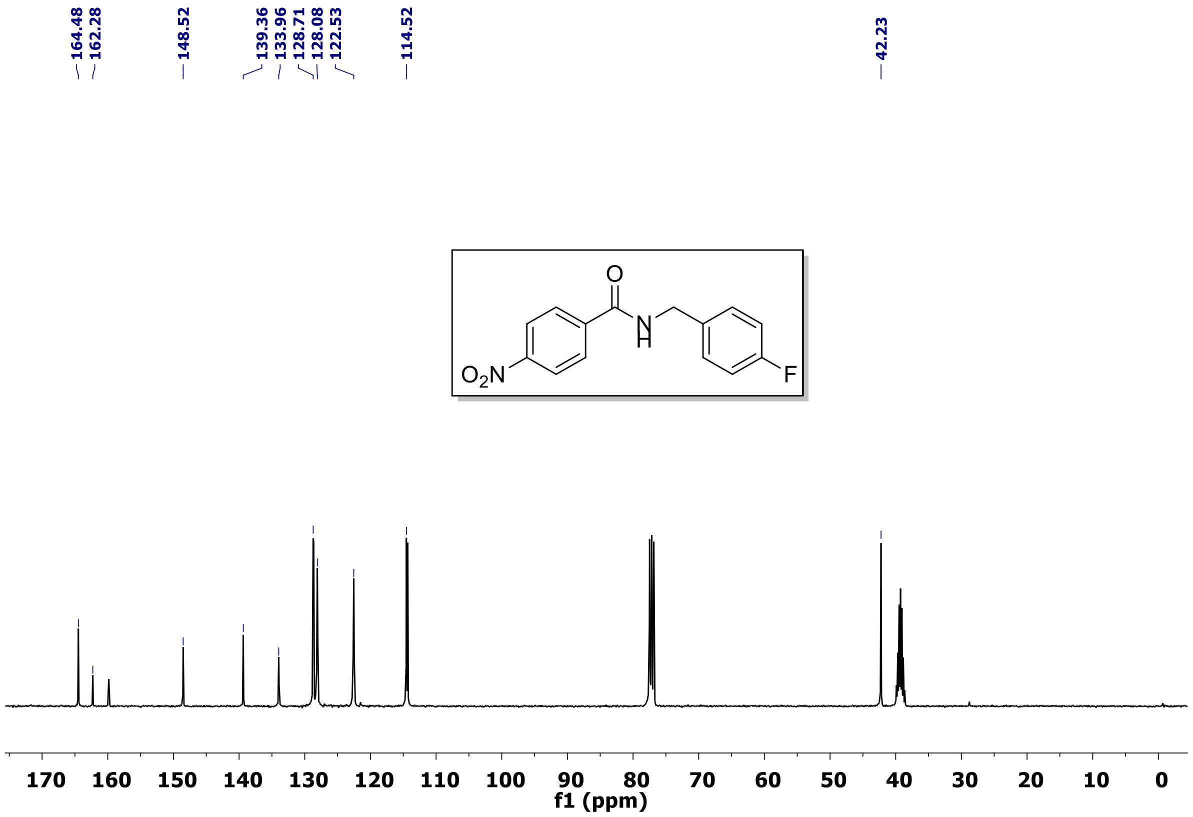

Copies of ^{1}H NMR and ^{13}C NMR spectra of **3r**

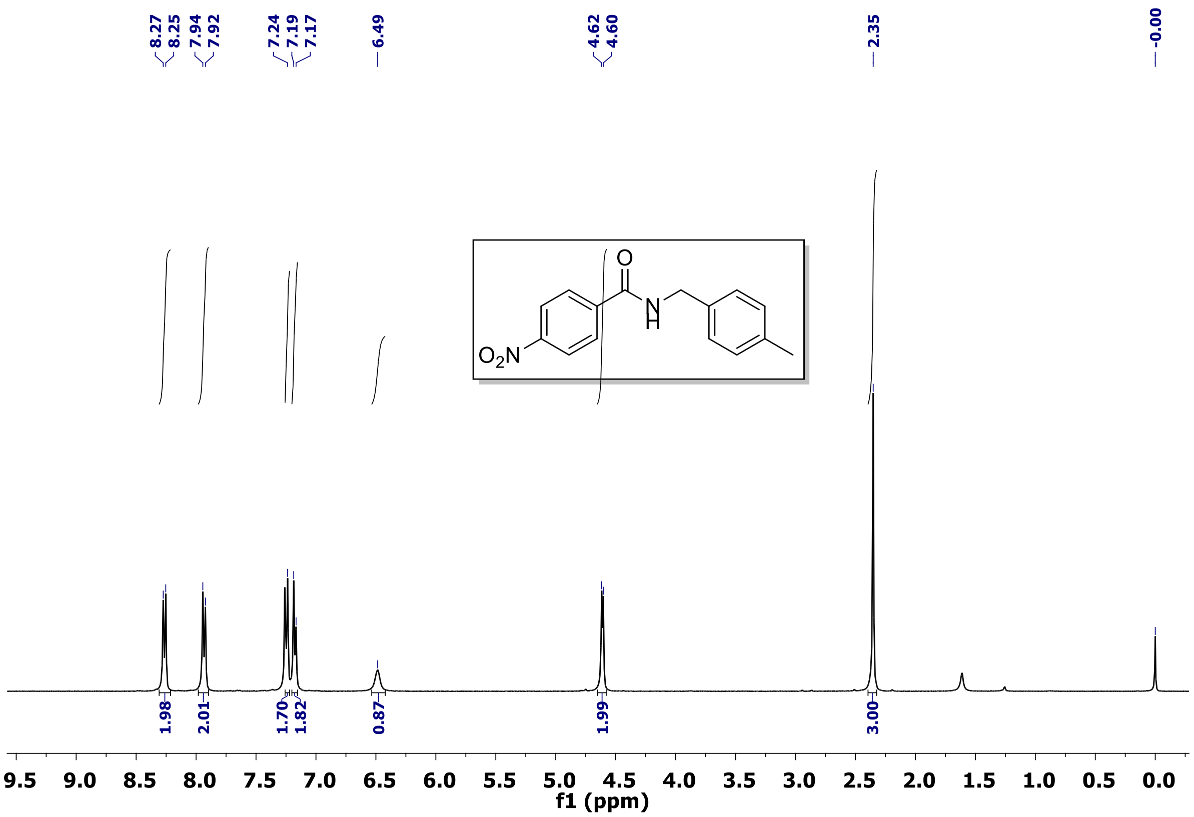

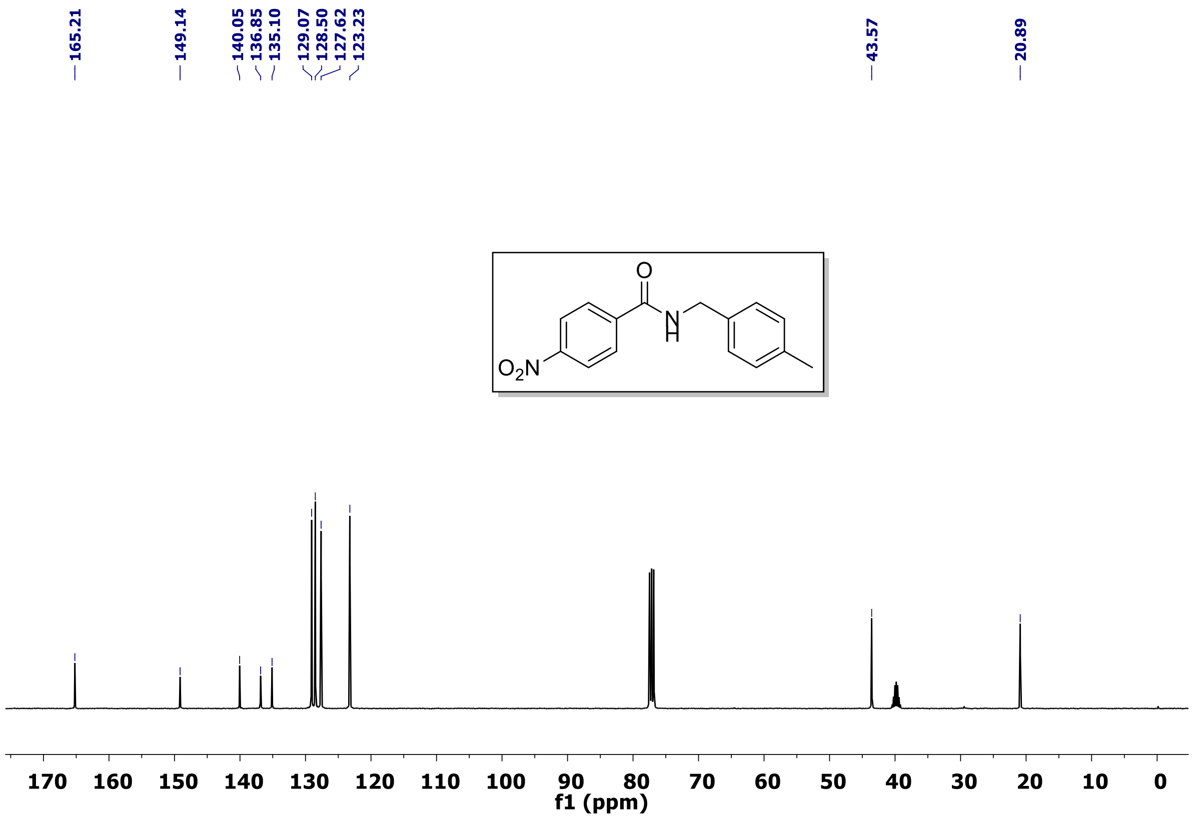

Copies of ^{1}H NMR and ^{13}C NMR spectra of **3s**

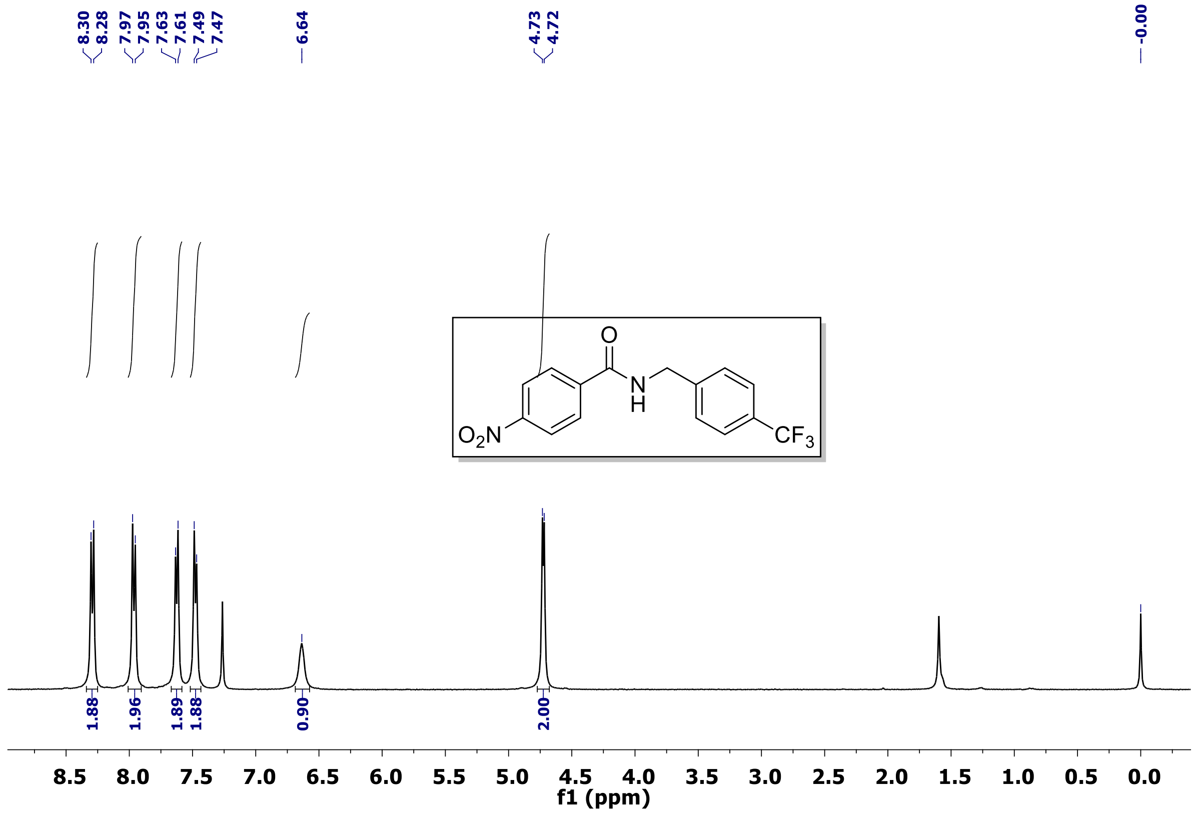

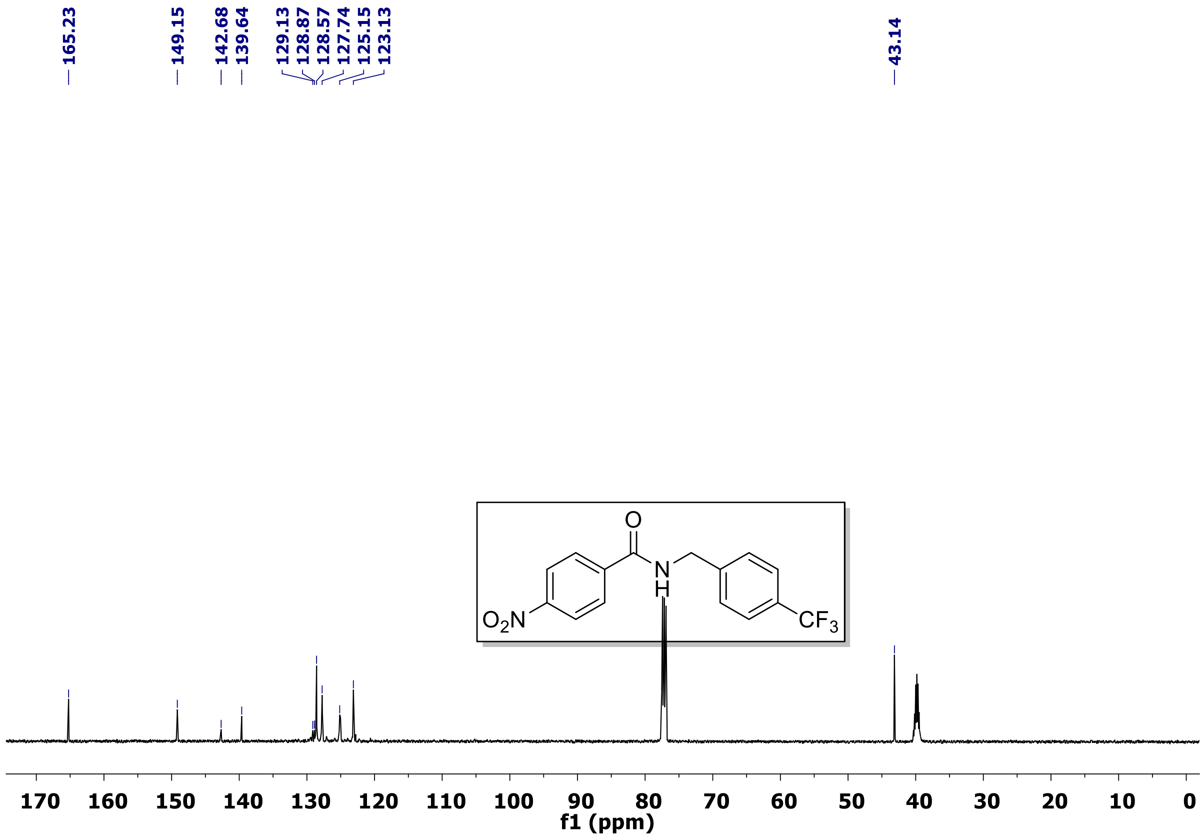

Copies of ^{1}H NMR and ^{13}C NMR spectra of **3t**

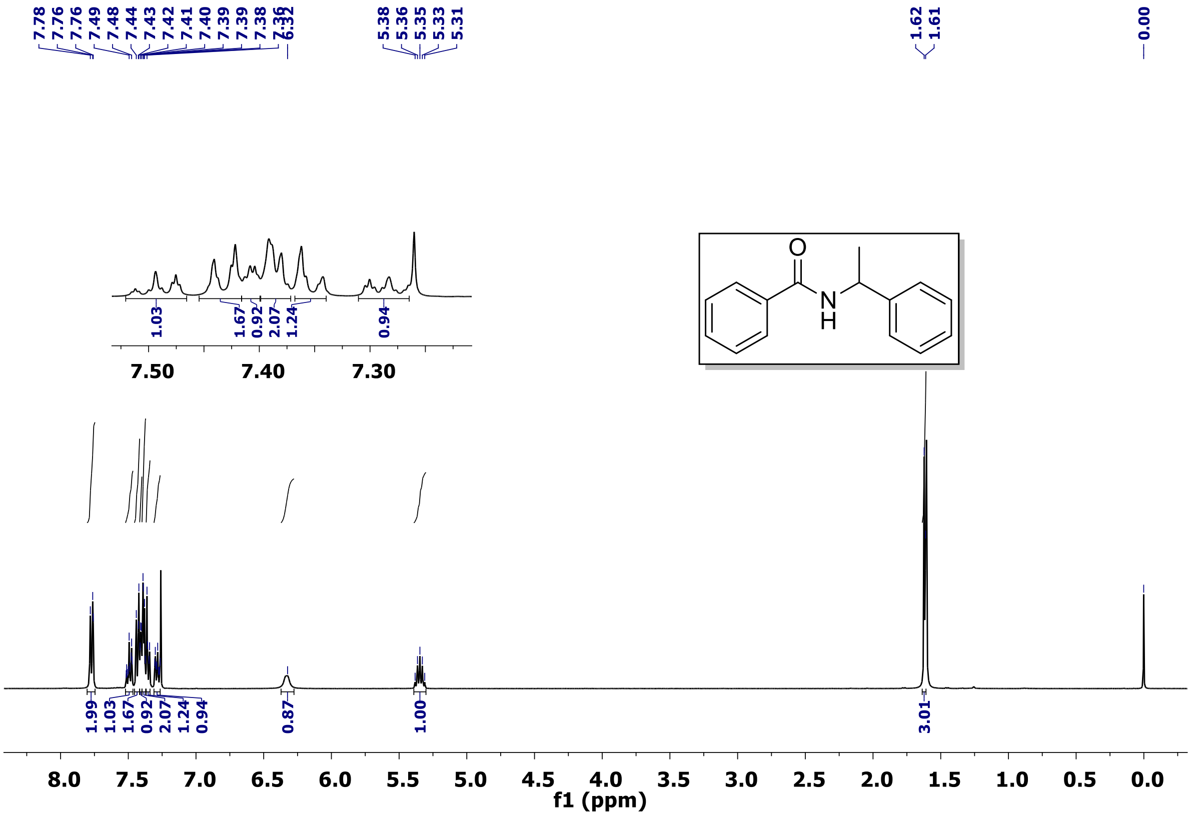

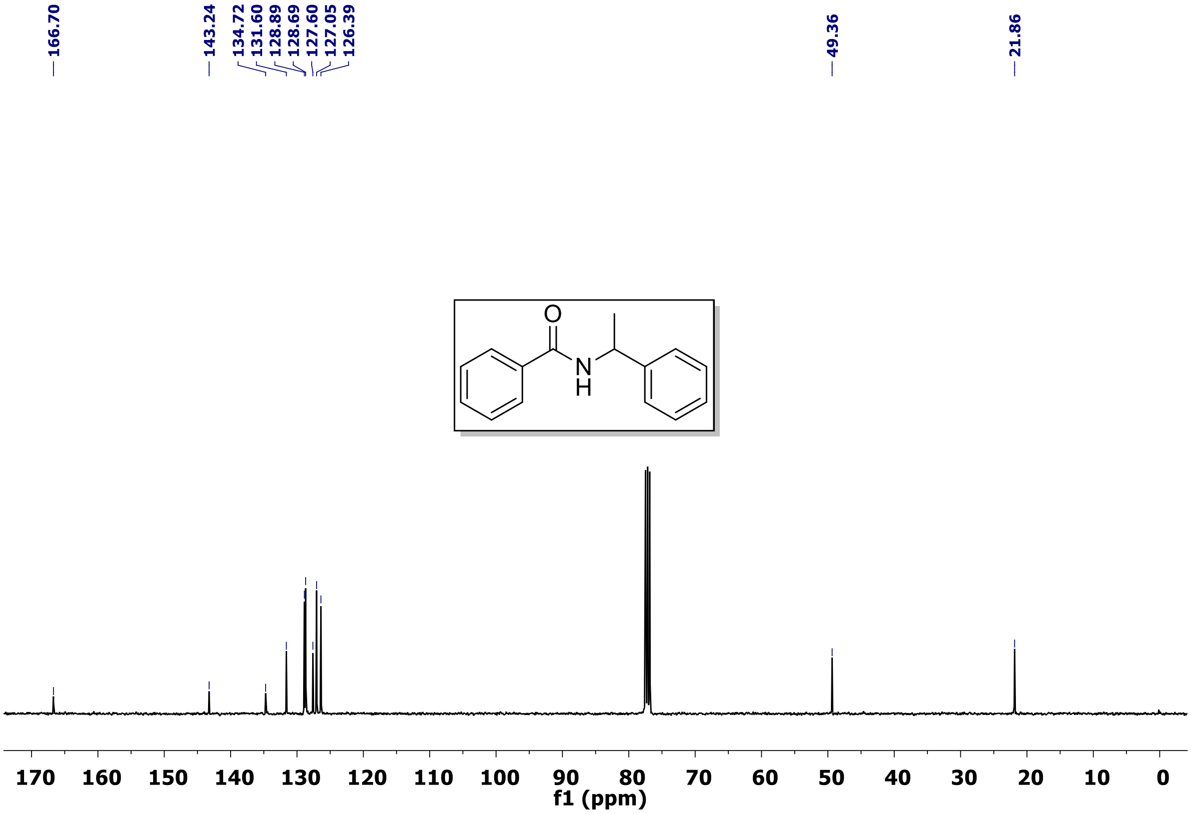

Copies of ^{1}H NMR and ^{13}C NMR spectra of **3u**

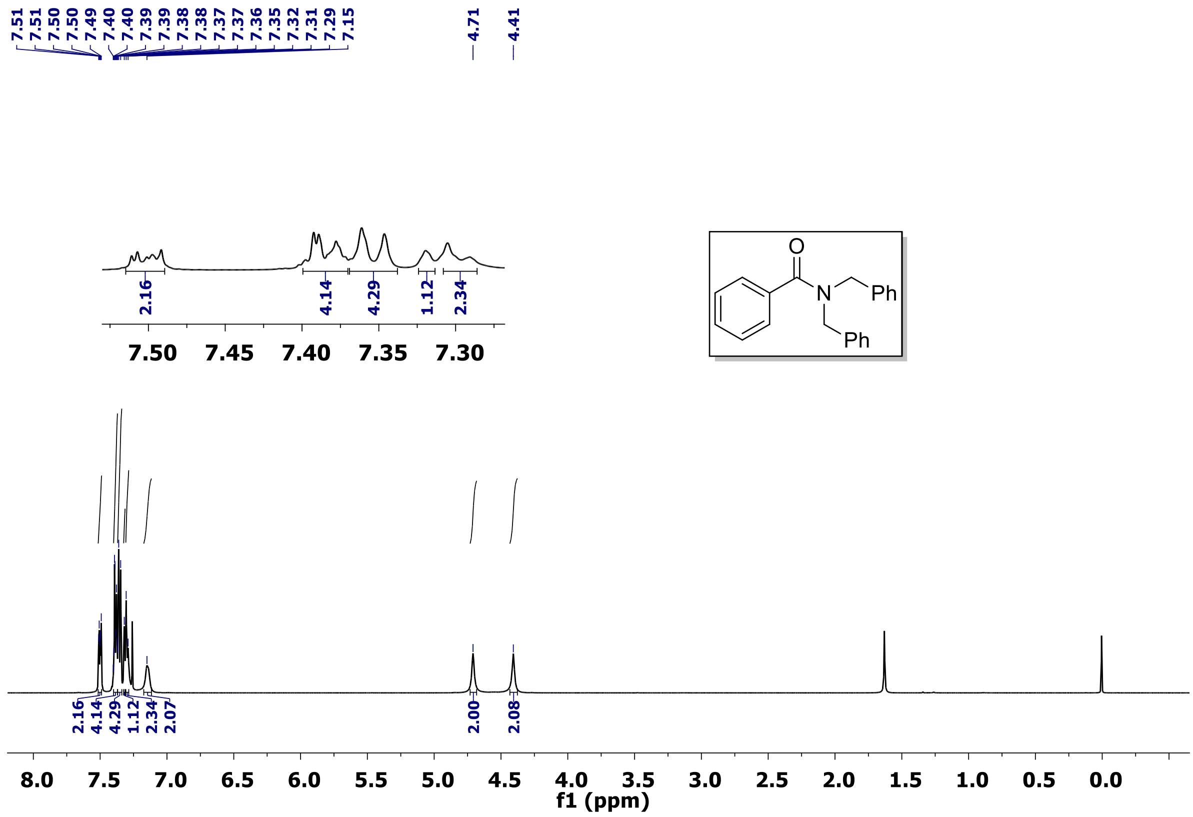

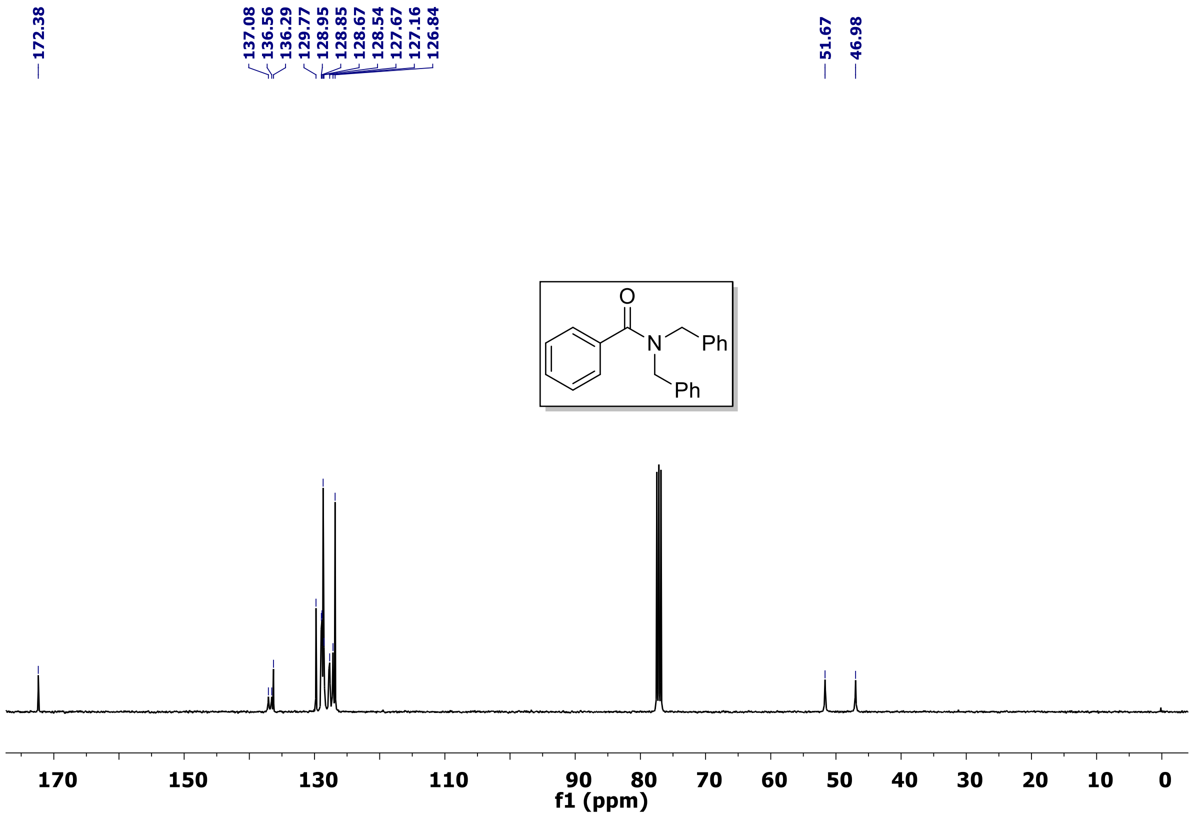

Copies of ^{1}H NMR and ^{13}C NMR spectra of **5a**

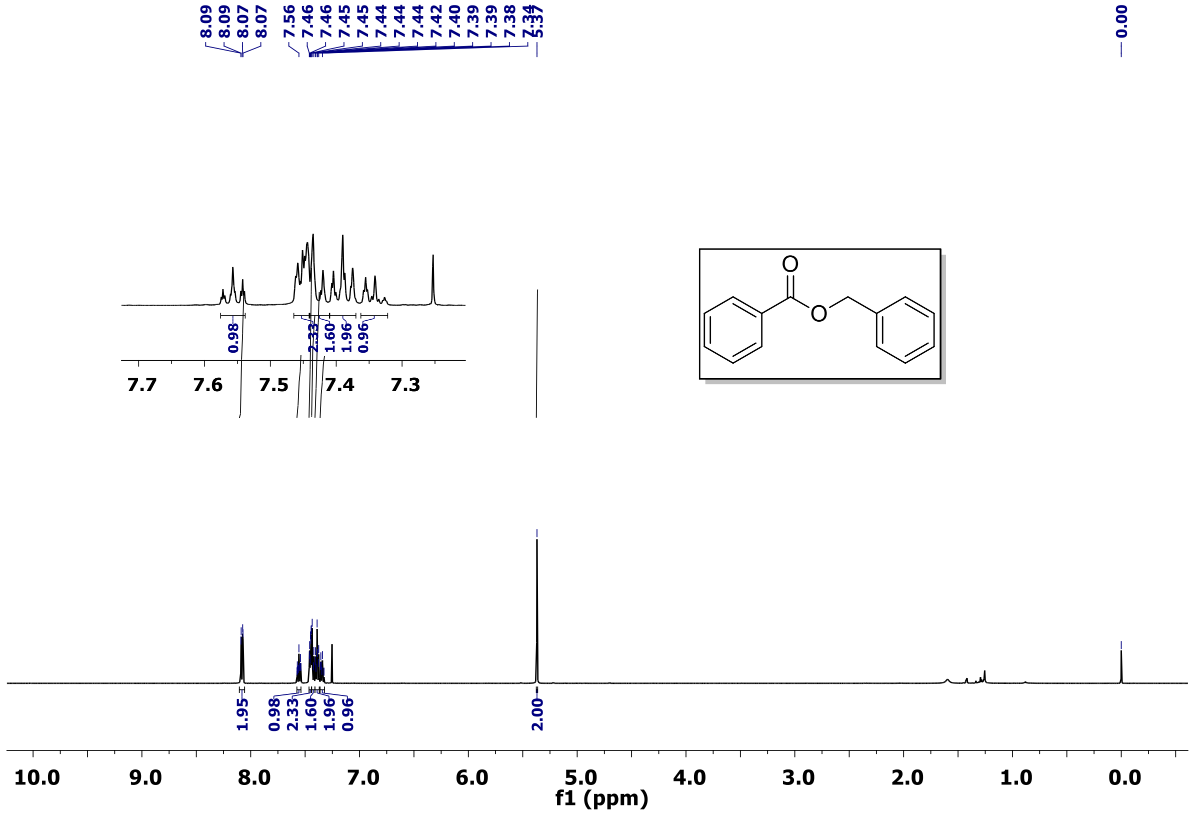

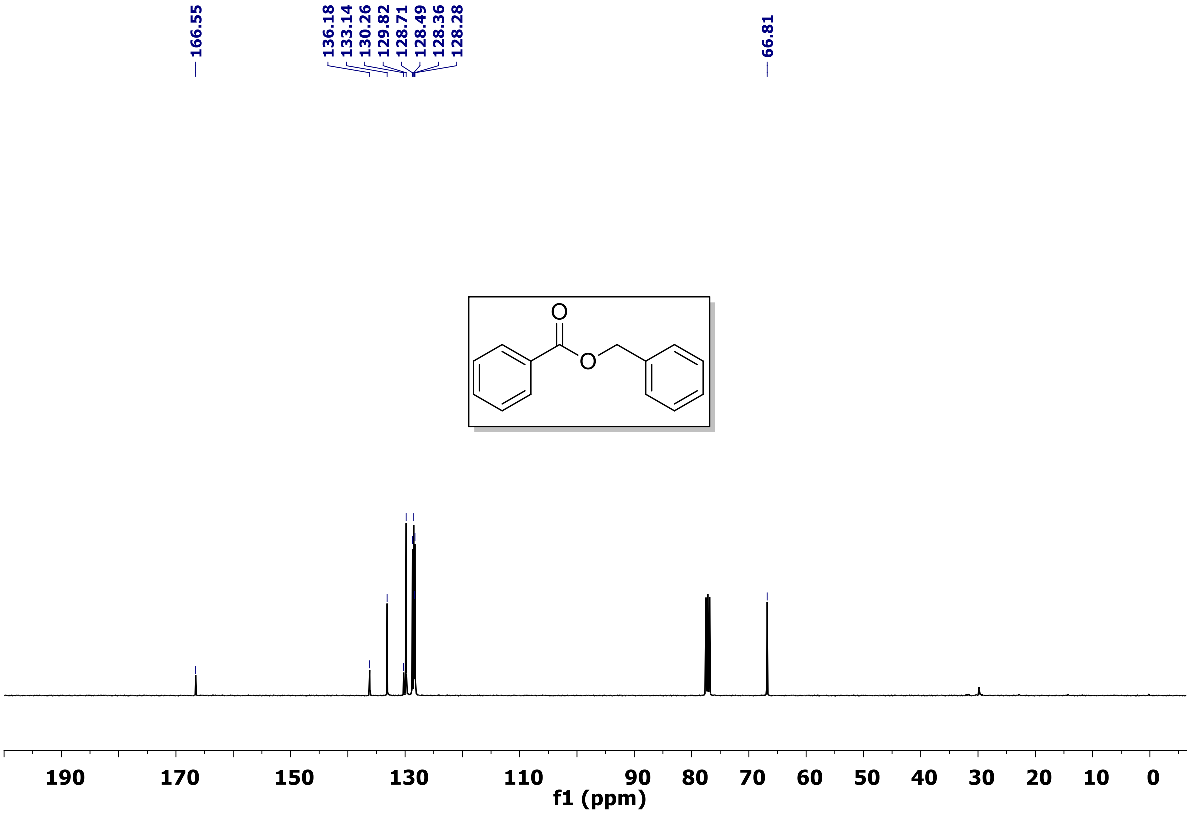

Copies of ^{1}H NMR and ^{13}C NMR spectra of **5b**

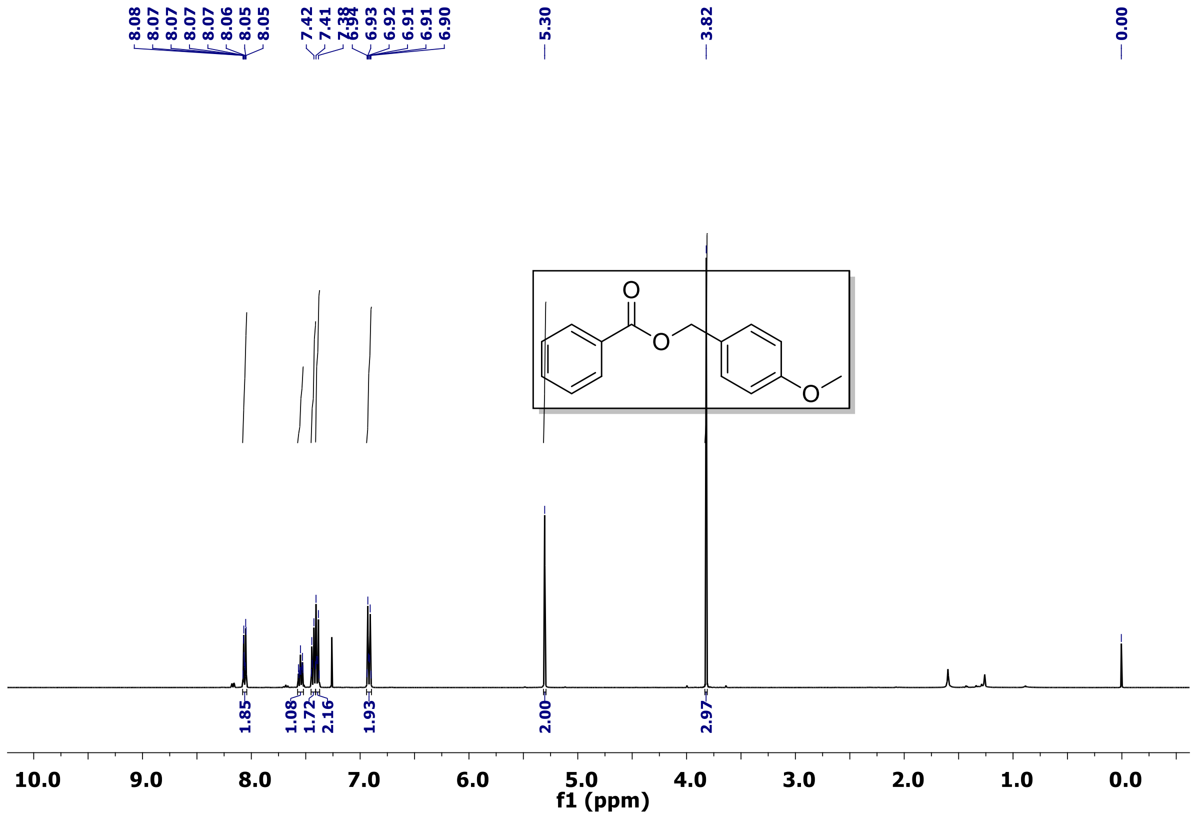

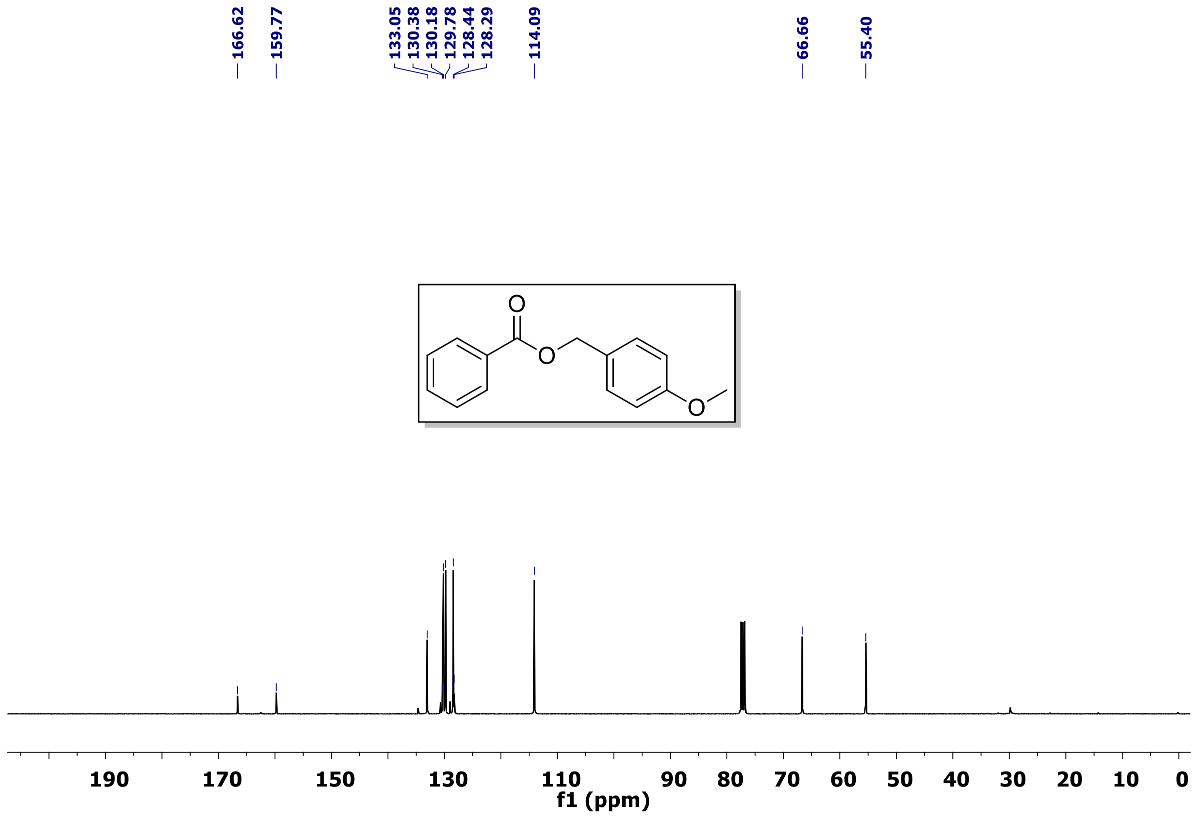

Copies of ^{1}H NMR and ^{13}C NMR spectra of **5c**

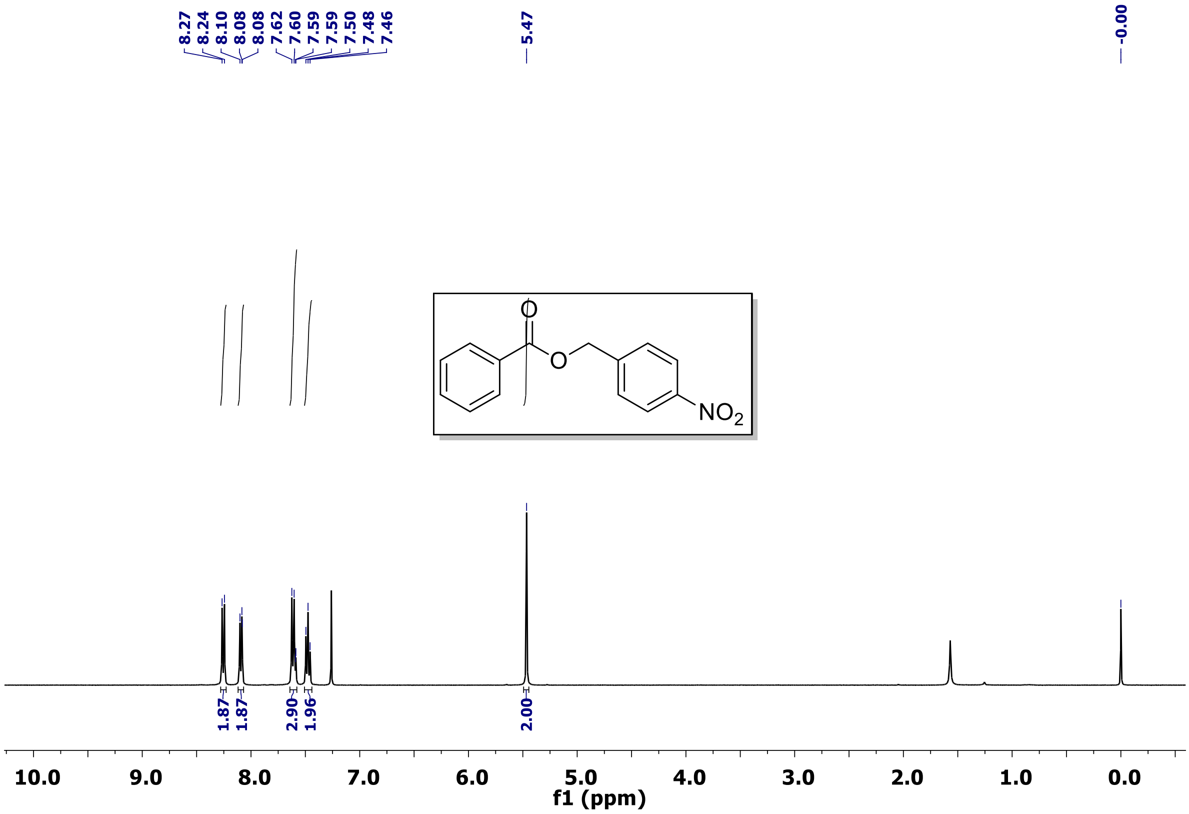

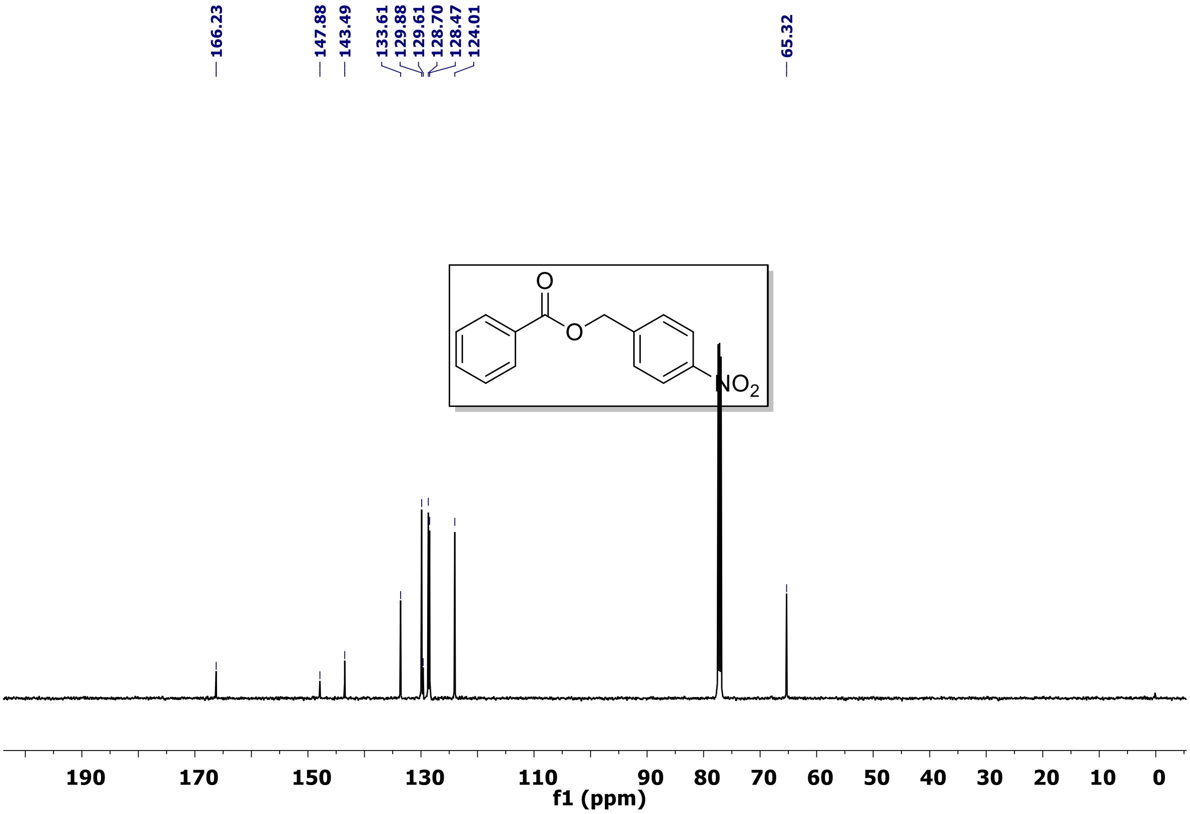

Copies of ^{1}H NMR and ^{13}C NMR spectra of **5d**

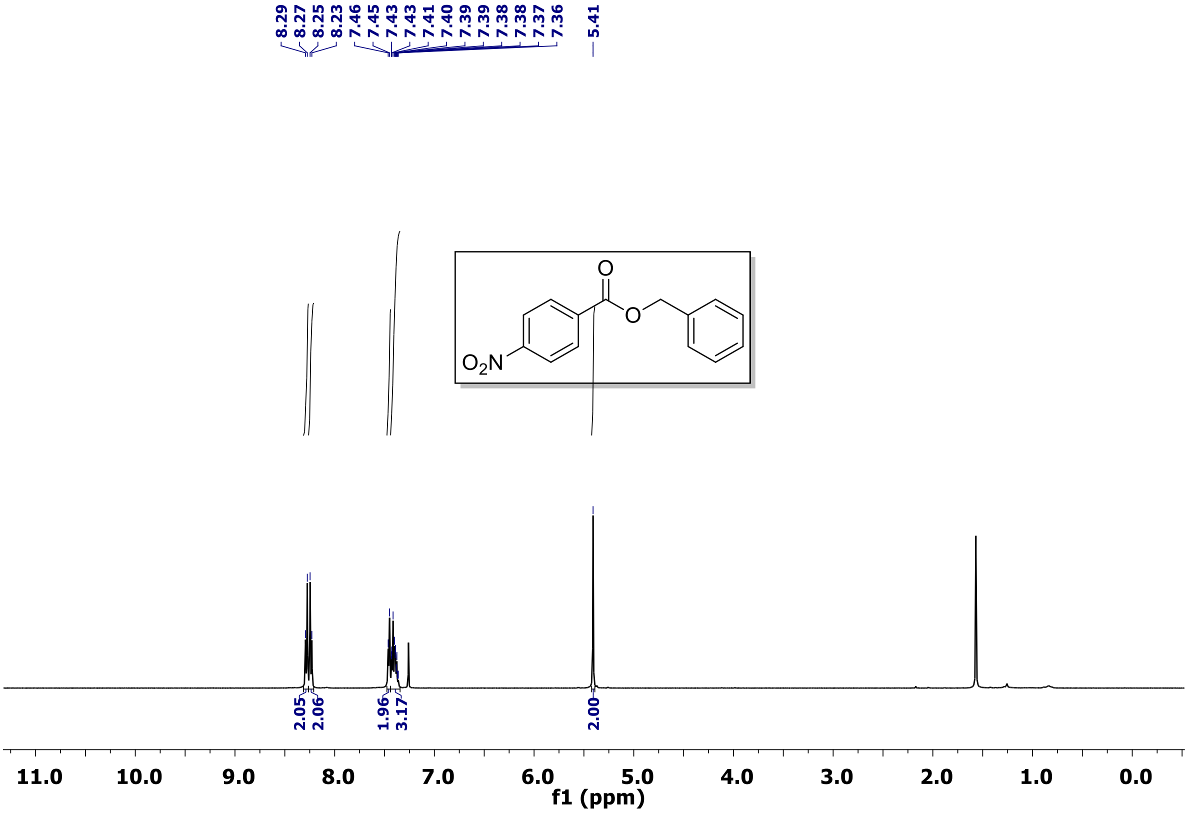

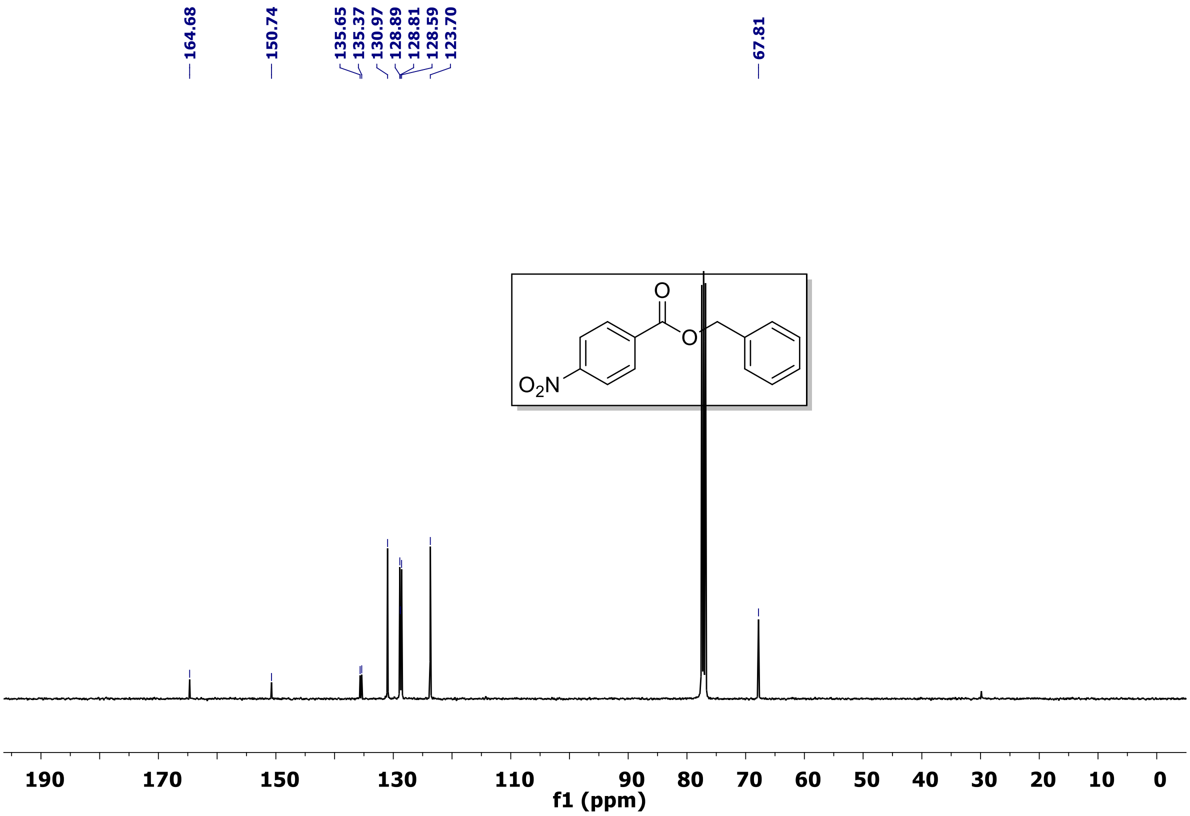

Copies of ^{1}H NMR and ^{13}C NMR spectra of **5e**

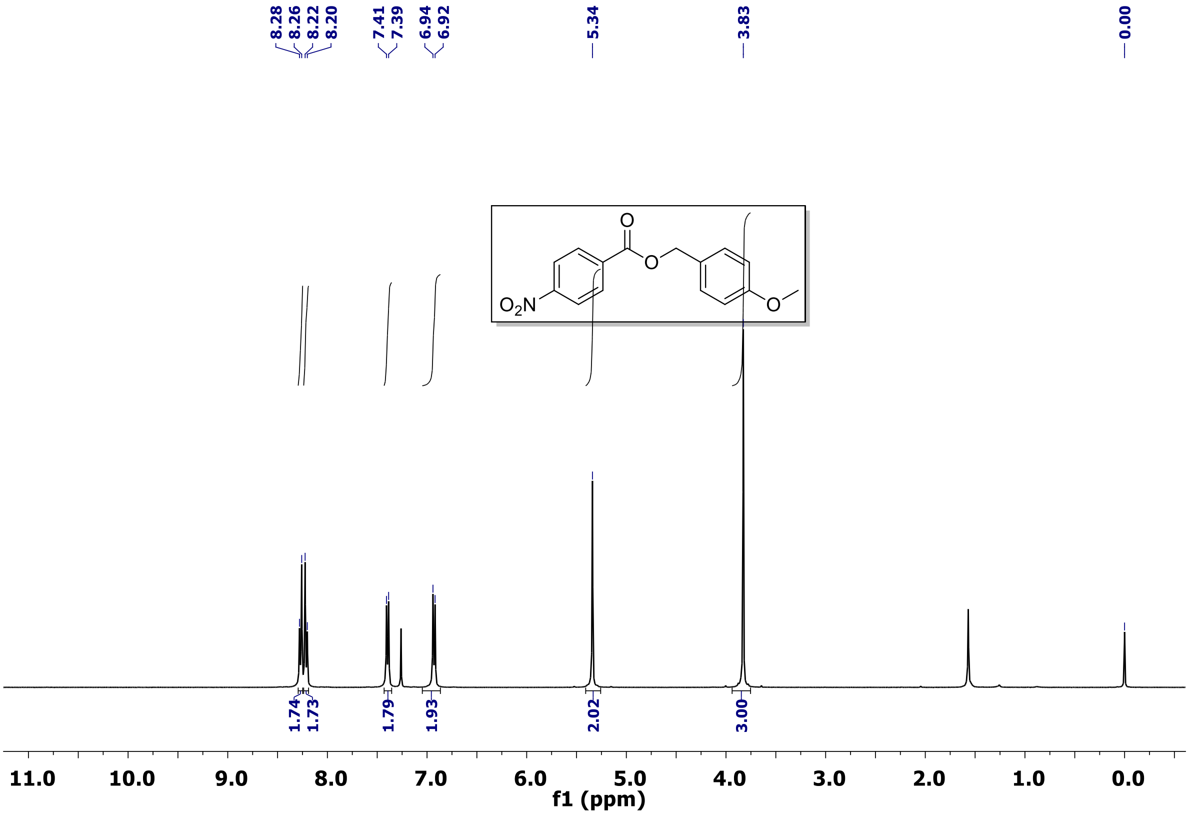

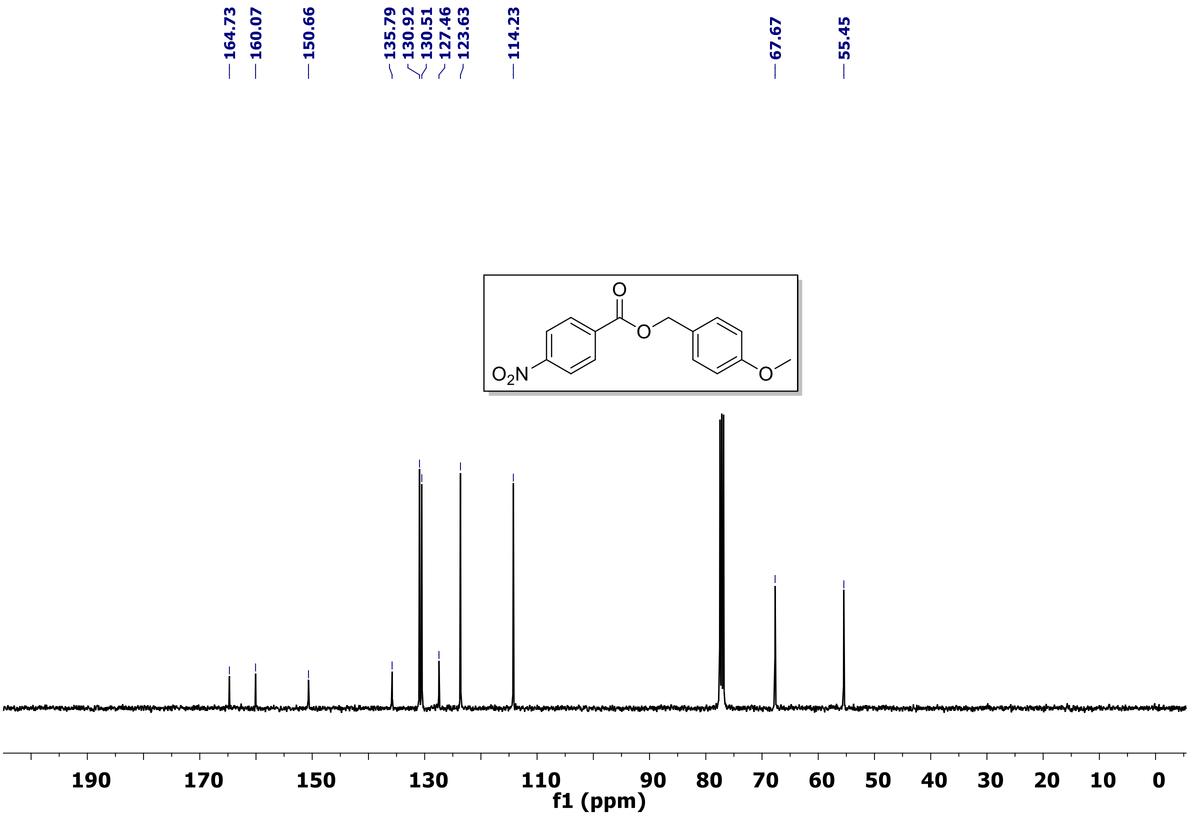

Copies of ^{1}H NMR and ^{13}C NMR spectra of **5f**

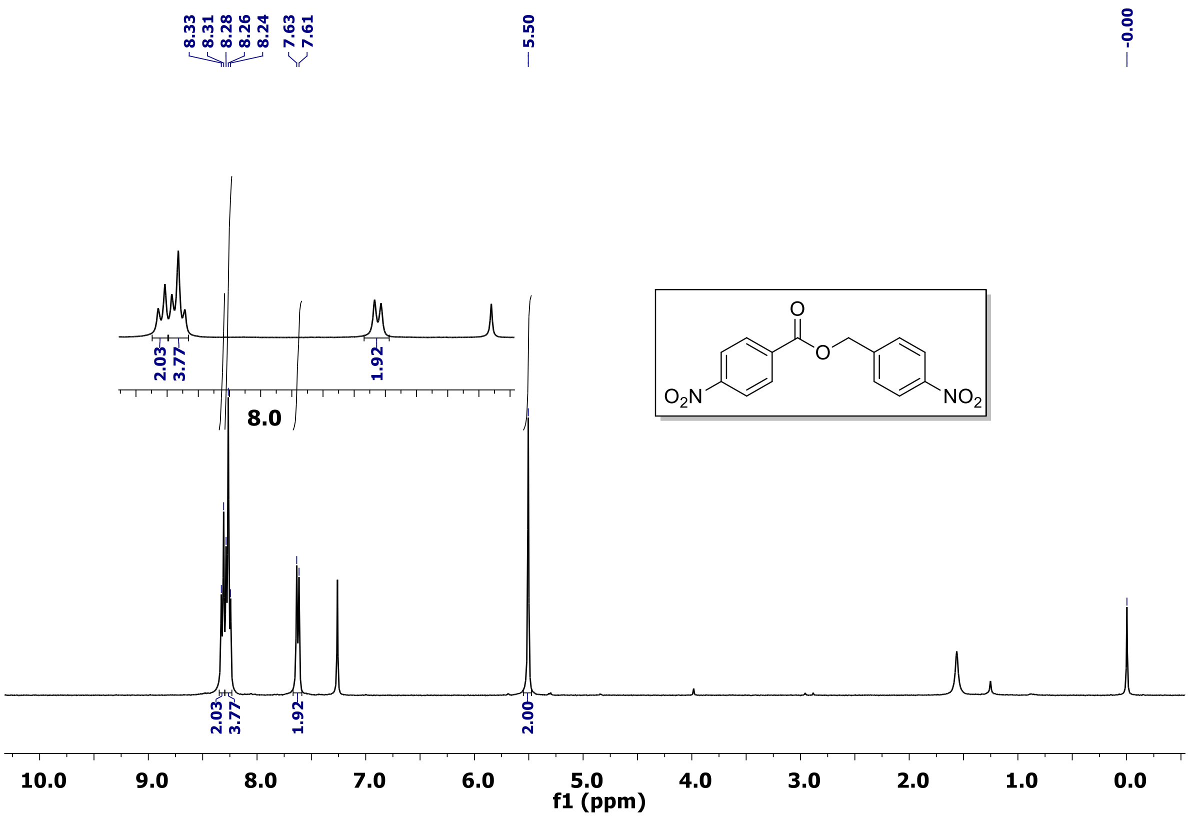

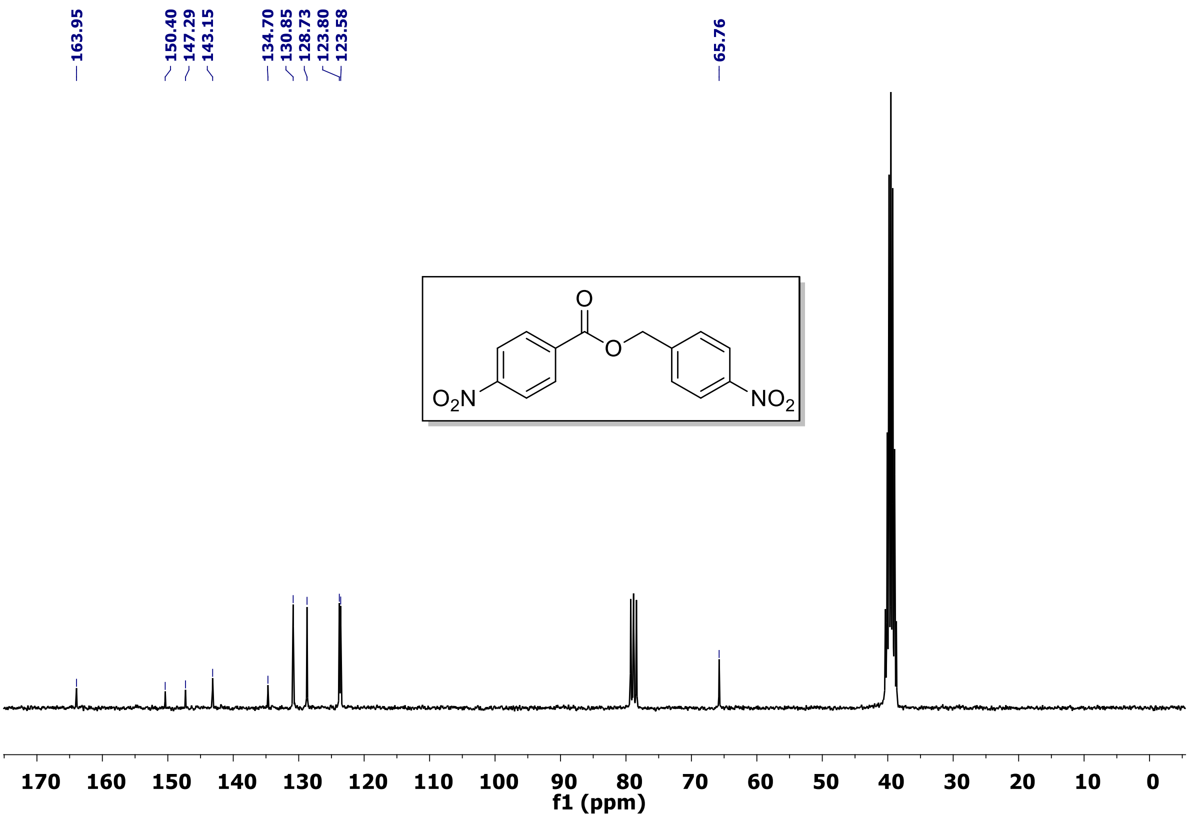

Copies of ^{1}H NMR and ^{13}C NMR spectra of **5g**

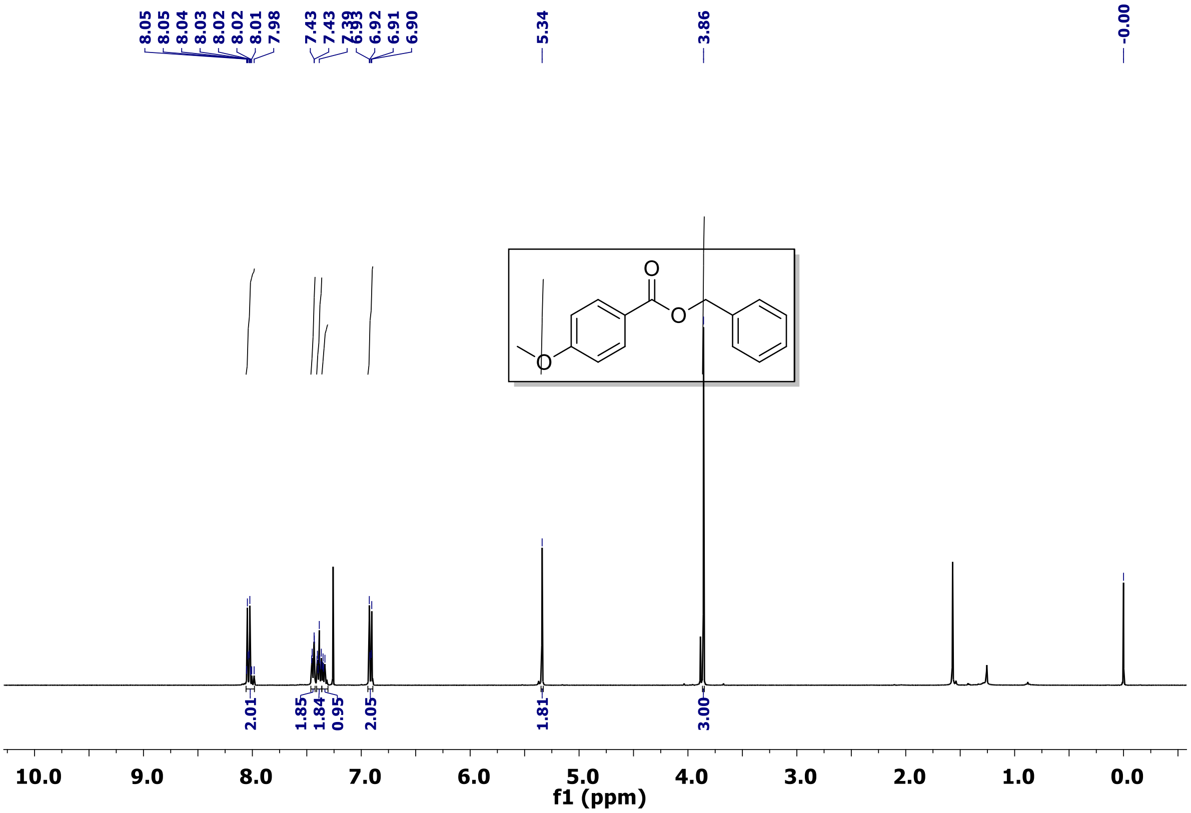

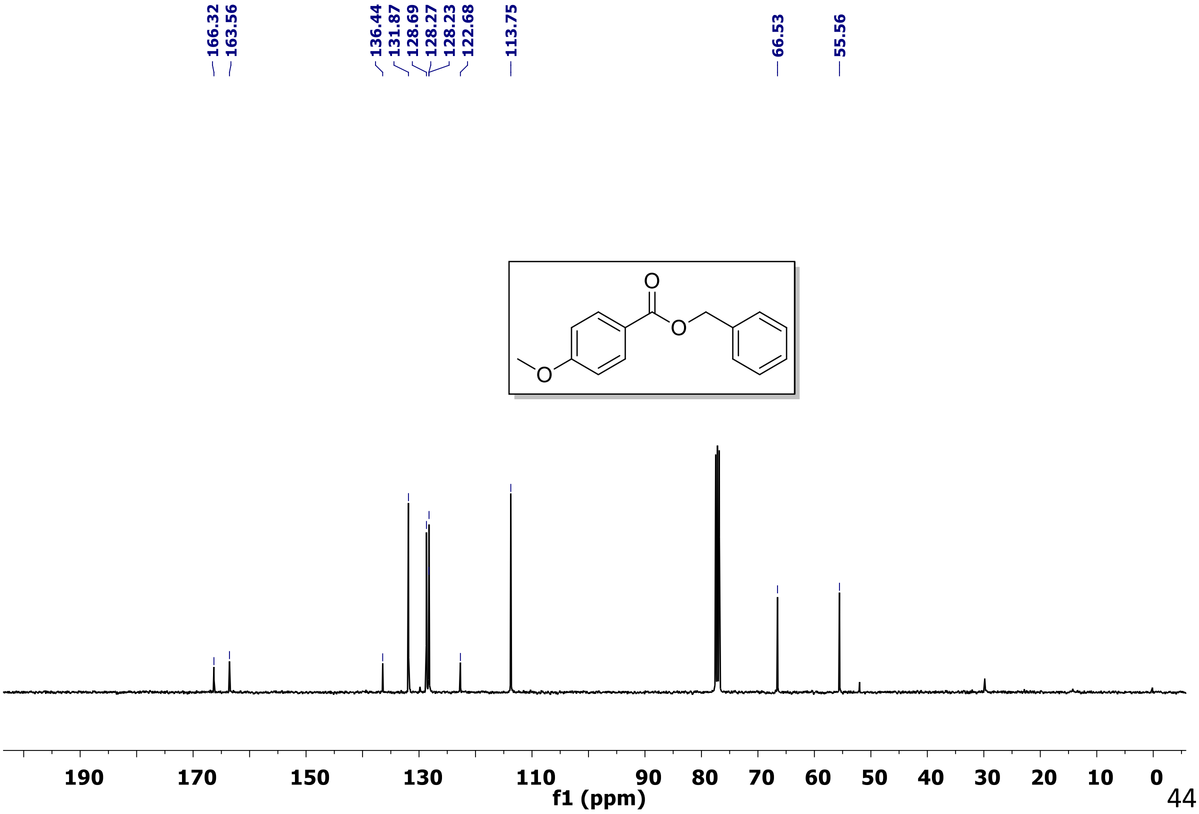

Copies of ^{1}H NMR and ^{13}C NMR spectra of **5h**

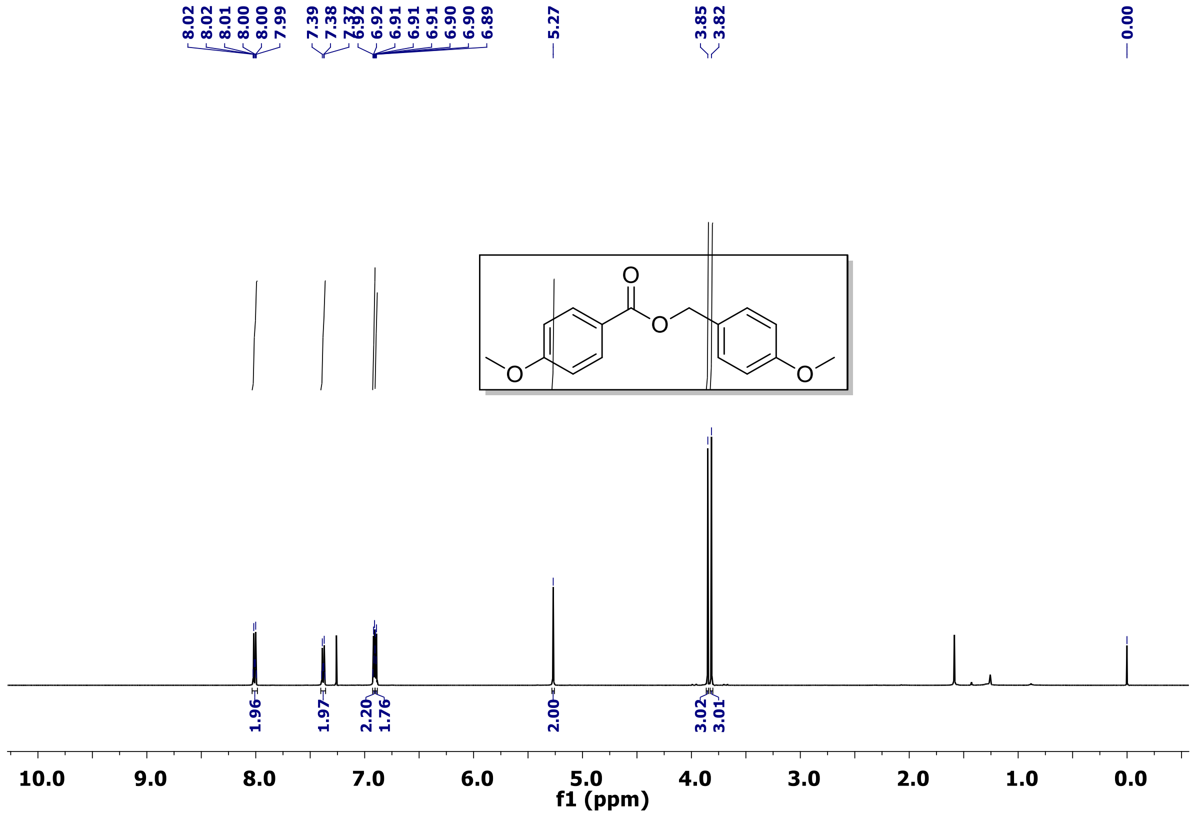

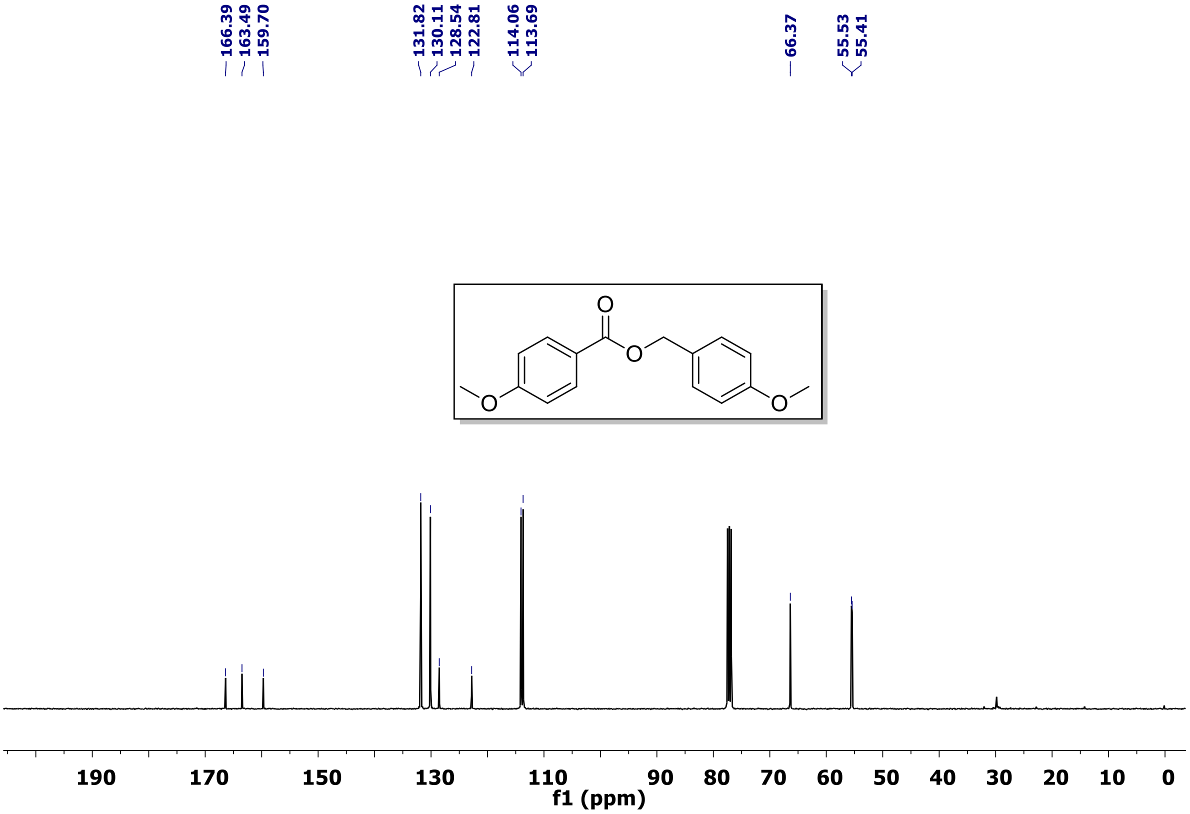

Copies of ^{1}H NMR and ^{13}C NMR spectra of **5i**

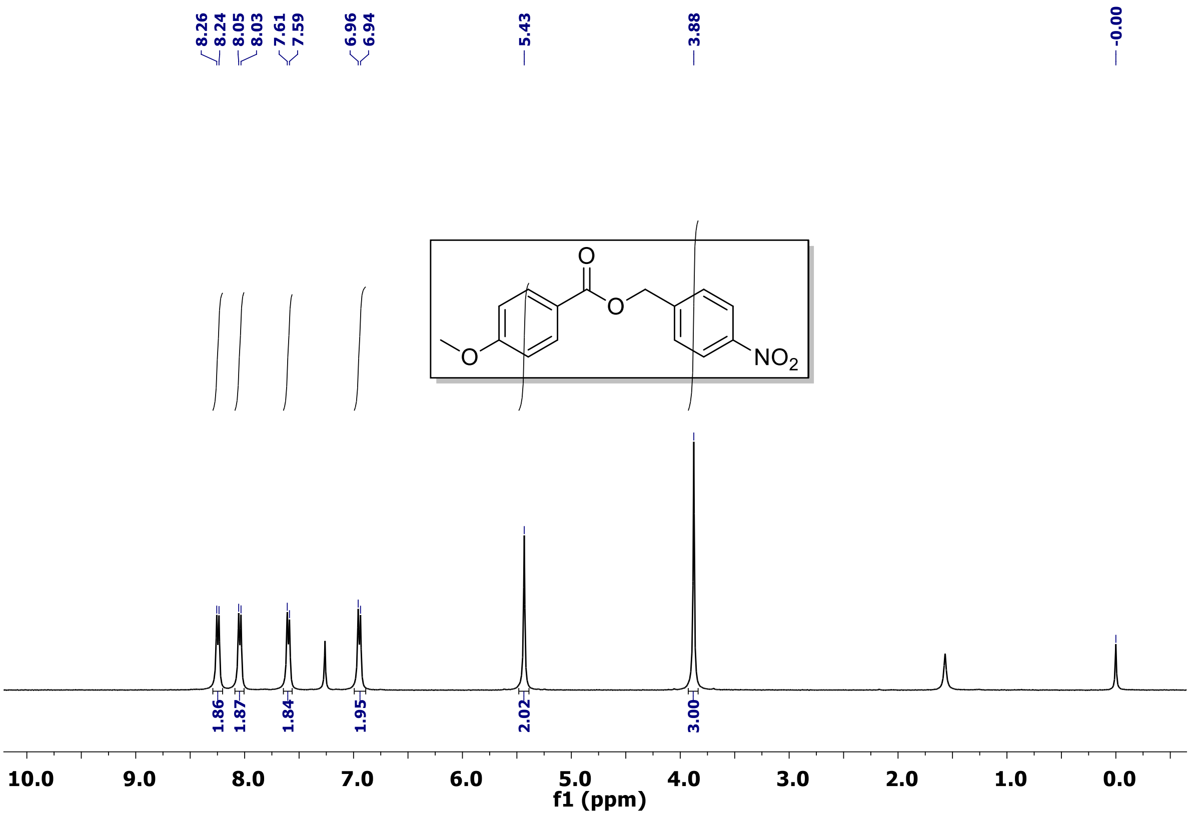

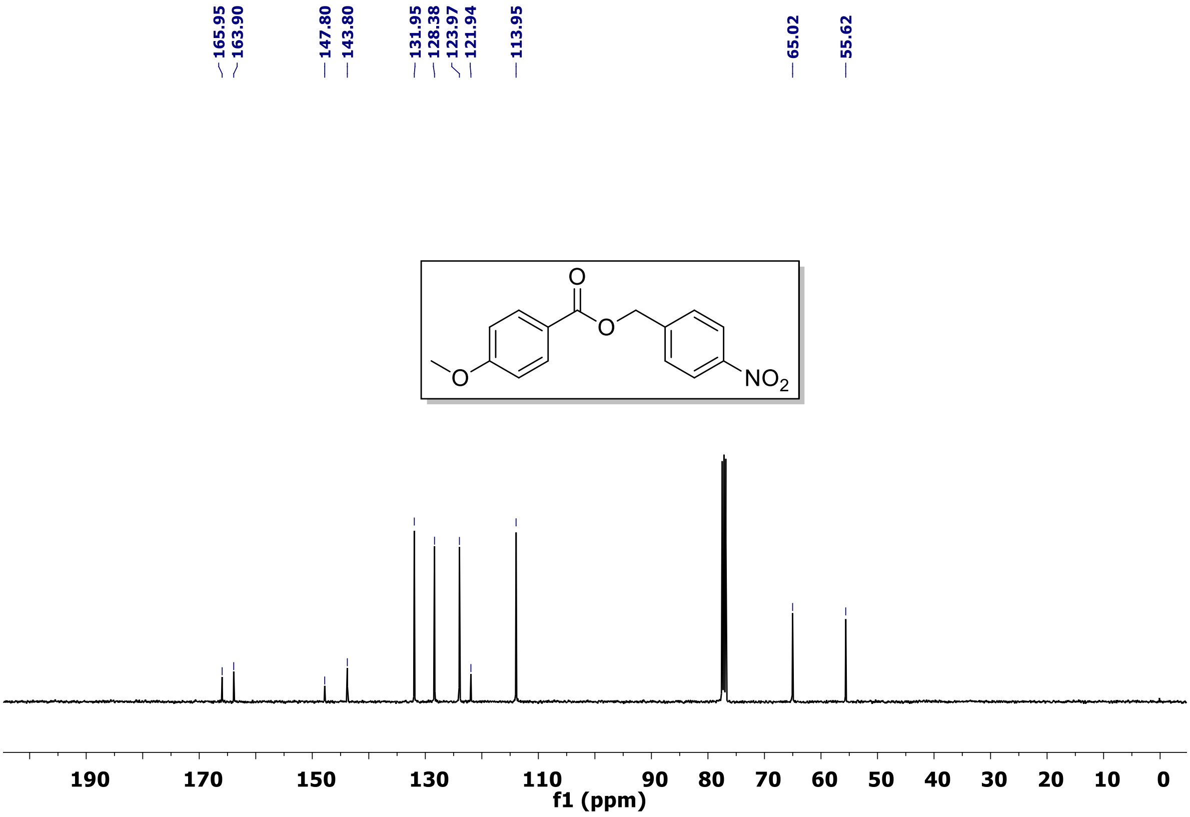

Copies of ^{1}H NMR and ^{13}C NMR spectra of **7a**

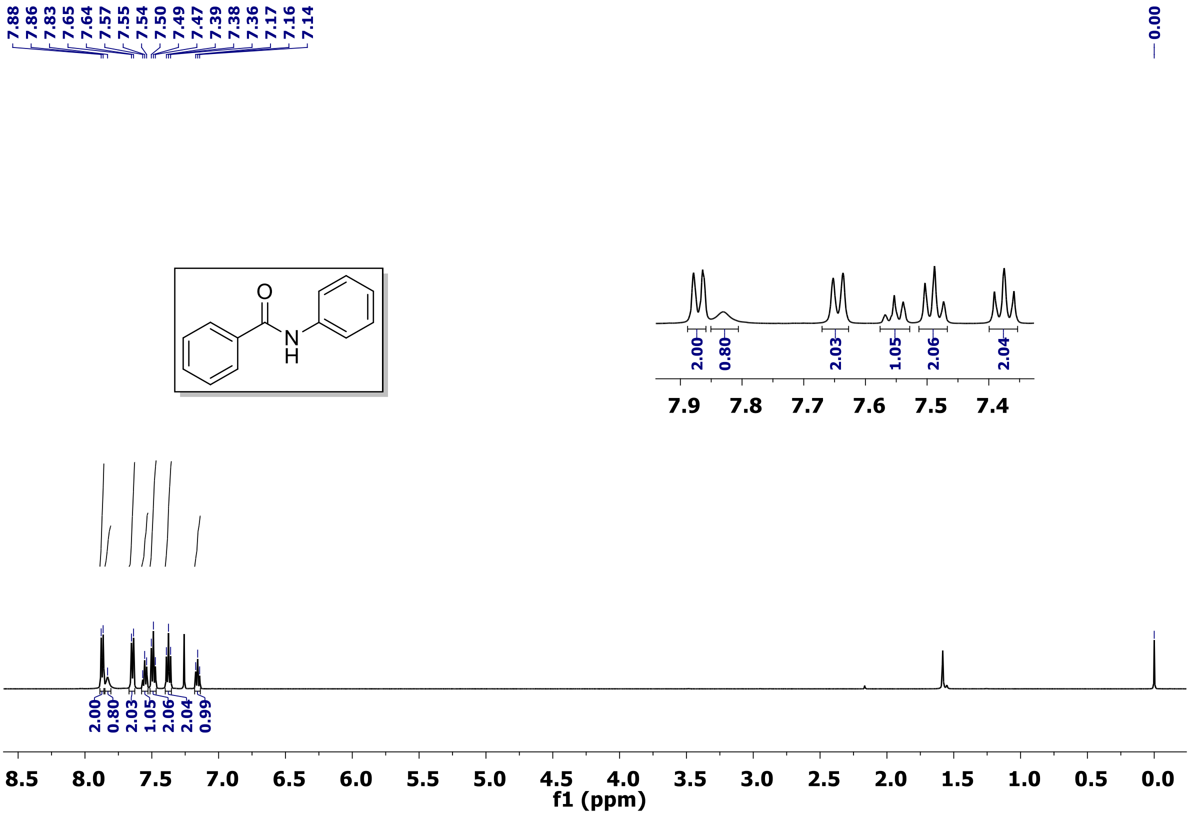

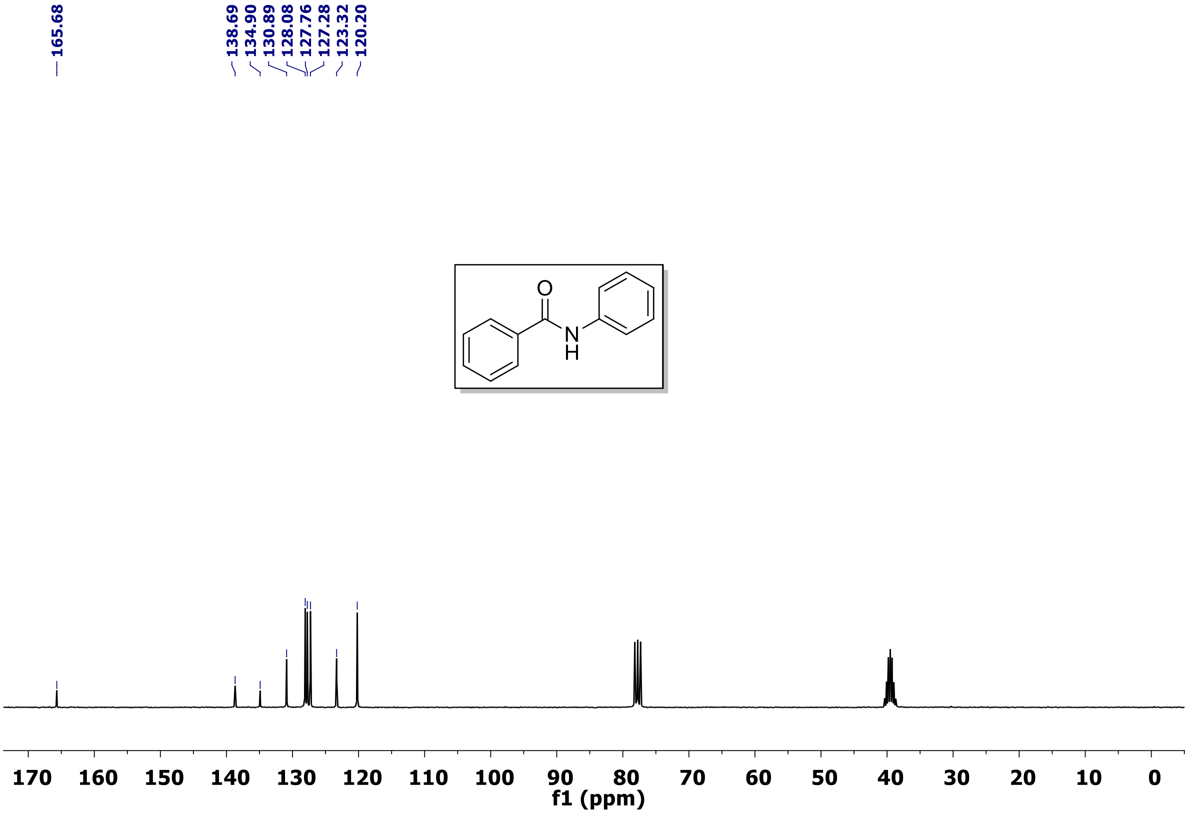

Copies of ^{1}H NMR and ^{13}C NMR spectra of **7b**

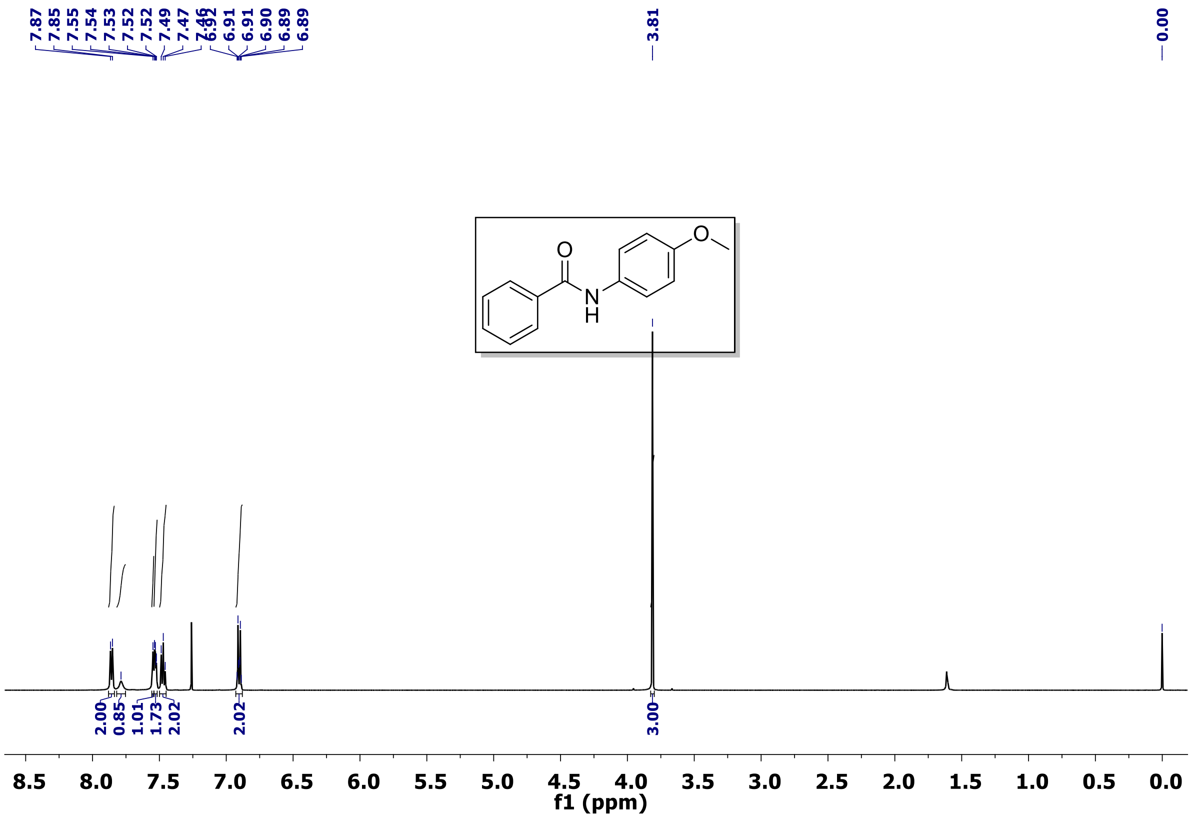

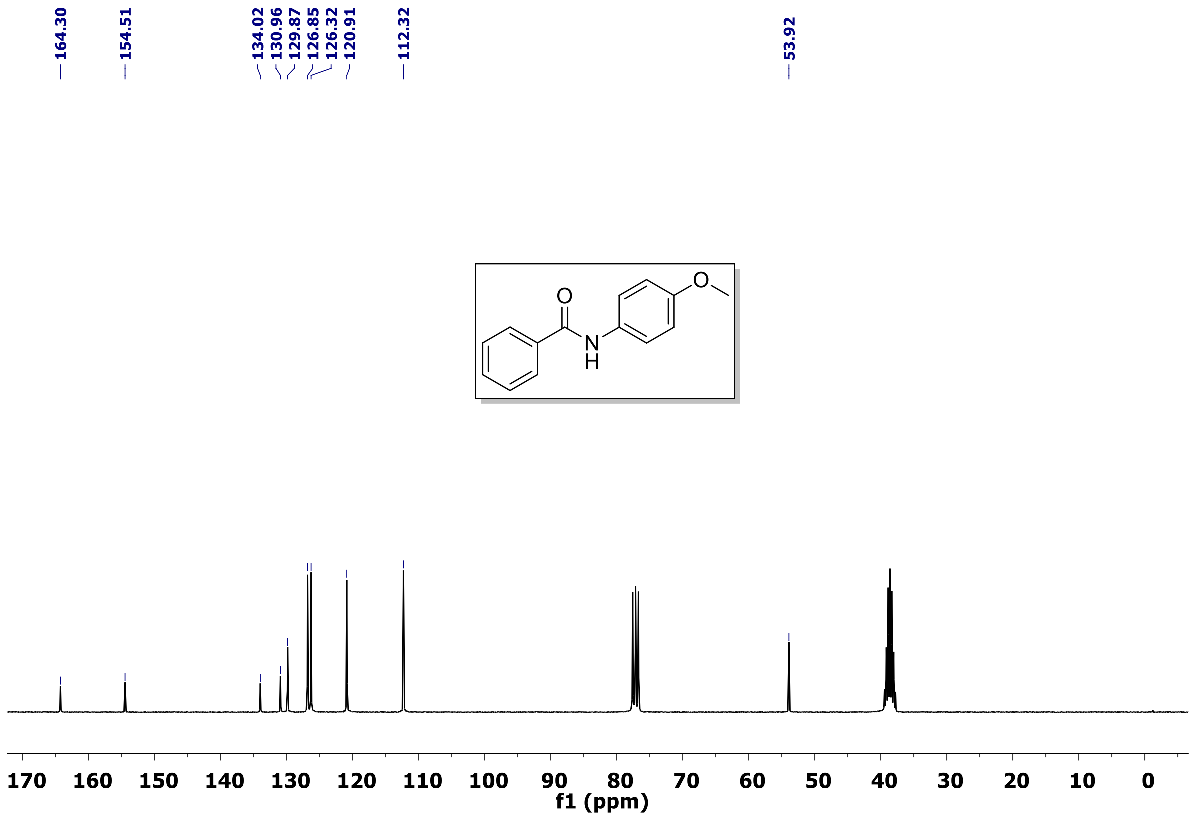

Copies of ^{1}H NMR and ^{13}C NMR spectra of **7c**

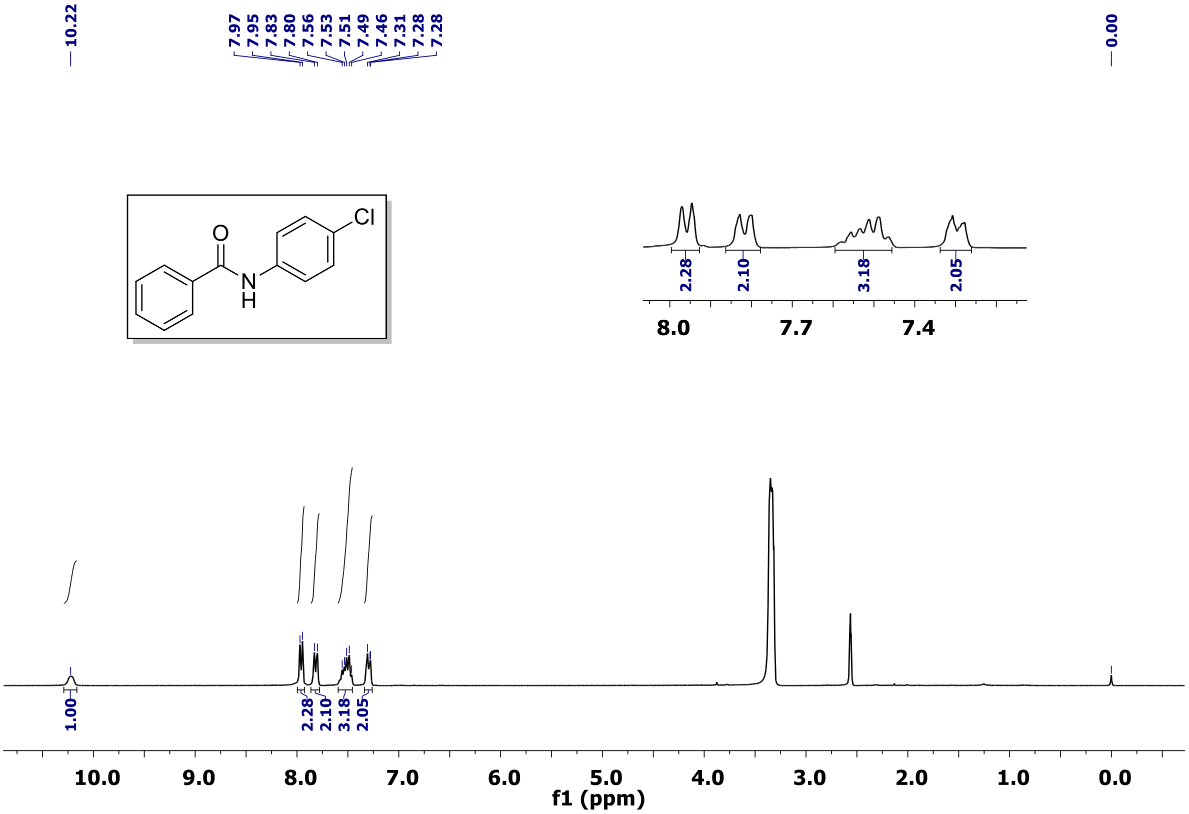

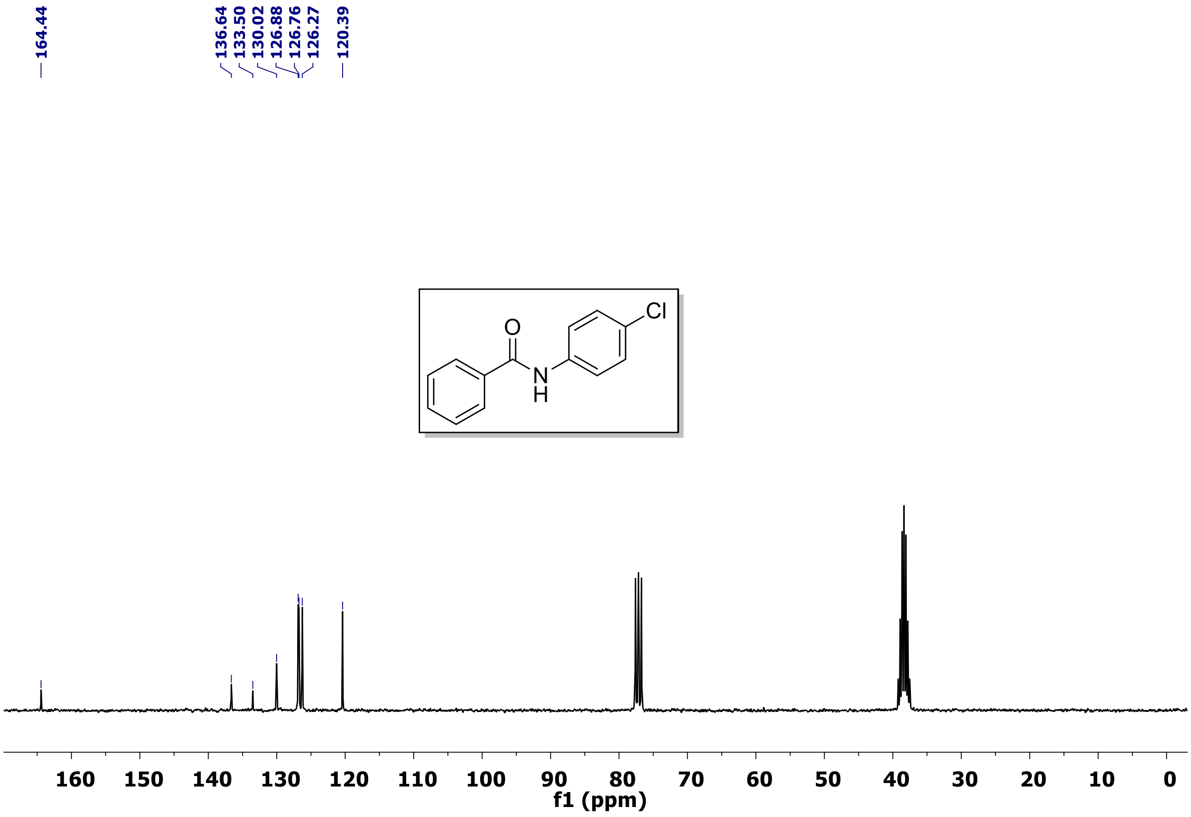

Copies of ^{1}H NMR and ^{13}C NMR spectra of **7d**

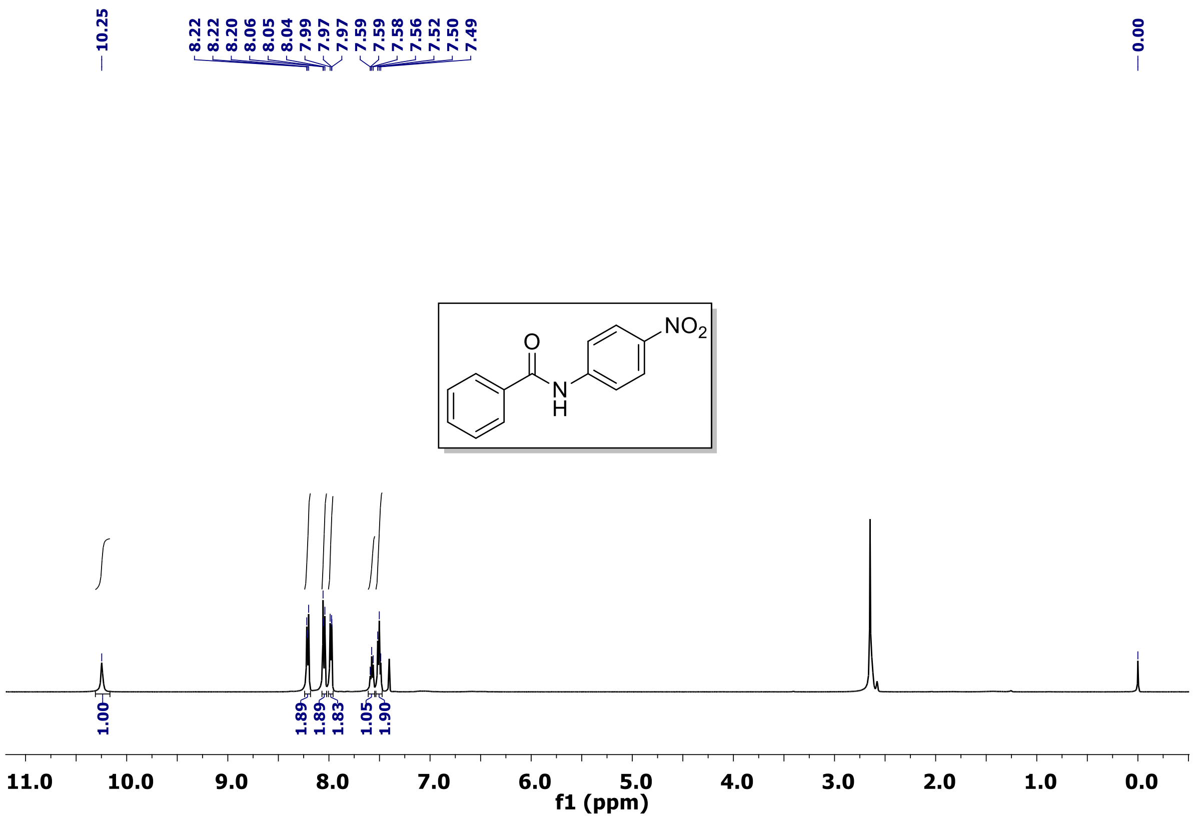

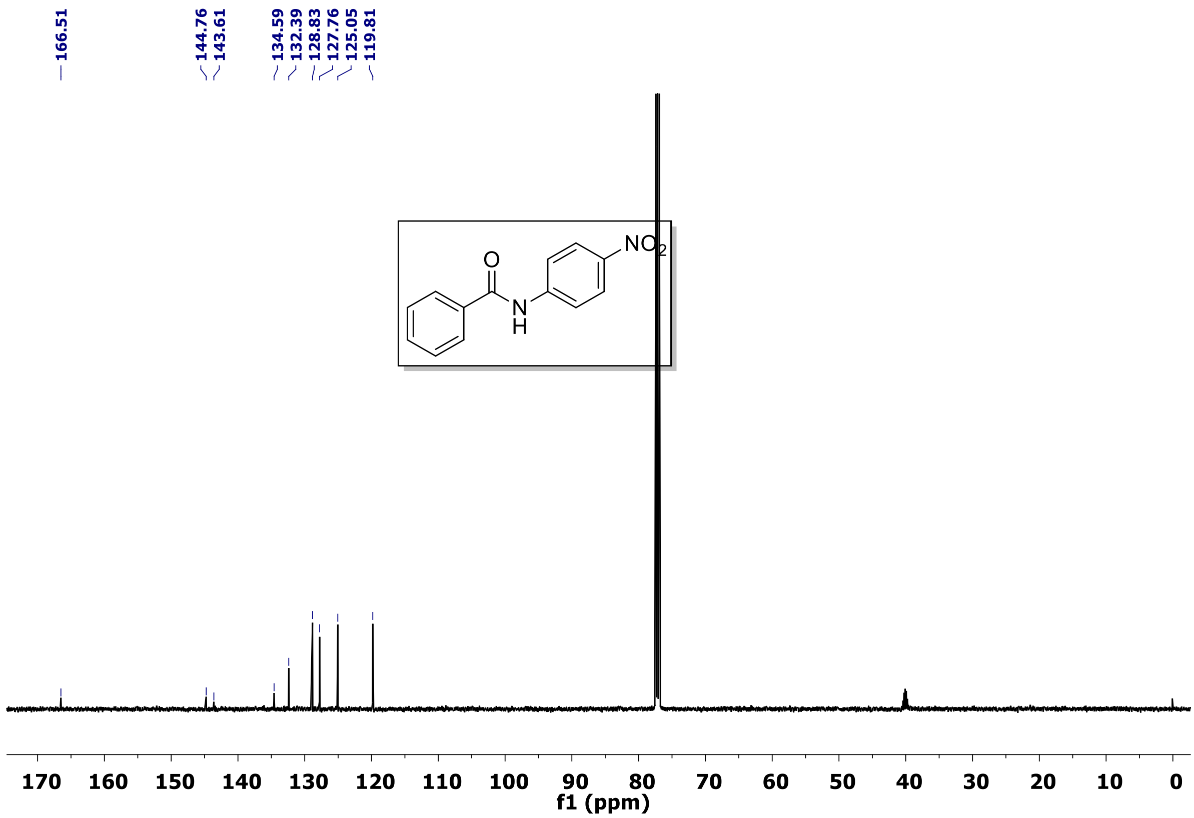

Copies of ^{1}H NMR and ^{13}C NMR spectra of **7e**

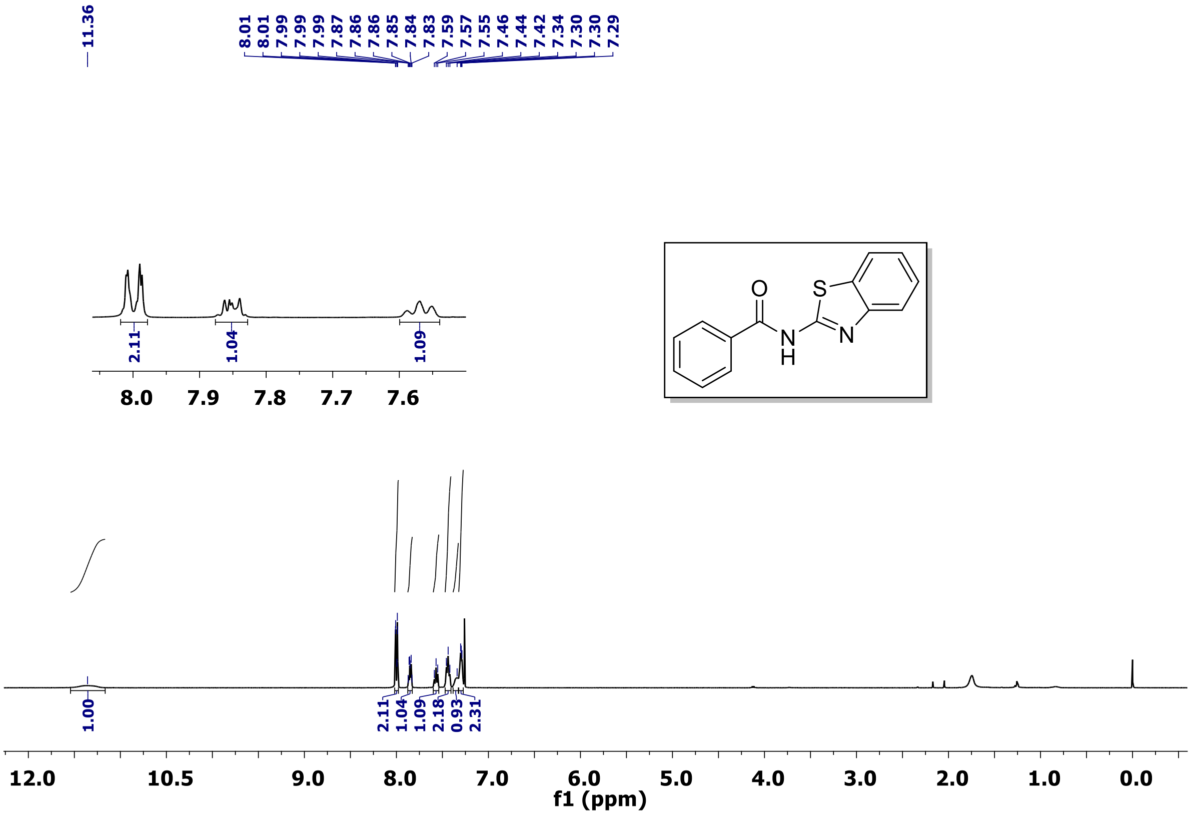

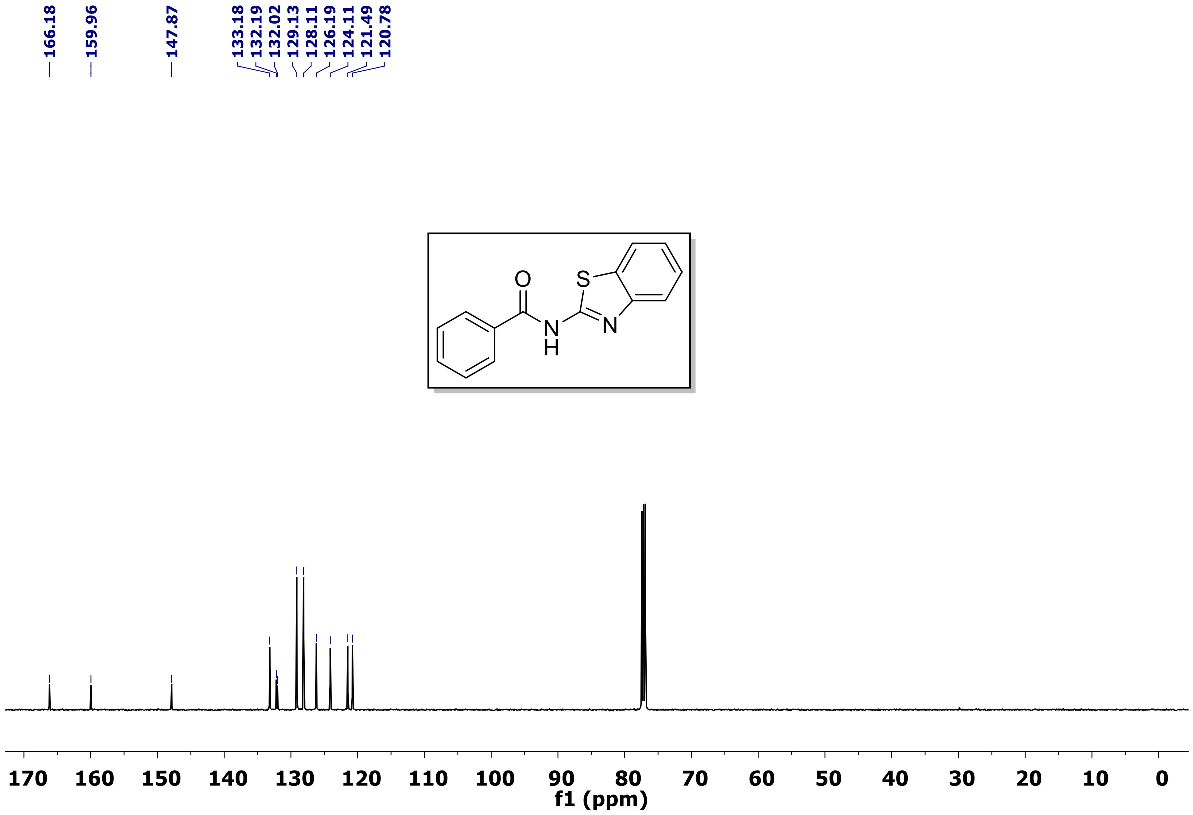

Copies of ^{1}H NMR and ^{13}C NMR spectra of **7f**

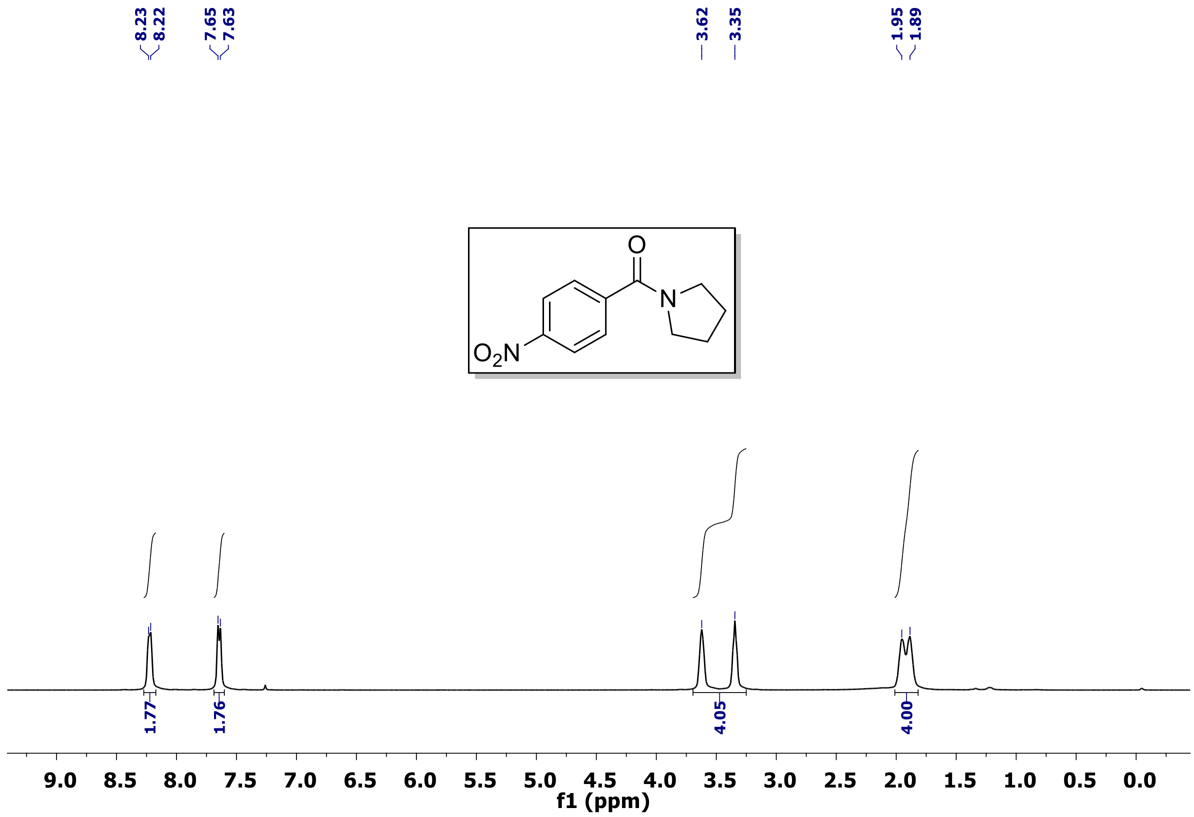

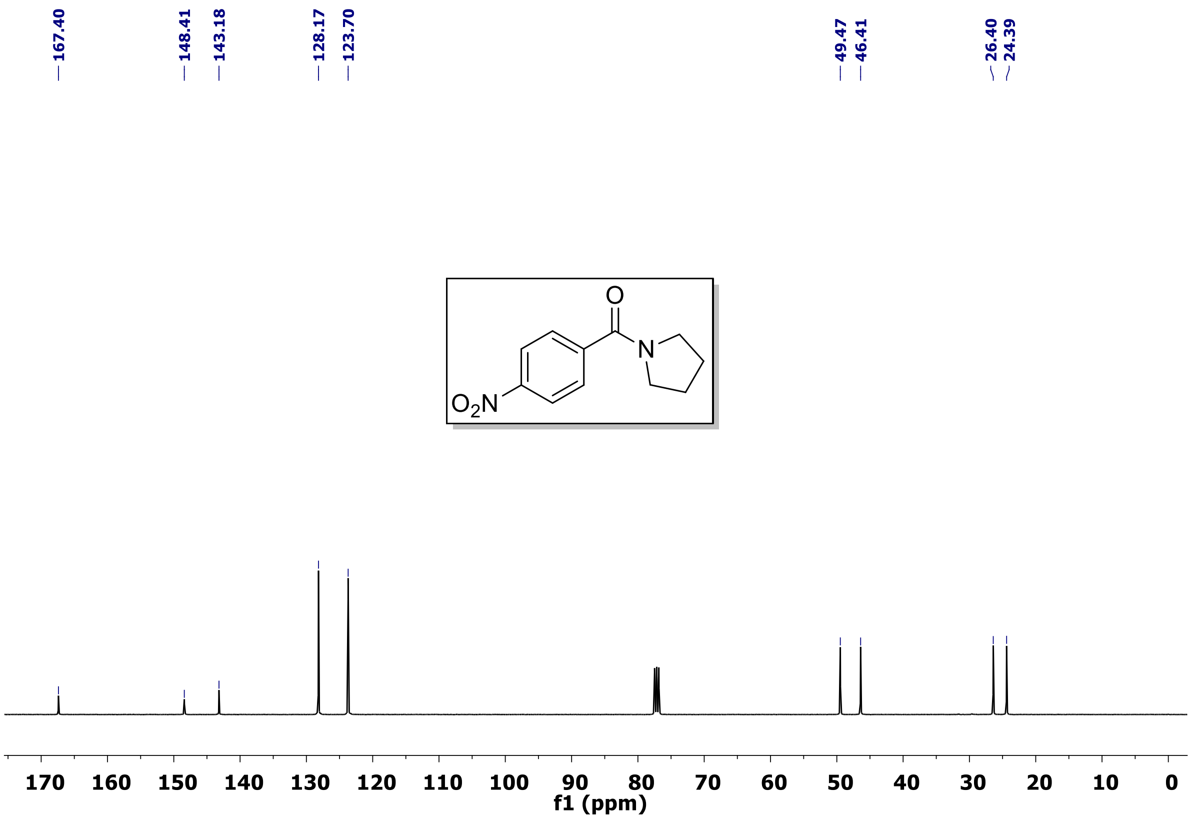

Copies of ^{1}H NMR and ^{13}C NMR spectra of **7g**

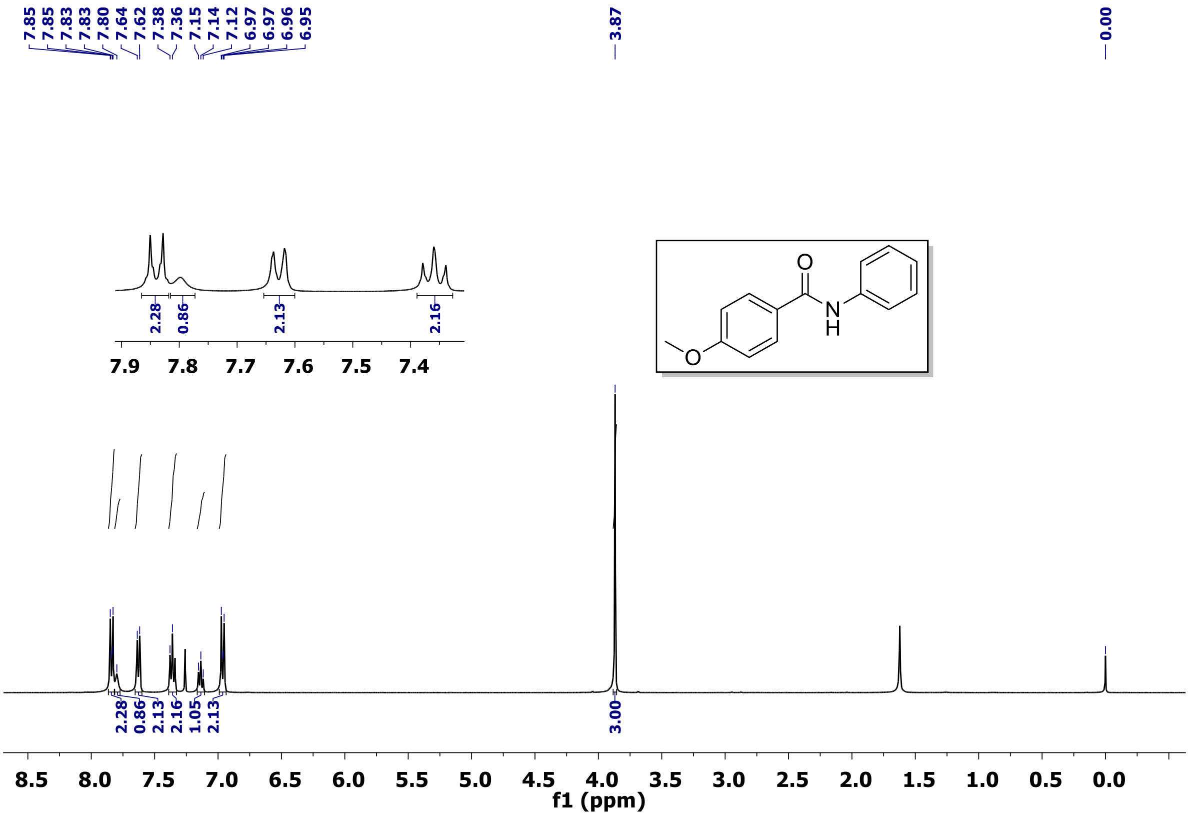

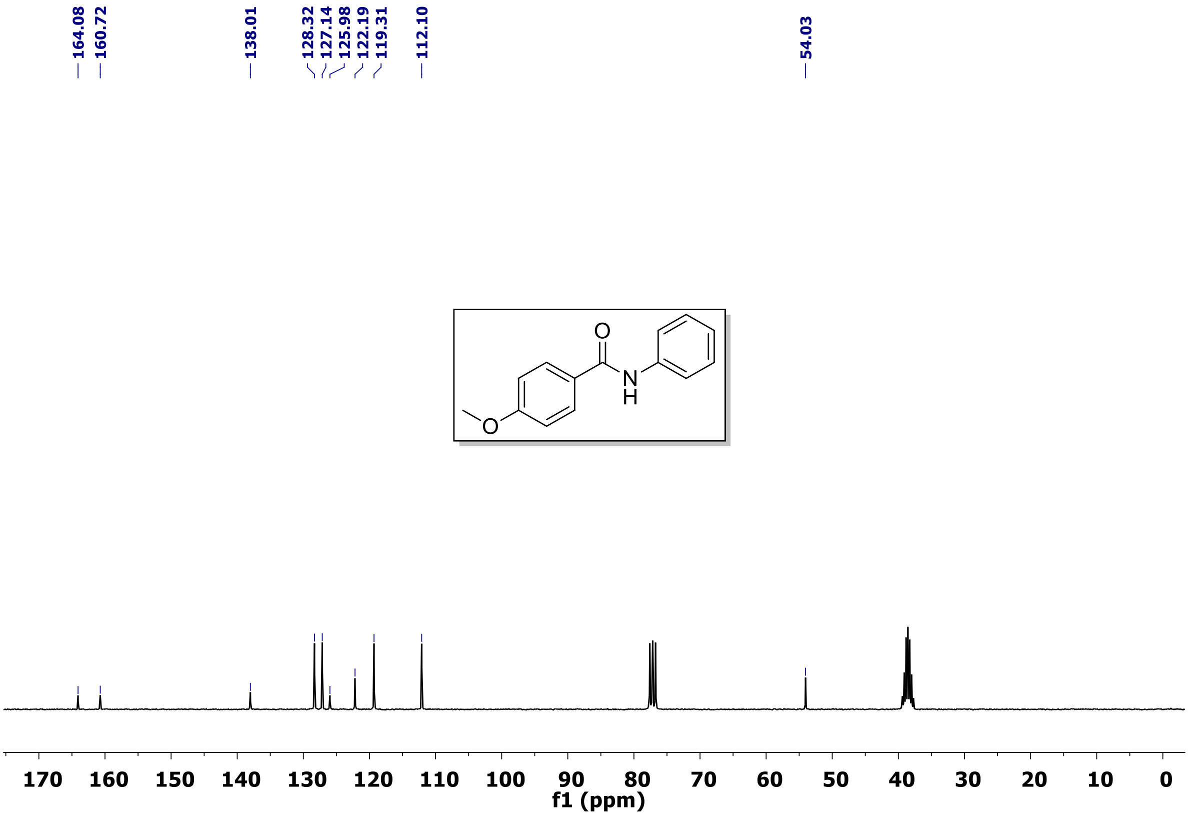

Supplement: File 1 — Experimental procedures, characterization data and copies of 1H and 13C NMR spectra of the compounds. [file Beilstein_J_Org_Chem-15-1864-s001.pdf]
